# Absolute Affinities from Quantitative Shotgun Glycomics Using Concentration-Independent (COIN) Native Mass Spectrometry

**DOI:** 10.1021/acscentsci.3c00294

**Published:** 2023-06-15

**Authors:** Duong T. Bui, James Favell, Elena N. Kitova, Zhixiong Li, Kelli A. McCord, Edward N. Schmidt, Fahima Mozaneh, Mohamed Elaish, Amr El-Hawiet, Yves St-Pierre, Tom C. Hobman, Matthew S. Macauley, Lara K. Mahal, Morris R. Flynn, John S. Klassen

**Affiliations:** † Department of Chemistry, University of Alberta, Edmonton T6G 2G2, Alberta, Canada; ‡ Department of Cell Biology, University of Alberta, Edmonton T6G 2H7, AB, Canada; § Poultry Diseases Department, Faculty of Veterinary Medicine, Cairo University, Giza 12211, Egypt; ∥ Department of Pharmacognosy, Faculty of Pharmacy, Alexandria University, Alexandria 21561, Egypt; ⊥ Institut National de la Recherche Scientifique (INRS), INRS-Centre Armand-Frappier Santé Biotechnologie, Laval H7 V 1B7, QC, Canada; # Department of Medical Microbiology and Immunology, University of Alberta, Edmonton T6G 2E1, AB, Canada; ∇ Li Ka Shing Institute of Virology, University of Alberta, Edmonton T6G 2E1, Alberta, Canada; ○ Department of Mechanical Engineering, Faculty of Engineering, University of Alberta, Edmonton T6G 1H9, Alberta, Canada

## Abstract

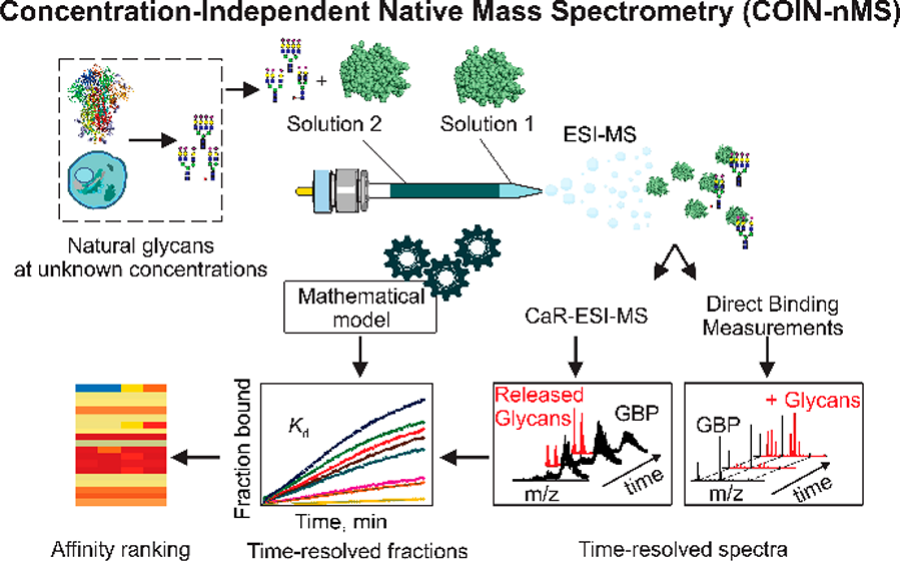

Native mass spectrometry (nMS) screening of natural glycan libraries against glycan-binding proteins (GBPs) is a powerful tool for ligand discovery. However, as the glycan concentrations are unknown, affinities cannot be measured directly from natural libraries. Here, we introduce **Co**ncentration-**In**dependent (COIN)-nMS, which enables quantitative screening of natural glycan libraries by exploiting slow mixing of solutions inside a nanoflow electrospray ionization emitter. The affinities (*K*_d_) of detected GBP–glycan interactions are determined, simultaneously, from nMS analysis of their time-dependent relative abundance changes. We establish the reliability of COIN-nMS using interactions between purified glycans and GBPs with known *K*_d_ values. We also demonstrate the implementation of COIN-nMS using the catch-and-release (CaR)-nMS assay for glycosylated GBPs. The COIN-CaR-nMS results obtained for plant, fungal, viral, and human lectins with natural libraries containing hundreds of *N*-glycans and glycopeptides highlight the assay’s versatility for discovering new ligands, precisely measuring their affinities, and uncovering “fine” specificities. Notably, the COIN-CaR-nMS results clarify the sialoglycan binding properties of the SARS-CoV-2 receptor binding domain and establish the recognition of monosialylated hybrid and biantennary *N*-glycans. Moreover, pharmacological depletion of host complex *N*-glycans reduces both pseudotyped virions and SARS-CoV-2 cell entry, suggesting that complex *N*-glycans may serve as attachment factors.

## Abstract 2

We describe a breakthrough screening technology that enables the measurement of absolute ligand affinities for glycan-binding proteins using natural libraries of glycans at unknown concentrations

## Introduction

Complex carbohydrates (glycans), covalently attached to proteins, peptides, lipids, or RNA, are found inside, secreted from, and on the surface of the cells of all living organisms.^1−3^ Glycan profiles are specific to the cell or tissue type and change in response to cellular conditions, such as aging, infection, and disease.^4−7^ Binding of glycans to glycan-binding proteins (GBPs) mediates diverse processes in health and disease, including cellular recognition and signaling, immune responses, bacterial and viral infections, and diseases such as cancers and neurodegenerative disorders.^1,2^ Identifying and characterizing glycan interactions are essential to understand the mechanisms of cellular and immunological processes and guides the development of therapeutics and diagnostics.^8,9^ However, mapping the glycan interactome—the repertoire of glycans recognized and their (relative) affinities—of GBPs is challenging due, in part, to the limited availability and high cost of glycans in their purified form. For example, it is estimated that there are ≥10^4^ human glycan determinants,^10^ but the available libraries of purified glycans typically contain only a few hundred structures. The influence of presentation (e.g., as glycolipids, glycopeptides, or glycoproteins), environment (e.g., membrane composition), and the low affinities (typically *K*_d_ > μM) of glycan-GBP interactions further hinder their discovery and characterization.^11,12^

Shotgun glycomics (SG), which utilizes glycans extracted from natural sources, such as tissue, cell cultures, or biofluids, enables screening of biologically focused libraries and expands the size and diversity of glycan libraries without requiring expensive and experimentally challenging chemical or chemo-enzymatic synthesis.^13^ Shotgun glycomics screening is usually performed using glycan microarrays constructed from fractionated natural glycan libraries. This powerful technique has enabled discovery of glycan ligands for both endogenous and exogenous GBPs.^14−16^ However, microarray screening is not quantitative (does not measure absolute *K*_d_) and has limitations, such as binding artifacts associated with GBP and glycan modifications and immobilization, and false negatives for ligands with fast off kinetics, typical of low-affinity interactions, during washing steps.^17−19^ Moreover, the purification of glycans from natural libraries is often incomplete, leading to mixtures of structures on the array. As a result, glycan specificities deduced from different arrays often exhibit inconsistencies.^17−19^ Consequently, there is a significant need for quantitative, label- and immobilization-free SG screening methods.

Native mass spectrometry (nMS)—normally implemented with electrospray ionization (ESI)-MS under native-like solution conditions with experimental/instrumental parameters that preserve the noncovalent interactions present in solution—is an attractive alternative to glycan array SG screening. Mass spectrometry-based SG can be implemented using direct ESI-MS (nMS) detection of GBP–glycan interactions or using catch-and-release (CaR)-ESI-MS (referred to here as CaR-nMS), whereby ligands are detected following their release (as ions) from GBP–glycan complexes upon collisional activation in the gas phase.^20^ As there is no need to label the glycans or GBP, nMS-based screening is free of binding artifacts resulting from chemical modifications and glycan immobilization. Direct nMS screening requires GBP–glycan complexes to be at least partially resolved for identification and quantification. This is difficult to achieve for glycan binding to high molecular weight (MW) or heavily glycosylated GBPs. The analytical challenge is even greater for natural glycan libraries, which may contain hundreds of species. In such cases, the CaR-nMS assay, which relies on the detection of released glycan ligands, has clear advantages.

Because the concentrations of glycans in natural libraries are unknown and challenging to measure, neither absolute nor relative affinities can be directly determined from nMS or CaR-nMS measurements performed on natural libraries. There have been attempts to establish relative affinities of ligands in natural libraries by combining relative ligand abundances measured by CaR-nMS with their relative concentrations, established by liquid chromatography (LC) analysis of the library following fluorophore labeling.^21^ However, a recent investigation into the robustness of this approach revealed differences, in some cases dramatic, in affinity rankings.^22^ These differences were traced to nonuniform and fluorophore-dependent glycan recovery efficiencies (in the purification steps required after labeling) and nonuniform changes in solution affinities and ligand release efficiencies (from GBPs in CaR-nMS).^22^ Together, these results reinforce the critical need for a label-free approach for quantitative nMS-SG screening.

Here, we introduce **Co**ncentration **In**dependent (COIN)-nMS for quantitative screening of mixtures of glycans of unknown concentration against GBPs. The method, inspired by the recently developed Slow Mixing Mode nanoflow ESI (nanoESI)-MS (SLOMO) technique,^23^ exploits slow mixing of solutions inside a nanoESI emitter to achieve a nearly constant glycan concentration flux. The *K*_d_ of detected interactions is determined from analysis of the time-dependent changes in the relative abundances of GBP–glycan complexes and the glycan concentration flux. We first demonstrate the reliability of COIN-nMS through affinity measurements performed on individual GBP-glycan interactions with known *K*_d_ and then apply the method to screen defined mixtures of glycans with known GBP affinities. Because GBP glycosylation can hinder direct detection of glycan interactions as the peaks are not easily resolved, we also describe the implementation of COIN-nMS using the CaR-nMS assay (COIN-CaR-nMS) and use this technique to quantitatively screen natural glycan and glycopeptide libraries against a series of lectins, including human immune lectins and viral and plant lectins. Together, the screening results, and the new insights into the fine glycan specificity of GBPs they provide, highlight the tremendous power of COIN-nMS/CaR-nMS for quantitative SG screening and mapping the glycan interactome of GBPs relevant to human health and disease as well as lectins used in biochemical and bioanalytical assays and diagnostics.

## Materials and Methods

### Protein and Oligosaccharides

#### Proteins

The fragment of the C-terminus of the carbohydrate recognition domain of human Galectin-3 (GAL-3C, residues 107–250, molecular weight (MW) 16 327 Da) was a gift from Professor Chris Cairo (University of Alberta). The lectin *Sambucus nigra agglutinin* (SNA) was purchased from MJS BioLynx, Inc. (Brockville, Canada); the lectin mixture *Maackia amurensis agglutinin* (MAA) was purchased from Sigma-Aldrich Canada (Oakville, Canada). A fragment of human Siglec 2 (fCD22, residue 1–332, MW 37.4 kDa), CD22-Fc chimera (CD22-Fc, MW 150 kDa), Siglec 7-Fc (Sig7-Fc, residue 1–345, MW 150 kDa) cloned in frame with human IgG1 Fc and a C-terminal His6, were expressed in wild-type CHO cells, as described elsewhere.^24^ The carbohydrate recognition domain (CRD) of DC-SIGN (residues 250–404, MW 17 794 Da) was prepared as described elsewhere.^25,26^ Human galectin-7 (GAL-7, monomer MW 14.94 kDa) was produced as described previously.^23^ The receptor binding domain (RBD, residues 319–541, MW 32 kDa) of the spike protein of severe acute respiratory syndrome coronavirus 2 (SARS-CoV-2) was a gift from Professor Stephen M. Tompkins (University of Georgia). Bovine fetuin (BF), asialo bovine fetuin (aBF), human lactoferrin (LF), and bovine alpha-1 acid glycoprotein (bAGP) were purchased from Sigma-Aldrich Canada (Oakville, Canada). *Aleuria Aurantia* lectin (AAL), *Phaseolus vulgaris erythroagglutinin* (PHA-E), *Ricinus communis agglutinin* (RCAI), and human transferrin (TF) were purchased from the Vector Laboratories (Newark, CA, USA). Prostate-specific Antigen (PSA, MW 28 430 Da, purified from human seminal plasma) was purchased from LEE Biosolutions (Maryland Heights, MO). To prepare stock solutions, each protein was dialyzed and concentrated in 200 mM ammonium acetate buffer (pH 6.9) using Amicon Ultra-0.5 mL centrifugal filters (EMD Millipore, Billerica, MA, USA) with 10 kDa MW cutoff. The protein concentration was estimated by UV absorption at 280 nm. All protein stock solutions were stored at −20 °C until used.

### Purified Glycans

The chemical structures and MWs of the purified glycans used in this work (**G1**-**G34**) are provided as Supporting Information (Table S1). The glycans **G1**, **G3**, **G6-G8**, **G10-G13**, **G17**, **G18**, and **G24**-**G33** were purchased from Elicityl SA (Crolles, France), **G2**, **G9**, **G21**, and **G22** from Glycom (Hørsholm, Denmark), **G5** and **G14-G16** from Omicron Biochemicals Inc. (South Bend, Indiana, US), and **G19** and **G20** from Dextra (Reading, UK). **G34** was purchased from the National Institute of Standards and Technology (Maryland, USA). **G4** was a gift from Professor Chantelle J. Capicciotti (Queen’s University). **G23** and **G25** were produced as described previously.^24^*N*-Acetyl-d-neuraminic acid (Neu5Ac) and ^13^C-Neu5Ac were purchased from Omicron Biochemicals Inc. (South Bend, IN, USA), and D3–3′-sialyllactose (D3–3SL) was produced as described in Supporting Information. Procainamide was purchased from Sigma-Aldrich Canada (Oakville, Canada).

### N-Glycan and Glycopeptide Libraries

The libraries of N-linked glycans used for nMS-SG were produced from purified glycoproteins (BF, LF, bAGP, and RBD) using PNGase F (New England Biolab, Massachusetts, United States) digestion. Glycopeptide libraries were produced from BF, LF, bAGP, and RBD using Pronase (Roche Diagnostics GmbH, Mannheim, Germany) digestion. Details of the experimental methods used for library production and glycan/glycopeptide identification by ESI-MS and hydrophilic interaction ultrahigh performance liquid chromatography (HILIC-UHPLC) coupled with fluorescence and ESI-MS detection are given as Supporting Information. Where indicated, the *N*-glycan and glycopeptide libraries were treated with neuraminidase S (NeuS, New England BioLabs, MA, USA), which selectively cleaves α2–3-linked sialic acid.^27^ A library of *N*-linked glycans, wherein all of the Neu5Ac is α2–3-linked, was prepared from aBF, as described in Supporting Information.

### Mass Spectrometry

Measurements were performed on a Q-Exactive Orbitrap mass spectrometer (Classic) and a Q-Exactive Ultra-High Mass Range (UHMR) Orbitrap mass spectrometer (Thermo Fisher Scientific, Bremen, Germany). Both instruments were equipped with a modified nanoflow ESI source. Details of experimental and instrumental parameters and data analysis are provided as Supporting Information.

### Pseudotyped Virus Production and Transduction

Details on the production and transduction of the pseudovirus with pharmacological modulation of *N*-glycan type are given as Supporting Information.

### SARS-CoV-2 Viral Infection Assays

All experiments with SARS-CoV-2 virus were performed under biosafety level 3 (BSL3) conditions on ACE2^+^ HEK293 cells. Details are given as Supporting Information.

## Results and Discussion

### Theoretical Overview of COIN-nMS Affinity Measurements

Native MS affinity measurements rely on the accurate determination of the distribution of interacting species at known initial concentrations. For example, the dissociaton constant (*K*_d_) for a 1:1 GBP (P)-ligand (L) interaction (eq 1) can be expressed by eq 2
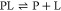
1
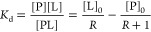
2where [P]_0_ and [L]_0_ are the initial concentrations of P and L, respectively. Assuming PL and P have similar ESI-MS response factors,^28^ the ratio (*R*) of their concentrations at equilibrium in solution can be calculated from the ratio of the total abundance (Ab) of the corresponding gas-phase ions measured by ESI-MS, eq 3.
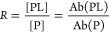
3

A unique feature of nMS affinity measurements is that *K*_d_ can be determined from a single measurement. However, for weak (large *K*_d_) interactions, a titration approach is commonly used (fixed [P]_0_ and varying [L]_0_), and *K*_d_ is determined by fitting eq 4a to a plot of fraction of protein bound to ligand (*F*) versus [L]_0_
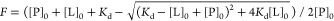
4awhere *F* is calculated from eq 4b.
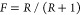
4b

In the case where multiple ligands are present together, the affinity (*K*_d,Lx_) of a given ligand (L_*x*_) can be expressed as eq 5
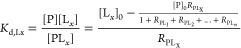
5where *R*_PL_*x*__ is obtained from eq 6.
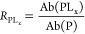
6

**Figure 1:**
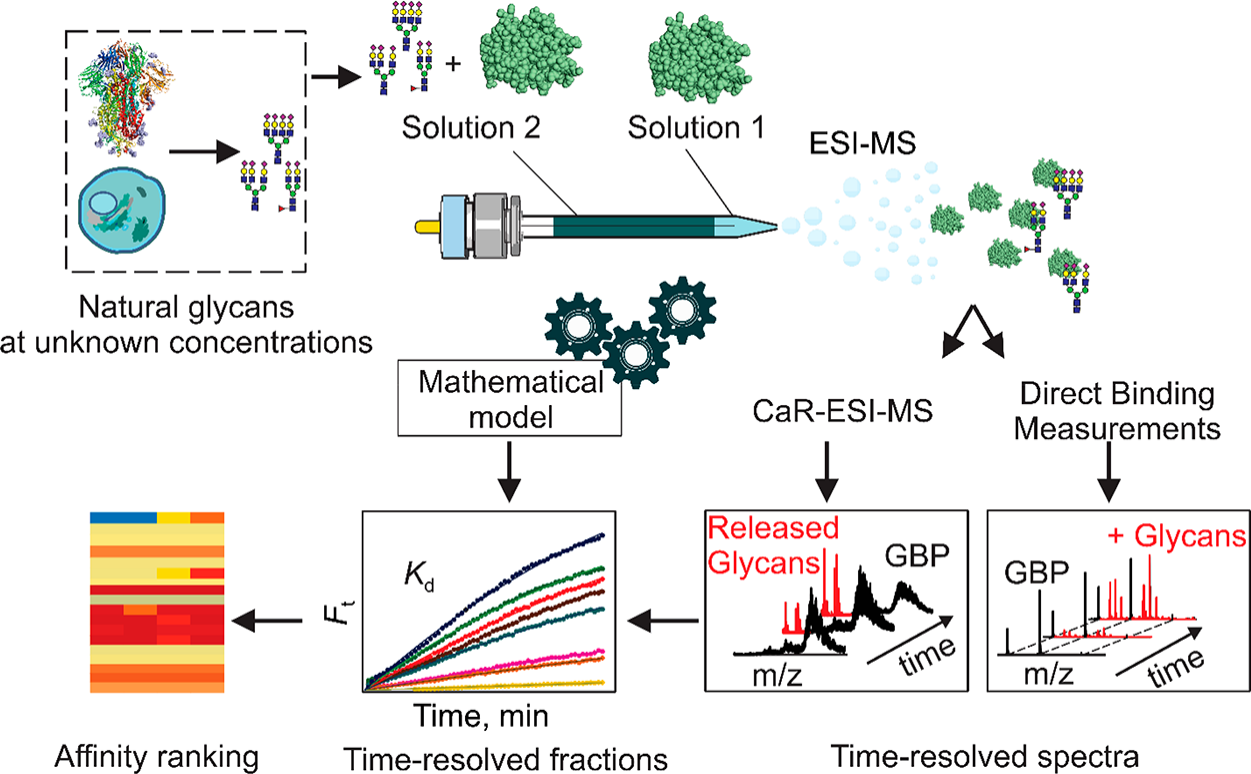
Overview of the **Co**ncentration-**In**dependent native mass spectrometry (COIN-nMS) workflow for quantitative screening of natural glycan libraries against GBPs. Two solutions (*Solution 1* and *2*) are loaded, in a layered fashion, into a nanoESI tip. Both *Solution 1* and *2* contain GBP, at the same concentration; *Solution 2* also contains the glycan library at unknown concentration. Specific binding of GBP to components of the glycan library is measured directly by time-resolved native mass spectrometry (nMS), whereby gaseous ions corresponding to intact GBP-glycan complexes are detected, or by catch and release nMS (CaR-nMS), whereby ligands released (collisionally) from GBP-glycan complexes are detected. Time-dependent fractional abundance of ligand-bound GBP (*F*_t_) is calculated from the relative abundances of free and ligand-bound GBP. The affinity (*K*_d_) is calculated by fitting eq 12 (COIN-nMS) or eq 13 (COIN-CaR-nMS) to the corresponding *F*_t_ data. The glycan binding specificity (affinity ranking) of the GBP is established by comparing *K*_d_ for the different glycan ligands detected.

The reliability of nMS for measuring the *K*_d_ of GBP–glycan complexes has been rigorously tested and, when performed using appropriate solution and instrumental conditions, shown to be in good agreement with values measured by isothermal titration calorimetry (ITC).^29^ However, it is not possible to calculate *K*_d_ directly from nMS binding data acquired at an unknown ligand concentration. In principle, *K*_d_ and [L]_0_ can be determined from nonlinear regression of titration data acquired using serial dilution of a solution of unknown [L]_0_. However, it is challenging to obtain a meaningful *K*_d_ from real (nonideal) binding data without imposing constraints. The COIN-nMS technique, which was inspired by the slow mixing mode (SLOMO) nanoESI-MS method,^23^ effectively overcomes many of the practical limitations of the nMS-based serial dilution approach. The assay relies on the continuous monitoring of P and the corresponding L-bound complex(es) under conditions of slow solution mixing. To implement COIN-nMS for a 1:1 PL complex, two different solutions (*Solutions 1* and *2*) are loaded into a single nanoESI tip (Figure 1). In the simplest format, *Solution 1*, which contains P at concentration [P]_0_, is loaded first, followed by *Solution 2*, which contains P ([P]_0_) and ligand ([L]_0_). As described below, the concentration of ligand at the end of the nanoESI tip (which is initially zero) increases with time (*t*) due to mixing, eq 7
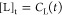
7where *C*_L_(*t*) is the *t*-dependent function that describes the change in ligand concentration due to diffusion and advection.

The time-dependent fractional binding site occupancy (fraction bound, *F*_t_) of P is calculated from the time-dependent abundance ratio (*R*_t_) of ligand-bound and free P ion signal (Ab_t_(PL) and Ab_t_(P), respectively), eqs 8a–8c.
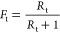
8a
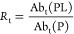
8b
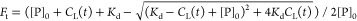
8c

### Glycan Concentration Flux in a nanoESI Emitter

**Figure 2:**
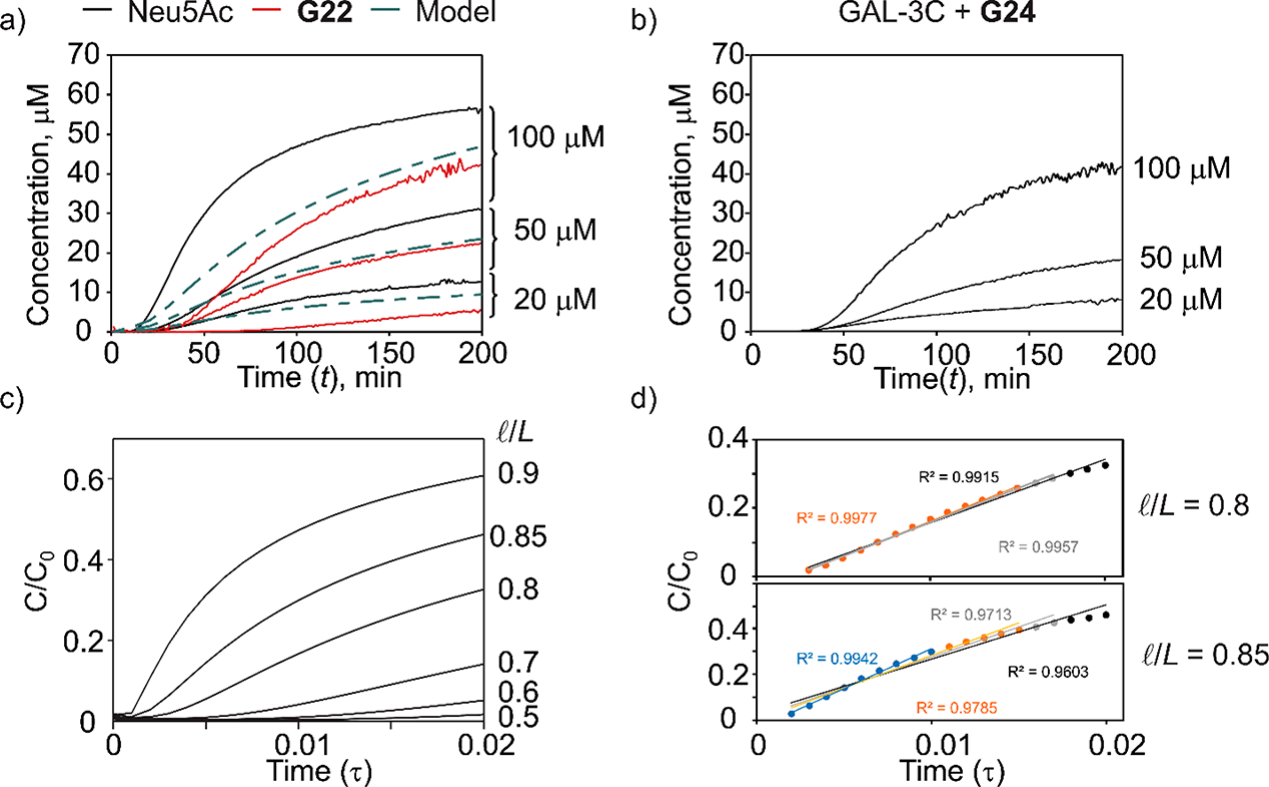
Glycan concentration flux in COIN-nMS experiments. (a**)** Time-dependent concentration of Neu5Ac and 3SL (**G22**) measured by nMS. *Solution 1* contained only internal standard (20 μM of ^13^CNeu5Ac or D3–3SL), and *Solution 2* contained internal standard (20 μM) and Neu5Ac or **G22** (20, 50, or 100 μM). Dashed line corresponds to model as described in text and as Supporting Information. (b) Time-dependent concentration of **G24** in the presence of GAL-3C measured by nMS. *Solution 1* contained only GAL-3C (5 μM), and *Solution 2* contained GAL-3C (5 μM) and **G24** (20, 50, or 100 μM). (c) Time-dependent (expressed as dimensionless time, τ) relative concentrations at the end of the capillary calculated at different volume ratios (*l*/L = 0.5, 0.6, 0.7, 0.8, 0.85, 0.9). (d) Demonstration of using a linear model to describe the diffusion data at different time ranges with *l*/L = 0.8 and 0.85.

To determine *K*_d_ from *F*_t_, it is necessary to have a model that reasonably describes *C*_L_(*t*) in a COIN-nMS experiment. To understand how the glycan concentration changes during COIN-nMS, the time-dependent concentrations of Neu5Ac and 3′-sialyllactose (3SL ≡ **G22**) in a nanoESI emitter were monitored using stable isotope-labeled internal standards (^13^CNeu5Ac or D3–3SL) under mixing conditions. The experiments were performed using a *Solution 1* that contained only internal standard and a *Solution 2*, which contained internal standard (at the same concentration as *Solution 1*) and Neu5Ac or **G22**. The resulting time-dependent concentration plots are shown in Figure 2a. Analogous experiments were performed on **G24** in the presence of GAL-3C (*Solution 1* contained GAL-3C, and *Solution 2* contained GAL-3C and **G24**), which binds to **G24** with a *K*_d_ of 7.2 ± 0.4 μM,^22^ to assess the effects of GBP binding. The concentration of **G24** was calculated using the known *K*_d_ and *R* at each time point (Figure 2b). Notably, the Neu5Ac and **G22** concentration curves have similar appearances (Figure 2a). The onset of glycan signal is approximately 15–20 min after solution introduction into the tip and decreased with increasing glycan concentration. For the same solution concentrations, the Neu5Ac signal appears earlier than that of **G22**, consistent with the monosaccharide having a larger diffusion coefficient (*D*) than the trisaccharide. The time-dependent concentration curves for **G24** measured in the presence of GAL-3C are qualitatively similar to those measured for Neu5Ac and **G22** (Figure 2b); the later signal onsets are consistent with a smaller “apparent” *D* of **G24** due to a fraction of the glycan being in the bound (to GAL-3C) form.

The factors affecting analyte mixing inside a nanoESI emitter have not been systematically investigated. However, it was suggested that diffusion, as opposed to advection, dominates mixing in SLOMO experiments.^23^ To better understand the mixing process, net analyte mass transfer within an emitter of length *L* and constant internal diameter (no taper) was modeled theoretically. The analytical model solves the advection-diffusion equation in the limit of a small Peclet number. In turn, the model predictions give important insights into the qualitative and quantitative trends measured experimentally. A description of the model and its derivation are given as Supporting Information (File 2, Theoretical Modeling). Shown in Figure 2c are the time-dependent (expressed in terms of dimensionless time, τ) changes in relative concentration at the end of the capillary corresponding to different volume ratios (0.5–0.9) of *Solution 2* (*V*_Solution 2_) to total solution volume (*V*_Total_), eq 9a
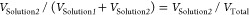
9awhich can be expressed as a length ratio, eq 9b
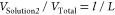
9bwhere *l* is the length of *Solution 2*. As described in Supporting Information, τ is related to *t*, *L*, and *D* by eq 10.
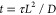
10

Theoretical curves, corresponding to a maximum mixing time of 200 min, an *L* of 2.8 cm, and *l*/*L* of 0.85 (calculated based on a total volume of 10 μL and assuming no tip taper) and using a *D* of 5 × 10^–6^ cm^2^/s, which is the reported value for glucose (Glc),^30^ are overlaid with the experimental curves (Figure 2a). Overall, the theoretical curves are similar in appearance to the experimental data but underestimate somewhat the rate of concentration increase near the onset region. This difference is most likely attributable to the tapering of the emitter and, possibly, the contribution of advection to mixing, which, for pragmatic reasons, was assumed to be small in the theoretical model.

As shown in Figure S1, select regions of the time-dependent concentration curves can be reasonably approximated using linear, logarithmic, or quadratic models allowing, in principle, *C*_L_(*t*) to be determined.^31^ However, the logarithmic and quadratic models require fitting additional parameters, which, in the absence of constraints, introduces uncertainty to *K*_d_. Ultimately, a linear model (eq 11a) was selected to describe the time dependence of the ligand concentration at the end of the emitter at early mixing times
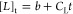
11a
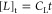
11bwhere *b* is the time-axis intercept. When the concentration change is considered from the point of mixing onset (mixing starting point), *b* becomes 0, and eq 11a reduces to eq 11b. It follows that eq 8c can be expressed as eq 12
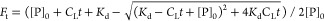
12where *C*_L_, the rate of concentration change, is unknown but constant. Fitting of eq 12 to the experimental *F*_t_ allows the values of *K*_d_ and *C*_L_ to be obtained from nonlinear least-squares regression.

Both the experimental data and theoretical modeling results were considered to identify the optimal experimental conditions for implementing COIN using the linear approximation. Higher *l*/*L* ratios produce faster mixing but a shorter linear region; smaller ratios produce extended linear regions but longer mixing onsets, thereby increasing measurement times and the possibility of spray instability resulting from tip degradation. Based on these considerations, an *l*/*L* value of between 0.8 and 0.85 (2 and 8 μL or 1.5 and 8.5 μL of *Solution 1* and *Solution 2*, respectively) was chosen as optimal. To demonstrate that linear approximation describes well a range of data points, the linear model was fit to the theoretical curve for *l*/*L* = 0.8 and 0.85 for mixing onsets of τ = 0.010, 0.015, 0.017, and 0.020 (Figure 2d). Importantly, the model describes the theoretical solution well up to a τ of 0.02 (*R*^2^ = 0.9915) for *l*/*L* = 0.8 and 0.01 (*R*^2^ = 0.9942) for *l*/*L* = 0.85, which correspond to mixing times of 200 and 100 min, respectively.

### Validation of COIN-nMS Using Model GBP–Glycan Systems

**Figure 3:**
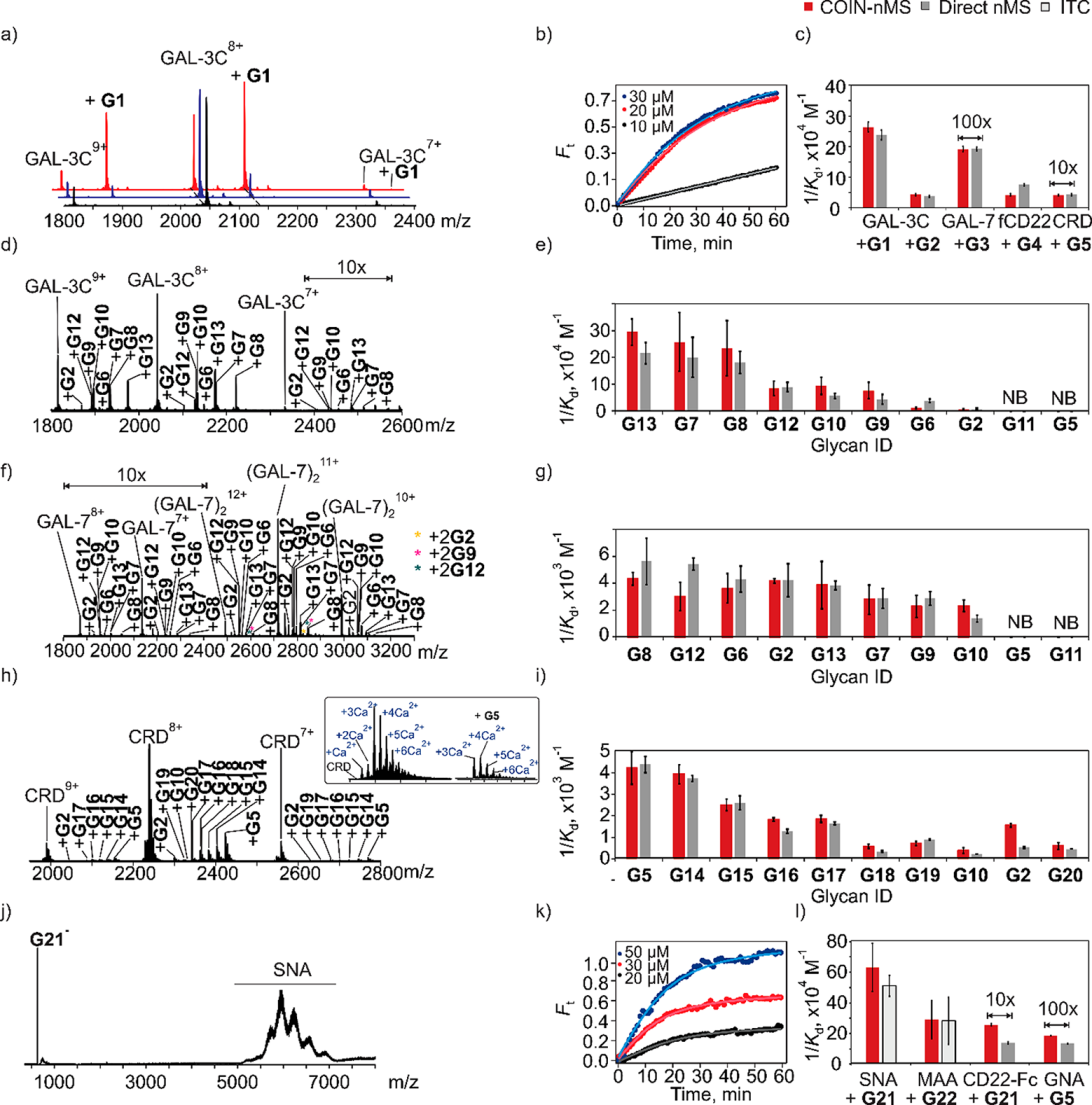
GBP-glycan affinity measurements performed using (a–i) COIN-nMS and (j–l) COIN-CaR-nMS for the model systems. (a, b) Representative time-resolved mass spectra and fractional binding site occupancy (*F*_t_) measured for GAL-3C (5 μM, *Solutions 1* and *2*) with **G1** (20 μM, *Solution 2*). (c) Comparison of the values obtained by COIN-nMS (red) vs nMS (gray). (d–i) GBP-glycan affinity measurements performed using COIN-nMS and mixtures of glycans. Representative ESI mass spectra acquired in positive ion mode (d) GAL-3C (5 μM) and **G2**, **G5**, **G6-G13**, (f) GAL-7 (5.5 μM) and **G2**, **G5**, **G6-G13**, (h) the carbohydrate binding domain (CRD) of DC-SIGN (2 μM) and **G2**, **G5**, **G10**, **G14**-**G20**. Insets show magnified region of the mass spectra containing signal for DC-SIGN CRD and (CRD+**G5**) ions. (e, g, i) Comparison of the values obtained by COIN-nMS vs nMS. (j–l) GBP-glycan affinity measurements performed using COIN-CaR-nMS. Solid lines are the best fit of eq 12 to the experimental data. (j) Representative mass spectrum measured by CaR-ESI-MS for an ammonium acetate solution (200 mM, pH 6.9) of SNA (5 μM) and **G22** (0.1 μM), collision energy (CE) 40 V. (k) Time-resolved relative abundance of released **G22** ion normalized to total SNA signal for COIN-CaR-ESI-MS experiments performed on ammonium acetate solution (200 mM, pH 6.9) of SNA (5 μM) and **G22** (0.1 μM) (*Solution 1*) and SNA (5 μM) and **G22** (20, 30, and 50 μM) (*Solution 2*). (l) Comparison of the values obtained by COIN-nMS (red) vs nMS (gray) or ITC (light gray). Solid curves represent the best fit of eq 13 to the experimental data. All measurements were performed in ammonium acetate solutions (200 mM, pH 6.9, 25 °C) except for DC-SIGN CRD (ammonium acetate, 200 mM, pH 7.4, Ca(CH_3_COO)_2_ 2.5 mM, 25 °C).

To validate COIN-nMS, the assay was used to measure glycan (individually and as mixtures) affinities for a series of immune lectins. Individual glycan affinity measurements were performed for GAL-3C with the tetrasaccharide **G1** and trisaccharide **G2**, GAL-7 with hexasaccharide **G3**, fCD22 (CD22 fragment) and the biantennary *N*-glycan **G4** and the DC-SIGN CRD with oligomannose **G5** (Figure 3a–c). Affinity measurements were performed at pH 6.9 (200 mM ammonium acetate) for GAL-3C, GAL-7, and fCD22 and pH 7.4 (200 mM ammonium acetate and 2.5 mM of Ca(CH_3_COO)_2_) for DC-SIGN CRD. For each system, *K*_d_ was determined by fitting eq 12 to the time-dependent *F*_t_ acquired from the onset of mixing (indicated as 0 min) to 60 min. Average affinities were also determined using global analysis of multiple data sets obtained at different glycan concentrations (Figure 3b and Table S2). Notably, the *K*_d_ measured by COIN agree (within 10%) with values measured directly by nMS for solutions with known initial concentrations. Moreover, the measured *K*_d_ exhibit no dependence on glycan concentration.

To demonstrate the application of COIN-nMS to glycan mixtures (Figure 3d–i), the assay was applied to small defined libraries, consisting of known binders and nonbinders at nonuniform concentrations, with GAL-3C, GAL-7, and DC-SIGN CRD. A library comprised of **G2** and **G5–13** was screened against GAL-3C (Figure 3d,e) and GAL-7 (Figure 3f,g). Of these, **G5** and **G11** do not bind to GAL-3C nor GAL-7 and served as negative controls. A library consisting of five oligomannose glycans (**G5**, **G14**-**G16**, and **G19**), three blood group antigens (**G10**, **G17**, and **G18**), one fucosylated human milk oligosaccharide (**G2**), and maltopentaose (**G20**) was screened against DC-SIGN CRD (Figures 3h,i). All of the components are known to bind, albeit weakly, to DC-SIGN CRD.^32−36^ To minimize Ca^2+^ adduct formation and nonspecific glycan binding, submicron nanoESI emitters were used for the DC-SIGN measrements.^24^ The *K*_d_ was determined by fitting eq 10 to the time-dependent *F*_t_ values acquired from the onset of mixing to 60 min. Average affinities were also determined using global analysis of multiple data sets measured at different (nonequimolar) concentrations (Tables S3–S5).

For GAL-3C and GAL-7, all but **G5** and **G11** were detected as ligands as expected. The *K*_d_ ranged from 5 to 165 μM for GAL-3C and from 178 to 730 μM for GAL-7 (Tables S2 and S3). Importantly, the affinities determined by COIN-nMS agree, within a factor of 2, with values determined directly by nMS (Figure 3e,g, respectively). For DC-SIGN CRD, all 10 of the glycans tested with COIN-nMS exhibited measurable binding (Figure 3i), with *K*_d_ ranging from 0.2 to 3 mM (Figure 3i and Table S5). Despite the affinities being relatively weak, the *K*_d_ obtained by COIN are in reasonable agreement with those obtained from direct nMS measurements. Notably, the *K*_d_ measured by COIN-nMS exhibit no dependence on glycan concentrations.

### Validation of COIN-CaR-nMS for Quantitative Library Screening

For most glycosylated GBPs, glycan binding measurements by nMS are challenging because the heterogeneity (micro- and macroheterogeniety) of GBP species makes quantifying the free and ligand-bound glycoforms difficult. As a result, detection of glycan ligands of glycosylated GBPs is usually performed using CaR-nMS. In CaR-nMS, glycan ligands bound (noncovalently) to a GBP are released as ions in the gas phase by collisional activation of the GBP-glycan complexes and detected. The results of CaR-nMS screening performed on glycan libraries of known concentrations enable GBP-glycan affinity rankings to be constructed from the relative abundances of released ligands. Generating titration curves from the relative abundances of released ligands in CaR-nMS experiments is generally not possible due to tip-to-tip variability in released ligand signal. The COIN approach enables quantification with CaR-nMS (COIN-CaR-nMS). Due to differences in the detection efficiencies (DEs) of released glycan ligands, eq 13 was used to analyze the COIN-CaR-nMS data
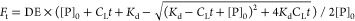
13where DE is the detection efficiency of the released glycan relative to the GBP. Because PL dissociates to free P and L in the COIN-CaR-nMS experiment, *F*_t_ is calculated from eq 14
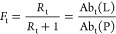
14where Ab_t_(P) and Ab_t_(L) are the time-dependent abundances of free P and L ions.

To demonstrate the feasibility of quantifying glycan binding with COIN-CaR-nMS, affinity measurements were performed on SNA and CD22-Fc with the trisaccharide **G21** (6SL), MAA with the trisaccharide **G22**, and GNA with oligomannose **G5** (Figure 3j–l). The apparent *K*_d_ for the SNA-**G21** and MAA-**G22** interactions were quantified (2.0 ± 0.3 and 4 ± 2 μM, respectively) by ITC (Figures S2 and S3), and the *K*_d_ for fCD22 to **G21** (75 ± 4 μM) and GNA to **G5** (774 ± 13 μM) were quantified by direct ESI-MS measurement. Significantly, the affinities measured using the COIN-CaR-nMS workflow are in excellent agreement with the reference values (Figure 5 and Table S6).

COIN-CaR-nMS measurements were also performed on SNA in the presence of two ligands (**G21** and **G23**) at varying relative concentrations—**G21** (30 μM) and **G23** (10 nM–10 μM). Importantly, the measured *K*_d_ were insensitive to the relative concentrations (Table S7). Similar results were obtained for binding measurements performed on Sig7-Fc and **G29** (30 μM) and **G21** (100 nM–10 μM). These findings establish that that *K*_d_ measured by COIN-CaR-nMS do not exhibit a dependence on ligand concentrations under the conditions used. Additionally, the results of these control experiments provide some insight into the dynamic range of COIN-CaR-nMS assay. Notably, it was found that a minimum ligand concentration of 50 to 500 nM is required to determine *K*_d_ for these interactions,

### Glycan Specificities of Lectins Determined with COIN-CaR-nMS

To illustrate the power of COIN-CaR-nMS for assessing the fine glycan specificities of lectins, natural *N*-glycan (total of 115 unique MWs) and glycopeptide (395 MWs) libraries (Figures S4–S7, Tables S8–S11) were prepared from purified glycoproteins and screened against a series of lectins and affinities measured. The different cut-offs of reported *K*_d_s (for different GBPs) reflect the differences in the *K*_d_ of the interactions and glycan ligand abundances. The resulting affinity rankings from COIN-CaR-nMS were compared to the reported trends in specificity from glycan array data.

### Plant and Fungal Lectins

**Figure 4:**
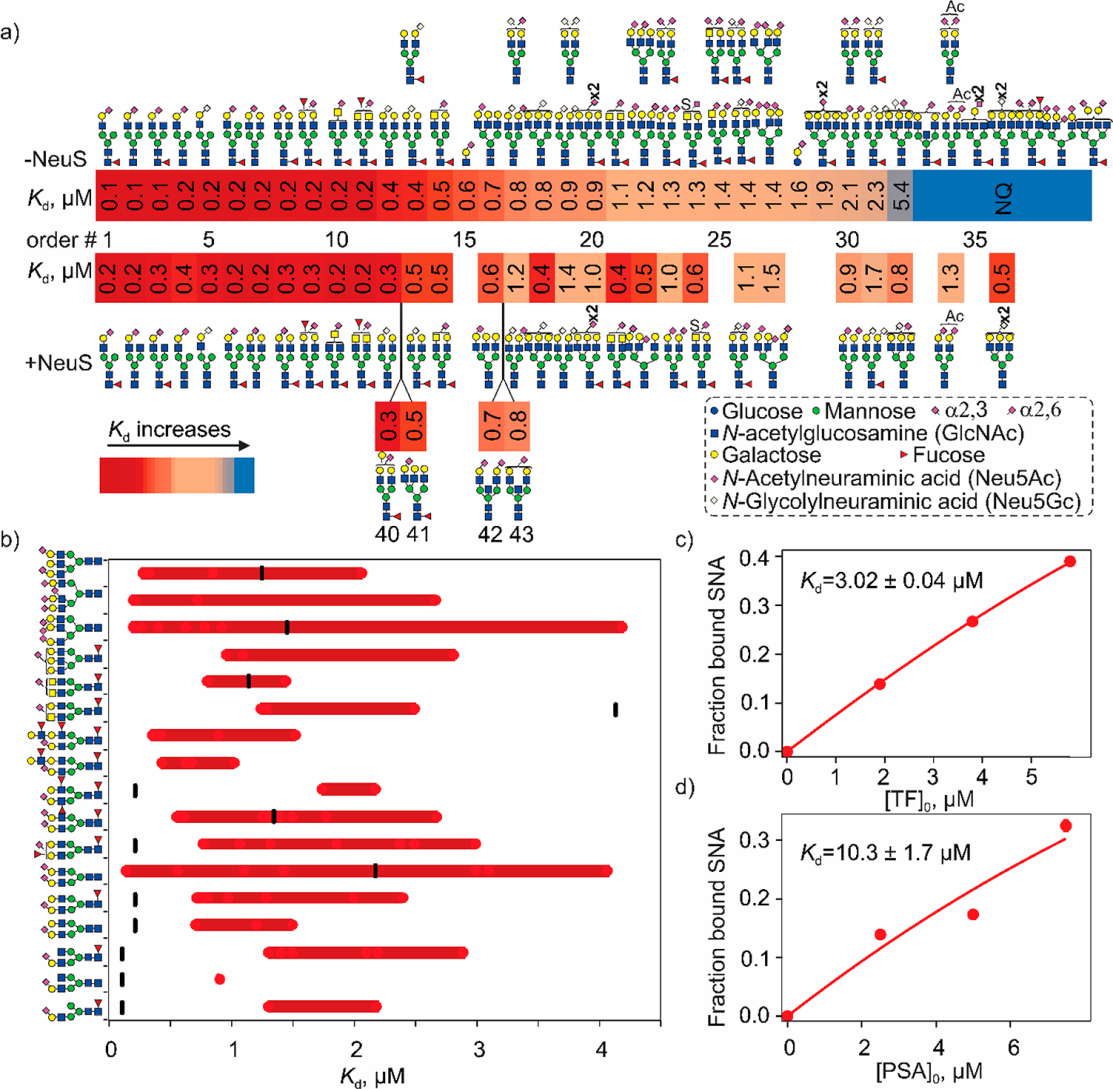
Glycan affinity rankings measured for natural *N*-glycan and glycopeptide libraries screened against SNA using COIN-CaR-nMS. (a) Ranking of highest affinity *N*-glycan ligands detected. When glycans exist as several isoforms, the most abundant one is shown; if several forms are equally abundant, all structures are shown or sialic acid linkage is not specified. The glycan structures from untreated and treated (with neuraminidase) libraries are denoted as −NeuS and +NeuS, respectively. Order number (#) indicates the ranking order. NQ indicates glycan ligand detected but not quantified due to low relative abundance. (b) Range (indicated as red bars) of affinities measured for glycopeptide ligands identified by COIN-CaR-nMS screening. Individual *K*_d_ corresponding to different peptide compositions (red circles) and the *K*_d_ for the free glycan (black dash) are also shown. (c, d) Concentration-dependent fraction of SNA bound to (c) human transferrin (TF) and (d) prostate cancer antigen (PSA) measured by SLOMO-nMS (SNA 10 μM, PSA 2.5–7.5 μM in *Solution 1*, 20 μM in *Solution 2*, TF 2–6 μM in *Solution 1*, 20 μM in *Solution 2*). Solid line is the best fit of eq 4a to the data.

**Figure 5:**
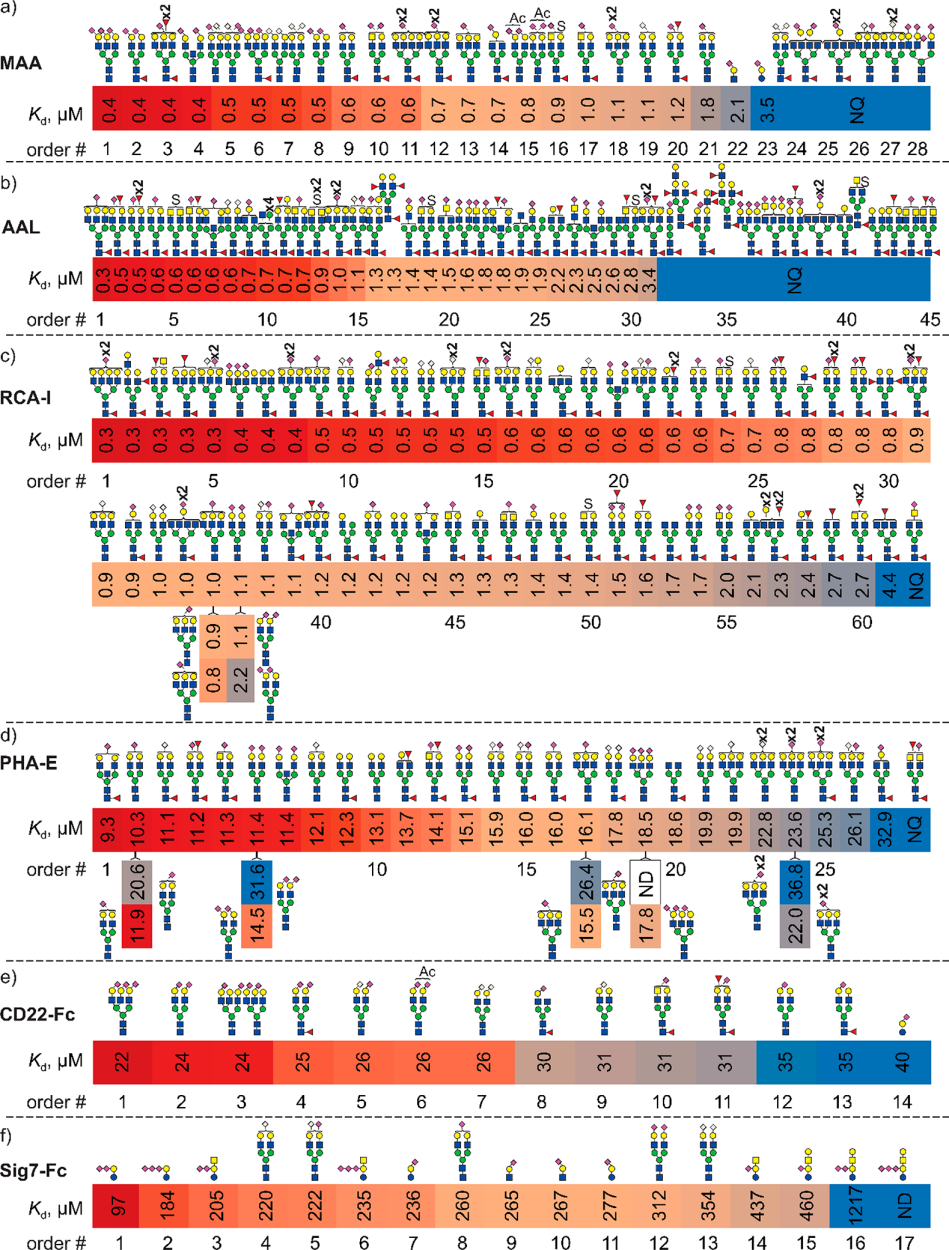
*N*-Glycan affinity rankings measured by COIN-CaR-nMS for plant, fungal, and human GBPs. Highest affinity *N*-glycan ligands measured for (a) MAA, (b) AAL, (c) RCA (RCA-I/RCA_120_), (d) PHA-E, (e) CD22-Fc, and (f) Sig7-Fc are shown together with affinity data measured for select purified glycans by COIN-CaR-nMS. Order number (#) indicates the ranking order. NQ indicates glycan ligand detected but not quantified due to low relative abundance.

While the glycan specificities of lectins commonly used in lectin arrays^37^ and biotechnology applications have been extensively investigated,^38^ there is a dearth of quantative affinity data. Moreover, glycan specificities have been predominantly established using surface-based assays, with few in-solution binding data available for comparison. With these considerations in mind, COIN-CaR-nMS was used to screen the *N*-glycan and glycopeptide libraries against plant (SNA, MAA, RCAI, and PHA-E) and fungal (AAL) lectins which, together, recognize a variety of glycan structures and with a range of affinities. Although COIN-CaR-nMS can, in principle, be performed by pooling the libraries, the present measurements were performed on individual libraries, for which the glycan structures were annotated by HILIC-FLD-MS analysis. The highest affinity *N*-glycan ligands of each lectin and their corresponding *K*_d_ (average values from different experiments, different dilution factors, and different libraries) are summarized in Figures 4a and 5, respectively. Where indicated, *K*_d_ were also measured for purified oligosaccharide ligands to provide additional context to the reported affinity ranking.

#### SNA

According to the COIN-CaR-nMS data (Figure 4a, Table S12), SNA, which is one of the most widely used lectin probes for glycans containing α2–6-linked sialic acid,^38−40^ has a preference for monosialylated (Neu5Ac or Neu5Gc) hybrid- and biantennarary complex *N*-glycans, with the sialic acid on the α3-branch, which is consistent with results obtained by glycan microarray screening.^41,42^ The *K*_d_ of the top ligands are in the 0.1–0.4 μM range. Disialylation, the presence of Man and GlcNAc residues on the α6-branch, and increased branching weaken binding. To our knowledge, reduction in binding resulting from sialylation (regardless of linkage) of the α6-branch has not been previously reported. This finding is supported by *K*_d_ values measured by ITC. In line with the COIN-CaR-nMS data, SNA bound more tightly to α2–6-linked sialic acid on both free (Figure S8) and protein-linked *N*-glycans from transferrin (Figure S9) when the samples were treated with NeuC, which predominantly removes α2–3-linked sialic acid confirming that monosialyated *N*-glycans are more tightly bound than the corresponding disialylated *N*-glycans.

The top *N*-glycan ligands exhibit somewhat stronger binding than **G23** (6SLN; 0.6 μM), pointing to the favorable effect of the underlying LacNAc and Man (and possibly GlcNAc) residues. That **G21** (6SL) is 3-fold weaker ligand than **G23** confirms that the underlying LacNAc structure enhances binding relative to the Lac disaccharide.^38^ The screening results obtained with the libraries treated with α2–3 sialic acid-specific neuraminidase NeuS (Figure 4a) allow for some refinement of the affinity rankings by reducing the number of isomeric species and increasing the concentrations of some of the library components. For example, a number of high-affinity ligands (e.g., structures corresponding to order numbers (#) 40–43) were not captured in the original screen but detected in the NeuS-treated libraries.

Application of COIN-CaR-nMS to the glycopeptides libraries reveals the highly variable effects of a single (N) or multiple amino acid residues on the *N*-glycan specificity (Figure 4b). To our knowledge, such modulating effects have not been previously quantified. For the monosialylated mono- and biantennary *N*-glycan ligands, the presence of amino acid/peptide tends to weaken binding, in many instances significantly. This effect is presumably due to steric clashes with SNA that are absent in the free glycans. For some structures, however, the presence of the peptide can not only decrease but also increase binding, particularly in the case of tri- and tetra-antennary structures. Enhanced binding could arise from either favorable peptide interaction with SNA or favorable (for binding) restriction of glycan conformational space. To provide additional context to the glycopeptide data, the affinity of SNA for two glycoproteins, a PSA standard possessing predominantly α2–6-disialylated biantennary *N*-glycans and TF, which also consists predominantly of α2–6-disialylated biantennary *N*-glycans, was measured using SLOMO. The SNA affinities were found to be 3 μM (TF) and 10 μM (PSA), respectively (Figure 4c,d). That the SNA affinity for TF is similar to that of the free glycan (1.0 μM) suggests little effect of the underlying protein, while for PSA, the underlying protein introduces a 5-fold reduction in affinity, presumably due to steric effects. It is also notable that some of the glycan structures associated with glycopeptide ligands detected were not identified from screening of the *N*-glycan libraries (presumably due to their low abundance in the *N*-glycan libraries). This finding indicates that there is value in screening both *N*-glycan and corresponding glycopeptide libraries. However, interpretation of the data in the context of intrinsic glycan affinity is not straightforward. By extrapolation, these findings raise the possibility that screening glycopeptide libraries containing O-linked glycans may not provide a reliable measure of GBP specificities for free O-linked glycans.

#### MAA

MAA is reported to bind preferentially to 3-*O* sulfated Gal on LacNAc, with terminal α2–3-linked sialic acid on type 2 LacNAc motif also recognized.^38^ The results of COIN-CaR-nMS screening of *N*-glycan libraries reveal that MAA has a preference for mono- and disialylated (Neu5Ac or Neu5Gc) bi- and triantennary *N*-glycans, with affinities ranging from 0.4 to 1.8 μM (Figure 5a, Table S13). Notably, the top hits exhibit stronger (5-fold) binding than **G25** (3SLN), indicating that the underlying Man residues contribute to affinity. It is also found that **G23** (3SL) binding is only moderately weaker (2-fold) than **G25**.

#### AAL

This fungal lectin is reported to recognize α-linked Fuc with a relaxed specificity.^43^ The COIN-CaR-nMS screening results (Figure 5b, Table S14) show that the nature of the *N*-glycan structure modulates affinity, with monosialyted bi- and triantennary *N*-glycans exhibiting the strongest binding, with *K*_d_ of 0.3 to 0.7 μM.

#### RCA-I

The screening data for RCA-I, a lectin that recognizes LacNAc as the main determinant and is frequently used to detect terminal Gal,^38^ reveals high-affinity binding to a large subset of the *N*-glycan library (Figure 5c, Table S15). The top hits are tri- and tetrantennary *N*-glycans, with affinities in the ≥0.3 μM range, a finding consistent with the results of a previous study that suggest branching increases affinity.^44^ It is also notable that several nongalactosylated ligands, possessing terminal GlcNAc, were identified (#53, 58, and 61). These findings, which are at odds with previous screening results,^43^ suggest that RCA-I recognizes GlcNAc, albeit with a *K*_d_ that is approximately 10-fold weaker than for LacNAc. The *K*_d_ (1.7 ± 0.8 μM) obtained by ITC for RCA-I binding to the GlcNAc terminated glycan (**G34**) matches the value obtained by COIN-CaR-nMS for glycan #53 (1.7 ± 0.1 μM), further demonstrating the reliability of the COIN-CaR-nMS assay (Figure 5c). It was reported that RCA-I binding is inhibited by substitutions at the 3 position on Gal but not the 6 position.^43^ Other studies, however, showed that the interaction is partially reduced by both α2–3- and α2–6-linked sialic acid as well as modification on the neighboring GlcNAc residue.^38,44^ To clarify the influence of sialic acid linkage on binding, COIN-CaR-nMS was performed on all α2–3-linked or α2–6-linked *N*-glycan libraries. The results reveal that α2–6-Neu5Ac does not significantly affect binding, while α2,3-Neu5Ac weakens but does not fully abolish binding (#37, Figure 5c), consistent with earlier findings^38,44^

#### PHA-E

It was previously reported that PHA-E exhibits a high specificity for bisected or β-1,6-branched *N*-glycans;^45^ core fucosylation and α2–3-sialylation are well-tolerated, but α2–6-sialylation inhibits binding.^43^ However, the COIN-CaR-nMS results show that PHA-E prefers both bisected and biantennary complex type *N*-glycans, with similar (10–40 μM) *K*_d_ (Figure 5d, Table S16).^46^ In addition, glycans without Gal or with only 1 Gal are also found (#20 and #27) to weakly interact with PHA-E. The affinity is not affected by the presence of α2–3-Neu5Ac; substitution with α2–6-Neu5Ac weakens (∼2-fold) binding, in line with previous findings.^43^ The present results also reveal that PHA-E prefers biatennary to triantennary *N*-glycans, indicating that the lectin is more sensitive to branching than previously reported.^43^

### Human Immune Cell Lectins

The sialic-acid-binding immunoglobulin-like lectins (Siglecs) are a family of cell surface proteins that recognize sialic acid and regulate the innate and adaptive immune systems through glycan binding.^47^ Despite their importance to human health, the glycan binding properties of Siglecs have not been comprehensively established, and relatively few absolute affinities have been reported.^24^ Low affinity and poor solubility of recombinant constructs represent challenges to mapping the glycan specifities of Siglecs.^24^ To illustrate the power of COIN-CaR-nMS screening to uncover Siglec ligands and elucidate structural preferences, the assay was applied to Fc fusions of human Siglec-2 (CD22) and Siglec-7.

#### CD22-Fc

The glycan binding properties of CD22 are the most thoroughly investigated of the human Siglecs. CD22 recognizes α2–6-linked sialic acids,^24^ and, according to the COIN-CaR-nMS screening results for the natural (untreated) and NeuS treated libraries, the top ligands in the libraries are α2–6-disialylated bi- and triantennary *N*-glycans, with a *K*_d_ of ∼25 μM. Monosialylated biantennary *N*-glycans also bind, with a *K*_d_ (30–35 μM) similar to that of 6SL (40 μM) (Figure 5e, Table S17). Preference for disialylated bi- and triantennary *N*-glycans, over monosialylated structures, is consistent with the findings of Paulson and co-workers.^48^ According to the COIN-CaR-nMS data, CD22-Fc binds Neu5Ac and Neu5Gc with indistinguishable *K*_d_, consistent with previous findings.^49^

#### Sig7-Fc

Currently, the functional ligands of Siglec-7 are not well-established. From glycan microarray data, Siglec-7 has a preference α2–8-linked and branched α2–6-linked sialic acid as in GD3 (NeuAcα2,8NeuAcα2,3Galβ1,4Glc) and LSTb (Galβ1,3[NeuAcα2,6]GlcNAcβ1,3Galβ1,4Glc).^50,51^ However, the results of cell-based studies suggest that *O*-glycans with α2–3-linked sialic acid and mucin glycoproteins are ligands.^52−54^ COIN-CaR-nMS screening performed on the *N*-glycan libraries identified few ligands (Figure 5f, Table S18). Nevertheless, these limited data reveal a preference for nonfucosylated mono- and disialylated (Neu5Ac or Neu5Gc) biantennary *N*-glycans, with affinities of 0.2–0.4 mM. The trisaccharides 6SL and 3SL as well as the oligosaccharides of the gangliosides GD3, GT3, GM2, GD2, GT2, GM1, GD1b, and GT1c were tested, and GD3 was found to exhibit the strongest binding (0.1 mM), followed by GT3, GD2, and GT2. Together these results suggest a slight preference for α2–8-linked Neu5Ac. Notably, the *K*_d_ for GD3 oligosaccharide (97 μM) and 6SL (236 μM) are in excellent agreement with the values obtained by ITC (98 μM and 240 μM, respectively); there is poorer agreement for 3SL, though the values agree within a factor of 3 (277 μM (COIN-CaR-nMS), 680 μM (ITC)).^55^ Interestingly, GD1b and GT1c, which also possess α2–8-Neu5Ac, exhibit weak or no binding. *O*-Glycopeptide libraries (contain both *O*- and *N*-glycopeptides) produced from BF and RBD were also screened. Only Neu5Ac-LacNAc-type *O*-glycopeptides were detected (Figure S8), with *K*_d_ in the 0.2–0.3 mM range. Together, these findings suggest that gangliosides may serve as natural ligands for Siglec-7.

### SARS-CoV-2 RBD

SARS-CoV-2 relies on a combination of ACE2 and glycans to bind and infect tissues. The RBD contains the portion of the spike (S) protein that a recognizes ACE2, the primary receptor (attachment factor) exploited by the virus for cell entry.^56^ The RBD also binds heparin sulfate, human blood group glycans, and sialoglycans, including gangliosides and acidic *N*-glycans; both heparin sulfates and acidic glycoplipids have been shown to facilitate viral entry.^57−60^ However, in recent saturation-transfer difference nuclear magnetic resonance (STD-NMR) studies, it was concluded that RBD does not bind 3SL and that the sialic acid binding site is located not on the RBD but on the *N*-terminal domain (NTD) of the S protein.^61,62^ In light of these divergent findings, we sought to more comprehensively profile the sialoglycan binding properties of SARS-CoV-2 RBD.

**Figure 6:**
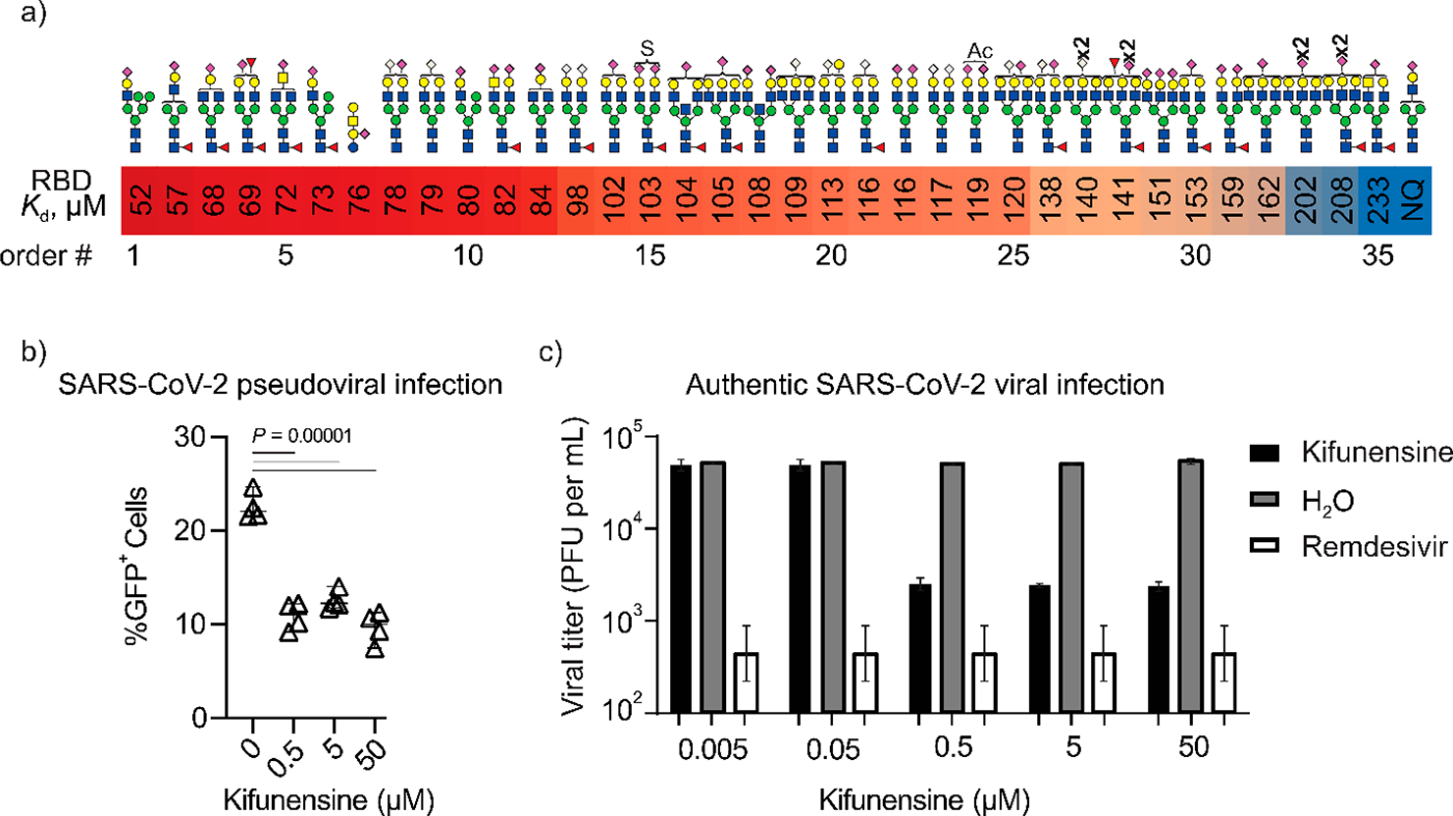
Recognition of *N*-glycans by SARS-CoV-2 RBD. (a) Highest-affinity *N*-glycan ligands measured by COIN-CaR-nMS. Order number (#) indicates the ranking order. NQ indicates glycan ligand detected but not quantified due to low relative abundance. (b) ACE2^+^ HEK293 cells were treated with kifunensin prior to testing infectivity with a GFP-encoding SARS-CoV-2 pseudotyped virus. %GFP^+^ cells were quantified by flow cytometry. Results are representative of four independent experiments. (c) Infection of ACE2^+^ HEK293 cells treated with kifunensin (or H_2_O or remdesivir as controls) with an early clinical isolate of SARS-CoV-2 at 0.1 multiplicity of infection (MOI). Shown is the average viral titers (plaque-forming units (PFUs)) obtained from four biological replicates. Error bars represent 95% CI.

The COIN-CaR-nMS screening data reveal several important findings (Figure 6a, Table S19). First, only sialylated *N*-glycans were identified as ligands; no neutral glycans were detected. These results confirm that SARS-CoV-2 RBD recognizes sialoglycans, including sialylated *N*-glycans (both α2–3- and α2–6-linked).^57^ The highest-affinity ligands are monosialylated hybrid and biantennary *N*-glycans, with affinities (50–75 μM) similar to that of the GM1 pentasaccharide, which was the highest-affinity ligand detected in recent CaR-ESI-MS screening of defined glycan libraries.^57^ Both Neu5Ac and Neu5Gc (bound to either Gal or GalNAc) were recognized with similar affinity (#9, 14, 22, and 23). Increased sialylation and branching led to a slight reduction in binding.

That RBD recognizes both acidic glycolipids and *N*-glycans with similar affinities raises important questions about the functional roles played by *N*-glycans in the infection process. Depletion of glycolipids from the cell surface was shown to attenuate SARS-CoV-2 infection of various ACE2-expressing cells.^57^ Lebrilla and co-workers recently reported an increase binding of RBD to cells treated with kifunensine (which promotes expression of oligomannose *N*-glycans due to blockade in *N*-glycan maturation).^63^ Based on this finding and the observation that RBD binding to host cells is reduced upon incubation with oligomannose glycans, it was concluded that cell surface oligomannose glycans increase adherence of RBD.^63^ However, in the current study, as well as previous screening studies, oligomannose *N*-glycans are not detected as RBD ligands.^57^ To gain further insight into the host cell *N*-glycans on SARS-CoV-2 infection, we performed pseudotyped lentivirus infection of ACE2-expressing HEK293T cells. Infection decreased significantly in cells treated with kifunensine (Figure 6b). We next performed the authentic viral infection of hCoV-19/Canada/ON-VIDO-01/2020 (VIDO) strain with ACE2^+^ HEK293 cells treated with kifunensine at different concentrations. Consistent with the results of the pseudotyped lentivirus infection experiments, we observed that kifunensine treatment resulted in a concentration-dependent decrease in infection (Figure 6c).

Together, these data give new and important insights into the role of host glycans in SARS-CoV-2 cell binding and entry. First, they establish that acidic *N*-glycans are ligands of RBD and that monosialylated hybrid and biantennary *N*-glycans have affinities similar to that of the GM1 pentasaccharide. According to the pseudotyped lentivirus and authentic virus infection data, acidic host *N*-glycans may serve as viral attachment factors. Contrary to previously reported findings, oligomannose *N*-glycans are found not to be RBD ligands.^63^ Finally, the screening data provide irrefutable evidence that SARS-CoV-2 RBD recognizes sialoglycans. That these interactions are not detectable by STD-NMR highlights the enviable sensitivity and analytical advantages of COIN-CaR-nMS for comprehensive mapping of the glycan binding properties of the GBPs of human viruses.

## Conclusions

Interactions between glycans and GBPs are essential for many critical physiological and pathophysiological processes. However, the natural ligands of most GBPs, and their corresponding affinities, are not well-established. Native MS-based SG has emerged as a powerful tool for the discovery of the glycan ligands of GBPs. However, as the glycan concentrations in natural libraries are unknown, affinities cannot be directly measured. In the present work, we introduce COIN-nMS and demonstrate how it enables quantitative screening of natural glycans at unknown concentrations. The assay exploits slow mixing of solutions inside a nanoESI emitter to achieve a continuous change of glycan concentration at the end of the emitter. At early mixing times, and following an initial transient, the glycan concentration flux at the tip of the emitter is approximately linear. From the changes in the relative abundances of GBP–glycan complexes and the glycan concentration flux, the *K*_d_ of all detected GBP–glycan interactions can be determined simultaneously. The reliability of COIN-nMS is demonstrated by affinity measurements performed on a series of purified glycan ligands (individually and as mixtures) of immune lectins with known *K*_d_. Notably, the affinities derived from COIN-nMS agree within a factor of 2 with literature or values determined directly by nMS (at known glycan concentration).

Because direct detection and quantification of glycan interactions with glycosylated GBPs by nMS can be challenging, we demonstrated the implementation of COIN-nMS using the CaR-nMS assay, wherein glycan ligands are detected following their release from the GBP in the gas phase. Application of COIN-CaR-nMS to natural *N*-glycan libraries and a series of lectins demonstrates the tremendous power of the assay for establishing the “fine” glycan specificities of GBPs. Not only are the highest-affinity ligands present in the libraries readily identified, but their affinities are precisely measured. This enables the glycan specificities of GBPs to be established with much greater confidence than is currently possible based on surface-based screening methods. That COIN-CaR-nMS identified ligands of the GBPs that went undetected with other assays further highlights the power of the method for establishing the glycan interactome of GBPs. To extend the potential of COIN-CaR-nMS, future efforts will be directed at expanding the glycan libraries available for screening, including the sources and classes of glycans (e.g., *O*-glycans), performing detailed annotation of the glycan structures contained within the libraries, which will enable glycan ligand specificities to be more precisely established, and demonstrating the feasibility of applying the assay to large (MDa) GBPs and their complexes.

Finally, it is important to emphasize that, while COIN-nMS and COIN-CaR-nMS were conceived for quantitative SG screening, they are easily adapted for libraries of other classes of compounds, including peptides and metabolites. Indeed, the application of COIN-CaR-nMS to natural glycopeptide libraries revealed that the strength of binding to a GBP can be highly modulated by the nature of the peptide. These results, therefore, caution against using glycopeptide library screening to establish the binding specificities of GBPs for free *N*- and *O*-linked glycans.

## Supporting Information Available

Additional details about proteins and reagents; data analysis procedures and related equations; representative mass spectra and COIN-CaR-nMS data; summary of affinities for model systems (PDF)
Mathematical model of the concentration changes in nanoESI emitters due to slow solution mixing (PDF)

## References

[ref1] Varki A. Biological Roles of Glycans. Glycobiology 2017, 27, 3–49. 10.1093/glycob/cww086. 27558841 PMC5884436

[ref2] van Kooyk Y.; Rabinovich G. A. Protein-Glycan Interactions in the Control of Innate and Adaptive Immune Responses. Nat. Immunol. 2008, 9, 593–601. 10.1038/ni.f.203. 18490910

[ref3] Flynn R. A.; Pedram K.; Malaker S. A.; Batista P. J.; Smith B. A. H.; Johnson A. G.; George B. M.; Majzoub K.; Villalta P. W.; Carette J. E.; Bertozzi C. R. Small RNAs Are Modified with N-Glycans and Displayed on the Surface of Living Cells. Cell 2021, 184, 3109–3124e22. 10.1016/j.cell.2021.04.023. 34004145 PMC9097497

[ref4] Hu M.; Lan Y.; Lu A.; Ma X.; Zhang L. Chapter One - Glycan-Based Biomarkers for Diagnosis of Cancers and Other Diseases: Past, Present, and Future. Prog. Mol. Biol. Transl. Sci. 2019, 162, 1–24. 10.1016/bs.pmbts.2018.12.002. 30905444

[ref5] Pinho S. S.; Reis C. A. Glycosylation in Cancer: Mechanisms and Clinical Implications. Nat. Rev. Cancer 2015, 15, 540–555. 10.1038/nrc3982. 26289314

[ref6] Adamczyk B.; Tharmalingam T.; Rudd P. M. Glycans as Cancer Biomarkers. Biochim. Biophys. Acta (BBA) - General Subjects 2012, 1820, 1347–1353. 10.1016/j.bbagen.2011.12.001. 22178561

[ref7] Haukedal H.; Freude K. K. Implications of Glycosylation in Alzheimer’s Disease. Front. Neurosci. 2021, 14, 625348 10.3389/fnins.2020.625348. 33519371 PMC7838500

[ref8] Chen S.; Qin R.; Mahal L. K. Sweet systems: technologies for glycomic analysis and their integration into systems biology. Crit. Rev. Biochem. Mol. Biol. 2021, 56, 301–320. 10.1080/10409238.2021.1908953. 33820453

[ref9] Cummings R. D.; Etzler M.; Hahn M. G.; Darvill A.; Godula K.; Woods R. J.; Mahal L. K. Glycan-Recognizing Probes as Tools. In Essentials of Glycobiology*,* 4th ed.; Varki A.; Cummings R. D.; Esko J. D.; Stanley P.; Hart G. W.; Aebi M.; Mohnen D.; Kinoshita T.; Packer N. H.; Prestegard J. H.; Schnaar R. L.; Seeberger P. H., Eds.; Cold Spring Harbor Laboratory Press: Cold Spring Harbor (NY), 2022; Chapter 48.

[ref10] Cummings R. D. The Repertoire of Glycan Determinants in the Human Glycome. Mol. Biosyst. 2009, 5, 1087–1104. 10.1039/b907931a. 19756298

[ref11] Collins B. E.; Paulson J. C. Cell Surface Biology Mediated by Low Affinity Multivalent Protein–Glycan Interactions. Curr. Opin. Chem. Biol. 2004, 8, 617–625. 10.1016/j.cbpa.2004.10.004. 15556405

[ref12] Lis H.; Sharon N. Lectins: Carbohydrate-Specific Proteins That Mediate Cellular Recognition. Chem. Rev. 1998, 98, 637–674. 10.1021/cr940413g. 11848911

[ref13] Song X.; Lasanajak Y.; Xia B.; Heimburg-Molinaro J.; Rhea J. M.; Ju H.; Zhao C.; Molinaro R. J.; Cummings R. D.; Smith D. F. Shotgun Glycomics: A Microarray Strategy for Functional Glycomics. Nat. Methods 2011, 8, 85–90. 10.1038/nmeth.1540. 21131969 PMC3074519

[ref14] Yu Y.; Mishra S.; Song X.; Lasanajak Y.; Bradley K. C.; Tappert M. M.; Air G. M.; Steinhauer D. A.; Halder S.; Cotmore S.; Tattersall P.; Agbandje-McKenna M.; Cummings R. D.; Smith D. F. Functional Glycomic Analysis of Human Milk Glycans Reveals the Presence of Virus Receptors and Embryonic Stem Cell Biomarkers. J. Biol. Chem. 2012, 287, 44784–44799. 10.1074/jbc.M112.425819. 23115247 PMC3531791

[ref15] Liau B.; Tan B.; Teo G.; Zhang P.; Choo A.; Rudd P. M. Shotgun Glycomics Identifies Tumor-Associated Glycan Ligands Bound by an Ovarian Carcinoma-Specific Monoclonal Antibody. Sci. Rep. 2017, 7, 14489 10.1038/s41598-017-15123-z. 29101385 PMC5670200

[ref16] Byrd-Leotis L.; Liu R.; Bradley K. C.; Lasanajak Y.; Cummings S. F.; Song X.; Heimburg-Molinaro J.; Galloway S. E.; Culhane M. R.; Smith D. F.; Steinhauer D. A.; Cummings R. D. Shotgun Glycomics of Pig Lung Identifies Natural Endogenous Receptors for Influenza Viruses. Proc. Natl. Acad. Sci. U. S. A. 2014, 111, E2241–E2250. 10.1073/pnas.1323162111. 24843157 PMC4050609

[ref17] Grant O. C.; Smith H. M. K.; Firsova D.; Fadda E.; Woods R. J. Presentation, Presentation, Presentation! Molecular-Level Insight into Linker Effects on Glycan Array Screening Data. Glycobiology 2014, 24, 17–25. 10.1093/glycob/cwt083. 24056723 PMC3854501

[ref18] Smith D. F.; Cummings R. D.; Song X. History and Future of Shotgun Glycomics. Biochem. Soc. Trans. 2019, 47, 1–11. 10.1042/BST20170487. 30626702

[ref19] Kilcoyne M.; Gerlach J. Q.; Kane M.; Joshi L. Surface Chemistry and Linker Effects on Lectin–Carbohydrate Recognition for Glycan Microarrays. Anal. Methods 2012, 4, 2721–2728. 10.1039/c2ay25532d.

[ref20] Bui D. T.; Kitova E. N.; Mahal L. K.; Klassen J. S. Mass Spectrometry-Based Shotgun Glycomics for Discovery of Natural Ligands of Glycan-Binding Proteins. Curr. Opin. Struct. Biol. 2022, 77, 102448 10.1016/j.sbi.2022.102448. 36088799

[ref21] Park H.; Jung J.; Rodrigues E.; Kitova E. N.; Macauley M. S.; Klassen J. S. Mass Spectrometry-Based Shotgun Glycomics for Discovery of Natural Ligands of Glycan-Binding Proteins. Anal. Chem. 2020, 92, 14012–14020. 10.1021/acs.analchem.0c02931. 32936606

[ref22] Bui D. T.; Jung J.; Kitova E. N.; Li Z.; Willows S. D.; Boddington M. E.; Kitov P. I.; Mason A. L.; Capicciotti C. J.; Mahal L. K.; Macauley M. S.; Klassen J. S. Mass Spectrometry-Based Shotgun Glycomics Using Labeled Glycan Libraries. Anal. Chem. 2022, 94, 4997–5005. 10.1021/acs.analchem.1c04779. 35302744

[ref23] Bui D. T.; Li Z.; Kitov P. I.; Han L.; Kitova E. N.; Fortier M.; Fuselier C.; Granger Joly de Boissel P.; Chatenet D.; Doucet N.; Tompkins S. M.; St-Pierre Y.; Mahal L. K.; Klassen J. S. Quantifying Biomolecular Interactions Using Slow Mixing Mode (SLOMO) Nanoflow ESI-MS. ACS Cent. Sci. 2022, 8, 963–974. 10.1021/acscentsci.2c00215. 35912341 PMC9335916

[ref24] Rodrigues E.; Jung J.; Park H.; Loo C.; Soukhtehzari S.; Kitova E. N.; Mozaneh F.; Daskhan G.; Schmidt E. N.; Aghanya V.; Sarkar S.; Streith L.; St Laurent C. D.; Nguyen L.; Julien J.-P.; West L. J.; Williams K. C.; Klassen J. S.; Macauley M. S. A Versatile Soluble Siglec Scaffold for Sensitive and Quantitative Detection of Glycan Ligands. Nat. Commun. 2020, 11, 5091 10.1038/s41467-020-18907-6. 33037195 PMC7547722

[ref25] Mitchell D. A.; Fadden A. J.; Drickamer K. A Novel Mechanism of Carbohydrate Recognition by the C-Type Lectins DC-SIGN and DC-SIGNR. J. Biol. Chem. 2001, 276, 28939–28945. 10.1074/jbc.M104565200. 11384997

[ref26] Báez Bolivar E. G.; Bui D. T.; Kitova E. N.; Han L.; Zheng R. B.; Luber E. J.; Sayed S. Y.; Mahal L. K.; Klassen J. S. Submicron Emitters Enable Reliable Quantification of Weak Protein–Glycan Interactions by ESI-MS. Anal. Chem. 2021, 93, 4231–4239. 10.1021/acs.analchem.0c05003. 33630563

[ref27] Gut H.; King S. J.; Walsh M. A. Structural and Functional Studies of Streptococcus Pneumoniae Neuraminidase B: An Intramolecular Trans-Sialidase. FEBS Lett. 2008, 582, 3348–3352. 10.1016/j.febslet.2008.08.026. 18775704

[ref28] Lin H.; Kitova E. N.; Klassen J. S. Quantifying Protein–Ligand Interactions by Direct Electrospray Ionization-MS Analysis: Evidence of Nonuniform Response Factors Induced by High Molecular Weight Molecules and Complexes. Anal. Chem. 2013, 85, 8919–8922. 10.1021/ac401936x. 24044528

[ref29] Shams-Ud-Doha K.; Kitova E. N.; Kitov P. I.; St-Pierre Y.; Klassen J. S. Human Milk Oligosaccharide Specificities of Human Galectins. Comparison of Electrospray Ionization Mass Spectrometry and Glycan Microarray Screening Results. Anal. Chem. 2017, 89, 4914–4921. 10.1021/acs.analchem.6b05169. 28345865

[ref30] Bashkatov A. N.; Genina E. A.; Sinichkin Y. P.; Kochubey V. I.; Lakodina N. A.; Tuchin V. V. Glucose and Mannitol Diffusion in Human Dura Mater. Biophys. J. 2003, 85, 3310–3318. 10.1016/S0006-3495(03)74750-X. 14581232 PMC1303608

[ref31] Park G. Y. Diffusion Coefficient Calculated by Complementary Error Function for the Sublimation Diffusion of Disperse Dye. J. Eng. Fiber Fabr. 2019, 14, 155892501986659 10.1177/1558925019866592.

[ref32] van Liempt E.; Bank C. M. C.; Mehta P.; Garcıa-Vallejo J. J.; Kawar Z. S.; Geyer R.; Alvarez R. A.; Cummings R. D.; Kooyk Y. v.; van Die I. Specificity of DC-SIGN for Mannose- and Fucose-Containing Glycans. FEBS Lett. 2006, 580, 6123–6131. 10.1016/j.febslet.2006.10.009. 17055489

[ref33] Guo Y.; Feinberg H.; Conroy E.; Mitchell D. A.; Alvarez R.; Blixt O.; Taylor M. E.; Weis W. I.; Drickamer K. Structural Basis for Distinct Ligand-Binding and Targeting Properties of the Receptors DC-SIGN and DC-SIGNR. Nat. Struct. Mol. Biol. 2004, 11, 591–598. 10.1038/nsmb784. 15195147

[ref34] Gao C.; Stavenhagen K.; Eckmair B.; McKitrick T. R.; Mehta A. Y.; Matsumoto Y.; McQuillan A. M.; Hanes M. S.; Eris D.; Baker K. J.; Jia N.; Wei M.; Heimburg-Molinaro J.; Ernst B.; Cummings R. D. Differential Recognition of Oligomannose Isomers by Glycan-Binding Proteins Involved in Innate and Adaptive Immunity. Sci. Adv. 2021, 7, eabf6834 10.1126/sciadv.abf6834. 34108208 PMC8189592

[ref35] Noll A. J.; Yu Y.; Lasanajak Y.; Duska-McEwen G.; Buck R. H.; Smith D. F.; Cummings R. D. Human DC-SIGN Binds Specific Human Milk Glycans. Biochem. J. 2016, 473, 1343–1353. 10.1042/BCJ20160046. 26976925 PMC4875834

[ref36] Martínez J. D.; Valverde P.; Delgado S.; Romanò C.; Linclau B.; Reichardt N. C.; Oscarson S.; Ardá A.; Jiménez-Barbero J.; Cañada F. J. Unraveling Sugar Binding Modes to DC-SIGN by Employing Fluorinated Carbohydrates. Molecules 2019, 24, 2337 10.3390/molecules24122337. 31242623 PMC6631030

[ref37] Pilobello K. T.; Slawek D. E.; Mahal L. K. A ratiometric lectin microarray approach to analysis of the dynamic mammalian glycome. Proc. Natl. Acad. Sci. U.S.A. 2007, 104, 11534–11539. 10.1073/pnas.0704954104. 17606908 PMC1913879

[ref38] Bojar D.; Meche L.; Meng G.; Eng W.; Smith D. F.; Cummings R. D.; Mahal L. K. A Useful Guide to Lectin Binding: Machine-Learning Directed Annotation of 57 Unique Lectin Specificities. ACS Chem. Biol. 2022, 17, 2993–3012. 10.1021/acschembio.1c00689. 35084820 PMC9679999

[ref39] Llop E.; Ferrer-Batallé M.; Barrabés S.; Guerrero P. E.; Ramírez M.; Saldova R.; Rudd P. M.; Aleixandre R. N.; Comet J.; de Llorens R.; Peracaula R. Improvement of Prostate Cancer Diagnosis by Detecting PSA Glycosylation-Specific Changes. Theranostics 2016, 6, 1190–1204. 10.7150/thno.15226. 27279911 PMC4893645

[ref40] Wu J.; Xie X.; Nie S.; Buckanovich R. J.; Lubman D. M. Altered Expression of Sialylated Glycoproteins in Ovarian Cancer Sera Using Lectin-Based ELISA Assay and Quantitative Glycoproteomics Analysis. J. Proteome. Res. 2013, 12, 3342–3352. 10.1021/pr400169n. 23731285

[ref41] Smith D. F.; Song X.; Cummings R. D. Use of Glycan Microarrays to Explore Specificity of Glycan-Binding Proteins. Methods Enzymol. 2010, 480, 417–444. 10.1016/S0076-6879(10)80033-3. 20816220

[ref42] Li L.; Guan W.; Zhang G.; Wu Z.; Yu H.; Chen X.; Wang P. G. Microarray Analyses of Closely Related Glycoforms Reveal Different Accessibilities of Glycan Determinants on N-Glycan Branches. Glycobiology 2020, 30, 334–345. 10.1093/glycob/cwz100. 32026940 PMC7175966

[ref43] Gao C.; Hanes M. S.; Byrd-Leotis L. A.; Wei M.; Jia N.; Kardish R. J.; McKitrick T. R.; Steinhauer D. A.; Cummings R. D. Unique Binding Specificities of Proteins toward Isomeric Asparagine-Linked Glycans. Cell Chem. Biol. 2019, 26, 535–547e4. 10.1016/j.chembiol.2019.01.002. 30745240 PMC7597375

[ref44] Itakura Y.; Nakamura-Tsuruta S.; Kominami J.; Sharon N.; Kasai K.; Hirabayashi J. Systematic Comparison of Oligosaccharide Specificity of Ricinus Communis Agglutinin I and Erythrina Lectins: A Search by Frontal Affinity Chromatography. J. Biochem. 2007, 142, 459–469. 10.1093/jb/mvm153. 17652328

[ref45] Kaneda Y.; Whittier R. F.; Yamanaka H.; Carredano E.; Gotoh M.; Sota H.; Hasegawa Y.; Shinohara Y. The High Specificities of Phaseolus Vulgaris Erythro- and Leukoagglutinating Lectins for Bisecting GlcNAc or β1–6-Linked Branch Structures, Respectively, Are Attributable to Loop B. J. Biol. Chem. 2002, 277, 16928–16935. 10.1074/jbc.M112382200. 11864980

[ref46] Nagae M.; Soga K.; Morita-Matsumoto K.; Hanashima S.; Ikeda A.; Yamamoto K.; Yamaguchi Y. Phytohemagglutinin from Phaseolus Vulgaris (PHA-E) Displays a Novel Glycan Recognition Mode Using a Common Legume Lectin Fold. Glycobiology 2014, 24, 368–378. 10.1093/glycob/cwu004. 24436051

[ref47] MacAuley M. S.; Crocker P. R.; Paulson J. C. Siglec-Mediated Regulation of Immune Cell Function in Disease. Nat. Rev. Immunol. 2014, 14, 653–666. 10.1038/nri3737. 25234143 PMC4191907

[ref48] Peng W.; Paulson J. C. CD22 Ligands on a Natural N-Glycan Scaffold Efficiently Deliver Toxins to B-Lymphoma Cells. J. Am. Chem. Soc. 2017, 139, 12450–12458. 10.1021/jacs.7b03208. 28829594 PMC5755971

[ref49] Macauley M. S.; Kawasaki N.; Peng W.; Wang S.-H.; He Y.; Arlian B. M.; McBride R.; Kannagi R.; Khoo K.-H.; Paulson J. C. Unmasking of CD22 Co-Receptor on Germinal Center B-Cells Occurs by Alternative Mechanisms in Mouse and Man. J. Biol. Chem. 2015, 290, 30066–30077. 10.1074/jbc.M115.691337. 26507663 PMC4705971

[ref50] Crocker P. R.; Paulson J. C.; Varki A. Siglecs and Their Roles in the Immune System. Nat. Rev. Immunol. 2007, 7, 255–266. 10.1038/nri2056. 17380156

[ref51] Yamaji T.; Teranishi T.; Alphey M. S.; Crocker P. R.; Hashimoto Y. A Small Region of the Natural Killer Cell Receptor, Siglec-7, Is Responsible for Its Preferred Binding to α2,8-Disialyl and Branched α2,6-Sialyl Residues: a comparison with Siglec-9. J. Biol. Chem. 2002, 277, 6324–6332. 10.1074/jbc.M110146200. 11741958

[ref52] Wisnovsky S.; Möckl L.; Malaker S. A.; Pedram K.; Hess G. T.; Riley N. M.; Gray M. A.; Smith B. A. H.; Bassik M. C.; Moerner W. E.; Bertozzi C. R. Genome-Wide CRISPR Screens Reveal a Specific Ligand for the Glycan-Binding Immune Checkpoint Receptor Siglec-7. Proc. Natl. Acad. Sci. U. S. A. 2021, 118, e2015024118 10.1073/pnas.2015024118. 33495350 PMC7865165

[ref53] Narimatsu Y.; Joshi H. J.; Nason R.; Van Coillie J.; Karlsson R.; Sun L.; Ye Z.; Chen Y.-H.; Schjoldager K. T.; Steentoft C.; Furukawa S.; Bensing B. A.; Sullam P. M.; Thompson A. J.; Paulson J. C.; Büll C.; Adema G. J.; Mandel U.; Hansen L.; Bennett E. P.; Varki A.; Vakhrushev S. Y.; Yang Z.; Clausen H. An Atlas of Human Glycosylation Pathways Enables Display of the Human Glycome by Gene Engineered Cells. Mol. Cell 2019, 75, 394–407e5. 10.1016/j.molcel.2019.05.017. 31227230 PMC6660356

[ref54] Malaker S. A.; Pedram K.; Ferracane M. J.; Bensing B. A.; Krishnan V.; Pett C.; Yu J.; Woods E. C.; Kramer J. R.; Westerlind U.; Dorigo O.; Bertozzi C. R. The Mucin-Selective Protease StcE Enables Molecular and Functional Analysis of Human Cancer-Associated Mucins. Proc. Natl. Acad. Sci. U. S. A. 2019, 116, 7278–7287. 10.1073/pnas.1813020116. 30910957 PMC6462054

[ref55] Movsisyan L. D.; Macauley M. S. Structural Advances of Siglecs: Insight into Synthetic Glycan Ligands for Immunomodulation. Org. Biomol. Chem. 2020, 18, 5784–5797. 10.1039/D0OB01116A. 32756649

[ref56] Lan J.; Ge J.; Yu J.; Shan S.; Zhou H.; Fan S.; Zhang Q.; Shi X.; Wang Q.; Zhang L.; Wang X. Structure of the SARS-CoV-2 Spike Receptor-Binding Domain Bound to the ACE2 Receptor. Nature 2020, 581, 215–220. 10.1038/s41586-020-2180-5. 32225176

[ref57] Nguyen L.; McCord K. A.; Bui D. T.; Bouwman K. M.; Kitova E. N.; Elaish M.; Kumawat D.; Daskhan G. C.; Tomris I.; Han L.; Chopra P.; Yang T.-J.; Willows S. D.; Mason A. L.; Mahal L. K.; Lowary T. L.; West L. J.; Hsu S.-T. D.; Hobman T.; Tompkins S. M.; Boons G.-J.; de Vries R. P.; Macauley M. S.; Klassen J. S. Sialic Acid-Containing Glycolipids Mediate Binding and Viral Entry of SARS-CoV-2. Nat. Chem. Biol. 2022, 18, 81–90. 10.1038/s41589-021-00924-1. 34754101 PMC12434308

[ref58] Clausen T. M.; Sandoval D. R.; Spliid C. B.; Pihl J.; Perrett H. R.; Painter C. D.; Narayanan A.; Majowicz S. A.; Kwong E. M.; McVicar R. N.; Thacker B. E.; Glass C. A.; Yang Z.; Torres J. L.; Golden G. J.; Bartels P. L.; Porell R. N.; Garretson A. F.; Laubach L.; Feldman J.; Yin X.; Pu Y.; Hauser B. M.; Caradonna T. M.; Kellman B. P.; Martino C.; Gordts P. L. S. M.; Chanda S. K.; Schmidt A. G.; Godula K.; Leibel S. L.; Jose J.; Corbett K. D.; Ward A. B.; Carlin A. F.; Esko J. D. SARS-CoV-2 Infection Depends on Cellular Heparan Sulfate and ACE2. Cell 2020, 183, 1043–1057e15. 10.1016/j.cell.2020.09.033. 32970989 PMC7489987

[ref59] Wu S.-C.; Arthur C. M.; Wang J.; Verkerke H.; Josephson C. D.; Kalman D.; Roback J. D.; Cummings R. D.; Stowell S. R. The SARS-CoV-2 Receptor-Binding Domain Preferentially Recognizes Blood Group A. Blood Adv. 2021, 5, 1305–1309. 10.1182/bloodadvances.2020003259. 33656534 PMC7929867

[ref60] Boukhari R.; Breiman A.; Jazat J.; Ruvoën-Clouet N.; Martinez S.; Damais-Cepitelli A.; le Niger C.; Devie-Hubert I.; Penasse F.; Mauriere D.; Sébille V.; Dürrbach A.; le Pendu J. ABO Blood Group Incompatibility Protects Against SARS-CoV-2 Transmission. Front. Microbiol. 2022, 12, 799519 10.3389/fmicb.2021.799519. 35069504 PMC8767008

[ref61] Creutznacher R.; Maass T.; Veselkova B.; Ssebyatika G.; Krey T.; Empting M.; Tautz N.; Frank M.; Kölbel K.; Uetrecht C.; Peters T. NMR Experiments Provide Insights into Ligand-Binding to the SARS-CoV-2 Spike Protein Receptor-Binding Domain. J. Am. Chem. Soc. 2022, 144, 13060–13065. 10.1021/jacs.2c05603. 35830336

[ref62] Buchanan C. J.; Gaunt B.; Harrison P. J.; Yang Y.; Liu J.; Khan A.; Giltrap A. M.; Le Bas A.; Ward P. N.; Gupta K.; Dumoux M.; Tan T. K.; Schimaski L.; Daga S.; Picchiotti N.; Baldassarri M.; Benetti E.; Fallerini C.; Fava F.; Giliberti A.; Koukos P. I.; Davy M. J.; Lakshminarayanan A.; Xue X.; Papadakis G.; Deimel L. P.; Casablancas-Antràs V.; Claridge T. D. W.; Bonvin A. M. J. J.; Sattentau Q. J.; Furini S.; Gori M.; Huo J.; Owens R. J.; Schaffitzel C.; Berger I.; Renieri A.; Naismith J. H.; Baldwin A. J.; Davis B. G. Pathogen-Sugar Interactions Revealed by Universal Saturation Transfer Analysis. Science 2022, 377, 385 10.1126/science.abm3125. 35737812

[ref63] Sheng Y.; Vinjamuri A.; Alvarez M. R. S.; Xie Y.; McGrath M.; Chen S.; Barboza M.; Frieman M.; Lebrilla C. B. Host Cell Glycocalyx Remodeling Reveals SARS-CoV-2 Spike Protein Glycomic Binding Sites. Front. Mol. Biosci. 2022, 9, 799703 10.3389/fmolb.2022.799703. 35372520 PMC8964299

