# Supplementary material for: Absolute Affinities from Quantitative Shotgun Glycomics Using Concentration-Independent (COIN) Native Mass Spectrometry
Source: ACS Cent Sci. 2023 Jun 15;9(7):1374–87. doi: 10.1021/acscentsci.3c00294 (PMC10303200; doi:10.1021/acscentsci.3c00294)
Supplement: Supplementary file 1 — oc3c00294_si_001.pdf [file oc3c00294_si_001.pdf]

Supporting Information File 1 for:

Absolute Affinities from Quantitative Shotgun Glycomics Using Concentration-Independent (COIN) Native Mass Spectrometry

Duong T. Bui,[1] James Favell,[1] Elena N. Kitova,[1] Zhixiong Li,[1] Kelli A. McCord,[1] Edward N. Schmidt[1], Fahima Mozaneh,[1] Mohamed Elaish,[2,3] Amr El-Hawiet,[4] Yves St-Pierre,[5] Tom C. Hobman,[2,6,7] Matthew S. Macauley,[1,6] Lara K. Mahal,[1] Morris R. Flynn,[8] and John S. Klassen[1]*

[1]*Department of Chemistry, University of Alberta, Edmonton, Alberta, Canada T6G 2G2*

[2] *Department of Cell Biology, University of Alberta, Edmonton, AB, Canada T6G 2H7*

[3] *Poultry Diseases Department, Faculty of Veterinary Medicine, Cairo University, Giza, Egypt*

[4]*Department of Pharmacognosy, Faculty of Pharmacy, Alexandria University, Alexandria, Egypt*

[5] *Institut National de la Recherche Scientifique (INRS), INRS-Centre Armand-Frappier Santé Biotechnologie, Laval, QC, Canada H7V 1B7*

[6] *Department of Medical Microbiology and Immunology, University of Alberta, Edmonton, AB, Canada T6G 2E1*

[7]*Li Ka Shing Institute of Virology, University of Alberta, Edmonton, Alberta, Canada. T6G 2E1*

[8] *Department of Mechanical Engineering, Faculty of Engineering, University of Alberta, Edmonton, Alberta, Canada T6G 1H9*

* Corresponding Author:

Department of Chemistry

University of Alberta

Edmonton, AB CANADA T6G 2G2

Email: john.klassen@ualberta.ca

Telephone: (780) 492-3501

https://orcid.org/0000-0002-3389-7112

Table of contents

Materials and methods…………………………………………………………………....S5

Production of *N*-glycan libraries……………………………………………………....S5

Production of glycopeptide libraries…………………………………………………......S5

Remodeling of *N*-glycan libraries……………………………………………………..S5

Preparation of deuterated 5-(acetyl-2,2,2-d3-amino)-(2→3)-O- β -D-galactopyranosyl-(1→4)- β -D-glucopyranose…………………………………………………………………...…S6

Isothermal titration calorimetry…….…………………………………….….….......S7

HILIC-UHPLC analysis……………………………………………………………...….S8

Mass spectrometry…………………………………………………………...……......S9

Calculation of glycan concentration in diffusion experiments in the presence of GBP …………..S9

Assessing the dynamic range of COIN-CaR-nMS…………………………………...S10

Pseudovirus production and transduction and Pharmacological modulation of *N*-glycan type…S10

Authentic SARS-CoV-2 viral infection assays…………………………………….....S10

Tables…………………………………………………………………………......S12

Table S1. Glycan ID, chemical composition, symbol nomenclature (SNFG) and molecular weight (MW) of purified glycans used in the study…………………………………………….….S12

Table S2. Affinities (K_d, μM) measured by nMS and COIN-nMS for GAL-3C binding with **G1** and **G2**, GAL-7 homodimer with **G3**, fCD22 with **G4** and DC-SIGN CRD with **G5**……….......S14

Table S3. Affinities (K_d, μM) measured by nMS and COIN-nMS for GAL-3C binding with mixture of glycans (**G2**, **G5-G13**)…………………………………………………...S15

Table S4. Affinities (K_d, μM) measured by nMS and COIN-nMS for GAL-7 homodimer binding with mixture of glycans (**G2**, **G5-G13**)……………………………………...…….S16

Table S5. Affinities (K_d, μM) measured by nMS and COIN-nMS for DC-SIGN CRD binding with mixture of glycans (**G2**, **G5**, **G10**, **G14-G20**)…………………..……………………………...S17

Table S6 Affinities (K_d, μM) measured by COIN-CaR-nMS for SNA binding with **G21**, MAA with **G22**, CD22-Fc with **G21** and GNA with **G5**…………………………………..……..S18

Table S7 Affinities (K_d, μM) measured by COIN-CaR-nMS for SNA and Sig7-Fc with pairs of glycans at different relative concentrations…………………...…………..……………………S19

Table S8 Annotation of *N*- and *O*- linked glycopeptides derived from pronase digestion of bovine fetuin……………………………………………………………………...............…S20

Table S9 Annotation of *N*-linked glycopeptides derived from pronase digestion of human lactoferrin……………………………………………………………………………….S23

Table S10 Annotation of *N*-linked glycopeptides derived from pronase digestion of bovine AGP……………………………………………………………………………………….S25

Table S11 Annotation of *N*- and *O*-linked glycopeptides derived from pronase digestion of SARS-CoV-2 RBD………………………………………………………………………..S27

Table S12. Affinities of *N*-glycans from the native and NeuS treated natural libraries and 6SL (**G21**) and 6SLN (**G23**) to SNA……………………….…………………………...…..S29

Table S13. Affinities of *N*-glycans from the native and all α2,3 linked natural libraries and 3SL (**G22**) and 3SLN (**G25**) to MAA……………………………………………………..…S31

Table S14. Affinities of *N*-glycans from the native natural libraries to AAL…………………S32

Table S15. Affinities of *N*-glycans from the native natural libraries and α2,6-NeuAc- and α2,3-NeuAc-linked glycans to RCA-I……………………………………....…………….S34

Table S16. Affinities of *N*-glycans from the native natural libraries and α2-6-NeuAc- and α2-3-NeuAc-linked glycans to PHA-E……………………………………………………….S36

Table S17. Affinities of *N*-glycans from the native natural libraries and **G21** to CD22-Fc…...S37

Table S18. Affinities of *N*-glycans from the native natural libraries and purified oligosaccharides to Sig7-Fc………………………………………………………..……………………S38

Table S19. Affinities of *N*-glycans from the native natural libraries and purified oligosaccharides to RBD……………………………………………………...……………………..….S39

Figures……………………..…………………………………………………………..S40

Figure S1. Time-dependent concentration changes of **G23** in the presence of GBP (GAL-3C) and the best fit for the region of data after mixing onset using linear, logarithmic and quadratic models……………………………………………………………………………………..S40

Figure S2. ITC measurements of SNA binding to **G21**………………………..……………S41

Figure S3. ITC measurements of MAA binding to **G23**………..……………………………...S42

Figure S4. Glycan composition of the enzymes treated bovine fetuin (BF)……………..…..…S43

Figure S5. Glycan composition of the enzymes treated human lactoferrin……………………..…S44

Figure S6. Glycan composition of the enzymes treated human bovine AGP (bAGP)………..….S45

Figure S7. Glycan composition of the enzymes treated RBD……………………………..…..…...S46

Figure S8. ITC measurements of SNA binding to *N*-glycans…………………………..…..…...S47

Figure S9. ITC measurements of SNA binding to human transferrin…………..………..…...S48

Figure S10. ITC measurements of RCA-I binding to **G34**……………………………..…..…...S49

Figure S11. Representative CaR-nMS mass spectrum measured for solution of Sig7-Fc and pronase digested RBD and BF…………………………………………………………..…S49

References……………………………………………………..…………………….…S50

Materials and Methods

Production of *N*-glycan libraries. *N*-glycans were produced from glycoproteins using PNGase F (New England Biolab, Massachusetts, United States) digestion using the following protocol. After dialysis in ammonium acetate solution (200 mM, pH 6.8), the proteins were incubated with PNGase F at 37 ^{o}C for 24 h. The proteins were then dialyzed 3 times in water using using Amicon Ultra-0.5 mL centrifugal filters (EMD Millipore, Billerica, MA, USA) with 10 kDa MW cut-off. The flow through containing glycans were collected and lyophilized. The dried glycans were then dissolved in mass spectrometry grade water and stored at -20 ^{o}C until used.

Production of glycopeptide libraries. To produce glycopeptide libraries, glycoproteins were dialyzed in ammonium acetate as described above. After that the glycoproteins were incubated with pronase (Roche Diagnostics GmbH, Mannheim, Germany) 37 ^{o}C for 7 d. The samples were then dialyzed 3 times in water using using Amicon Ultra-0.5 mL centrifugal filters (EMD Millipore, Billerica, MA, USA) with 10 kDa MW cut-off. The flow through containing glycopeptides were collected and lyophilized. The dried glycopeptides were then dissolved in mass spectrometry grade water and stored at -20 ^{o}C until used.

Remodeled *N*-glycan libraries. Libraries with all α2-6-linked Neu5Ac/Neu5Gc. To produce glycan libraries with all α2,6-linked Neu5Ac/Neu5Gc, NeuS (neuraminidase S, New England BioLabs, MA, USA was added to the glycoproteins solution (Ammonium acetate 200 mM, pH 6.8) and incubated for 24 h. Then the proteins were dialyzed in 200 mM ammonium acetate solution using Amicon Ultra-0.5 mL centrifugal filters (EMD Millipore, Billerica, MA, USA) with 10 kDa MW cut-off to remove the hydrolyzed Neu5Ac/Neu5c. The proteins with α2-6-Neu5Ac/Neu5Gc were then treated with PNGaseF and purified as described above. A library with all α2-3-Neu5Ac was produced from asialo bovine fetuin (aBF) by incubating with CMP-Neu5Ac

with ST3Gal4 soluble recombinant form of human sialyltransferases ST3Gal4 (UniProt Q11206, amino acid residues 41-333), expressed in Freestyle 293F cells (Thermo Fisher Scientific) as green fluorescent protein (GFP) fusions in the pGEn2 vector.[S1,S2]

Preparation of deuterated 5-(Acetyl-2,2,2-d$_3$-amino)-(2→3)-O-β-D-galactopyranosyl-(1→4)-β-D-glucopyranos

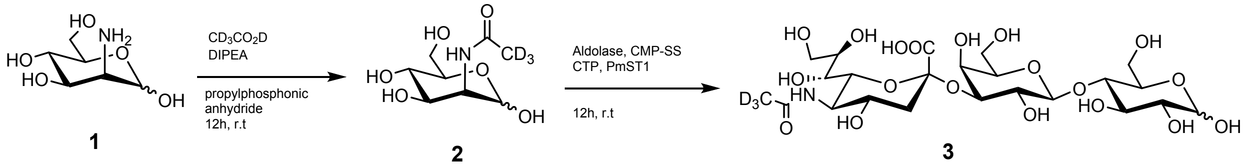

2-(Acetyl-2,2,2-d$_3$-amino)-2-deoxy-α-β-D-glucopyranose 2.

To a solution of mannosamine (1 eq, 20 mg, 0.11 mmol) in pyridine (5 ml), Acetic acid D_4 (2 eq, 13 ul, 0.22 mmol), DIPEA (12 eq, 233 ul, 1.3 mmol) and propylphosphonic anhydride (2 eq, 68 ul, 0.22 mmol) were added, and the mixture was stirred at room temperature for 12 hours and monitored using TLC in i-PrOH:NH_4OH:H_2O (7: 2: 1, by volume). After completion of the reaction, the solvent was evaporated, the crude product was dissolved in water and purified on P2 gel filtration to afford **2** (21 mg, 90 % yield). ^{1}H NMR (500 MHz, D_2O) δ = 5.10 (s, 1H), 5.00 (s, 1H), 4.43 (d, 1H, J = 3.5 Hz), 4.31 (d, 1H, J = 3.5 Hz), 4.14 (dd, 1H, J = 5.0, 10.0 Hz), 3.78 - 3.88 (m, 3H), 3.61 (t, 1H, J = 9.5 Hz), 3.40 (ddd, 1H, J = 2.5, 5.0, 7.5 Hz).

5-(Acetyl-2,2,2-d$_3$-amino)-(2→3)-O-β-D-galactopyranosyl-(1→4)-β-D-glucopyranose 3.

A mixture of **2** (1 eq, 5 mg, 0.022 mmol), lactose (1.2 eq, 5 mg, 0.014 mmol), CTP (1.5 eq, 13 mg, 0.026 mmol) CMP- sialic acid synthetase (NYS05) (0.25 mg/ml), pyruvate (5 eq, 12.3 mg, 0.021 mmol), aldolase (4 mg), PmST1 (ST3) (0.15 mg/ml), and recombinant shrimp alkaline phosphatase (rSAP) (NEB) (1 μL) were dissolved in Tris-HCl (100 mM, pH 8.5) containing $MgCl_2$ (10 mM). Reaction was carried out at 37 °C for 12 and monitored in i-PrOH: NH_4OH:H_2O (5: 2: 1, by volume) as developing solvent. The reaction was stopped by adding 4-fold prechilled 95%

ethanol and centrifuged at 37000 rcf for 15 min. The supernatant was decanted into a round bottom flask and evaporated. The residue was resuspended in water and purified on a P2 column equilibrated in 20% NH4OH giving compound **3** (9.8 mg, 71% yield) from the reaction mixture. H^1 NMR (700 MHz, D_2O) δ = 4.65 (d, 1H, *J* = 8.5 Hz), 4.52 (d, 1H, *J* = 7.7 Hz), 4.11 (d, 1H, *J* = 9.8 Hz), 3.71 - 3.91 (m, 4H), 3.67- 3.74 (m, 6H), 3.62 – 3.66 (m, 5H), 3.55 – 3.61 (m, 3H), 3.26 (t, 1H, *J* = 1.4 Hz), 2.76 (dd, 1H, *J* = 7.7, 9.8 Hz), 1.8 (t, 1H, *J* = 11.9 Hz).

Isothermal Titration Calorimetry (ITC). The ITC measurement was carried out using a Microcal PEAQ ITC (Malvern Panalytical, Worcestershire, United Kingdom). All measurements were performed in aqueous ammonium acetate solutions (200 mM, pH 6.8, 25 °C). First injection was 0.4 μL, 2 μL/injection for 2-19 injections. The reference power was adjusted for each measurement, duration 0.8 s for the first injection, 4.0 s for the rest, inject spacing was 150 s.

SNA. For SNA binding to **G21**, the SNA solution (50 μM) in the sample cell was titrated with a solution of **G21 (**6SL, 0.5 mM). The reference power was 5 μW. For SNA binding to human transferrin (TF), SNA (21 μM) in the sample cell was titrated with TF (0.5 mM) or NeuC (Sigma-Aldrich, Oakville, Canada)-treated TF (0.3 mM); the reference power was 20 μW. For SNA binding to free *N*-glycans (released from TF and NeuC-treated TF using PNGase F), SNA (10 μM) in the sample cell was titrated with *N*-glycans from TF (0.3 mM) or NeuC-treated TF (0.1 mM); the reference power was 30 μW.

MAA. For the MAA binding to **G22** (3SL), MAA solution (38.5 μM) in the sample cell was titrated with solution of **G22** (0.5 mM); the reference power was 20 μW.

RCA-I. For RCA-I binding to **G34** glycan, RCA-I (3 μM) in cell was titrated with **G34** (30 μM); the reference power was 30 μW.

HILIC-UHPLC-ESI-MS analysis. Glycans were labeled with procainamide, as described previously, prior to LC analysis.[S3] HILIC analysis of the glycan libraries was performed using a Thermo Scientific™ Vanquish™ UHPLC system coupled with fluorescent (FLD) detector (Thermo Scientific, Waltham, MA, USA) and ESI-MS (Thermo Q Exactive Orbitrap). The glycans were separated HILIC using a Waters BEH Glycan column 150x2.1 mm i.d, with BEH particles size of 1.7 μm. The eluents were ammonium formate 100 mM (pH 4.5) (A) and acetonitrile (B). Separation was performed at a flow rate of 0.2 mL min^{-1} with following gradients: 0-46.5 min, 75-65% B; 46.5-80 min, 65-55% B; 80-104.6 min, 55-50% B. The injection volume was 4 μL. The column compartment temperature was maintained at 60 ^{o}C.

The fluorescence excitation and detection wavelengths were λ_{ex} = 310 and λ_{em} = 370 nm. The ESI-MS parameters were set as followed: sheath gas flow rate, 40 arb, capillary temperature, 275 ^{o}C, probe heater temperature, 250 ^{o}C, aux gas flowrate,10 arb, spray voltage, 2.5 kV. The *m/z* range was 250-3000 in positive mode. The resolution for full MS analysis was at 70000. The maximum injection time was 100 ms and the automatic gain control (AGC) target was at $1x10^{6}$. The MS spectra were recorded using Xcalibur (Thermo, Version 4.1).

The glycan composition corresponding to each peak identified by HILIC analysis was preliminarily assigned according to the MS, MS/MS data with the aid of the Glycoworkbench software and known biosynthetic pathways of *N*-glycan.[S4] The structures of separated isomers were then assigned according to the measured retention times (known as Glucose Unit (GU)).[S4,S5] In addition to the GU, the linkage and branch of the sialic acid were confirmed by treating the glycans with NeuS.[S6]

Mass Spectrometry.

Measurements were performed on a Q-Exactive Orbitrap mass spectrometer (Classic) and a Q Exactive Ultra-High Mass Range (UHMR) Orbitrap mass spectrometer (Thermo Fisher Scientific, Bremen, Germany). Both instruments were equipped with a modified nanoflow ESI source. NanoESI tips were produced from borosilicate glass capillaries (1.0 mm outside diameter (o.d.), 0.78 mm inner diameter (i.d.), 10 cm length or 1.2 mm o.d., 0.69 mm i.d., 10 cm length) using a P-1000 micropipette puller or a P-97 micropipette puller (Sutter Instruments, CA). To perform nanoESI, a voltage of approximately +0.5 to +0.9 kV was applied to a platinum wire inserted inside the nanoESI tip and in contact with the solution. The solution temperature was 25 ^{o}C. Resolutions (resolving power) of 12,500 or 17,500 were used for most of the protein-glycan systems except for DC-SIGN CRD (where resolution of 140,000 was used) for direct binding measurements. For the CaR-ESI-MS measurements, a resolving power of 25,000 was used. Maximum injection time was 200 ms, the S-lens RF level was 100 and DC offset was 20-100. Raw data were processed using the Thermo Xcalibur 4.2 software. Time-resolved mass spectra were averaged over 1 min intervals and the sum of the charge state-normalized abundances of the reactant and the complex ions were calculated automatically using the SWARM software (https://github.com/pkitov/CUPRA-SWARM).[S7] For mass spectra acquired for the glycoprotein sample, which exhibit poorly resolved or unresolved peaks, as well as spectral overlap between free and bound species, analysis was performed using charge state-normalized peak areas that were determined by fitting the ion signal with Gaussian functions using the Igor pro multifit tool (WaveMetrics Inc., Lake Oswego, OR, USA) and SWARM. Glycopeptides were confirmed by molecular weight, m/z and isotopic pattern obtained by Bruker DataAnalysis software.

Calculation of glycan concentration in diffusion experiment in the presence of GBP

The time-resolved glycan concentrations were calculated from the known affinity (K_d) of the glycan ligand for the GBP and the relative abundance of free and ligand-bound GBP ions obtained from ESI-MS measurements (eq S1):

$$[\mathrm{L}] = K_{\mathrm{d}}R + \frac{[\mathrm{P}]_0 R}{1+R} \quad \text{(S1)}$$

where $[P]_0$ is the initial GBP concentration and R is the abundance ratio of free-to-bound GBP (eq 3).

Assessing the dynamic range of COIN-CaR-nMS. To assess the dynamic range of COIN-CaR-nMS, affinity measurements were performed on Sig7-Fc and SNA and a mixture of two ligands (6SL (**G21**) and GD2 (**G29**) (Sig7-Fc) and 6SL (**G21**) and 6SLN (**G23**) (SNA)). For these experiments, one ligand was maintained at a fixed concentration (30 μM), the concentration of the other was varied from 10 μM to 10 nM.

Pseudovirus production and transduction and Pharmacological modulation of *N*-glycan type. Pseudovirus production and transduction were performed as described previously.[S8] Pharmacological modulation of *N*-glycan type was conducted as following: stocks of kifunensine (Sigma-Aldrich) were prepared at 5 mM concentration in sterile H_2O. Approximately 70,000 cells in 250 μL of Dulbecco's Modified Eagle Medium (DMEM) growth media (Gibco) containing 10% Fetal Bovine Serum (FBS, Gibco), 100 U/mL Penicillin (Gibco), 100 μg/mL Streptomycin (Gibco), and μg/mL Blasticidin (InvivoGen), were seeded in a 24-well plate in triplicate. Kifunensine, or H_2O as a negative control, was added to wells at 1:1000 dilution (5 μM). Cells were incubated at 37 ºC, 5% CO2 for 72 hours.

SARS-CoV-2 infection assays

All experiments with SARS-CoV-2 virus were performed under biosafety level 3 (BSL3) conditions. ACE2$^+$ HEK293 cells were cultured using Complete DMEM supplemented with 10% FBS and Blasticidin.[S8] SARS-CoV-2 (hCoV-19/Canada/ON-VIDO-01/2020, GISAID accession EPI_ISL_425177) was kindly provided by D. Falzarano (Vaccine and Infectious Disease Organization).

ACE2$^+$ HEK293 cells were seeded in 96-well plates (1×10^4 cells per well), and the next day, the cells were treated with kifunesine or H_2O and remdesivir as a positive control for inhibiting SARS-CoV-2 replication. After 72 h, cells were infected with the indicated strains of SARS-CoV2 using an MOI of 0.1 . The virus-containing inoculum was removed after 1 h and replaced with complete fresh medium. Cells were incubated for an additional 24 h before collection of the medium for a plaque assay. Briefly, approximately 1×10^5 Vero cells were seeded in a 24-well plate. After a 24-h incubation, cells were infected with serial dilutions of virus-containing medium (0.2 ml) for 1 h with occasional rocking, after which 1.0 ml of medium containing 0.75% carboxymethyl cellulose was added to the wells. Cells were incubated for 72–96 h to allow plaque formation, after which samples were fixed with 10% formaldehyde, washed with water and stained with 1% crystal violet in 20% ethanol. After extensive washing with water, plaques were counted, and viral titers (PFU per ml) were determined.

Tables

Table S1. Glycan ID, chemical composition, symbol nomenclature (SNFG) and molecular weight (MW) of purified glycans used in the study.[a]

Glycan ID	Chemical composition	Symbol structure	MW
**G1**	α-D-Gal-(1→3)-[α-L-Fuc(1→2)]-β-D-Gal(1→4)-D-GlcNAc	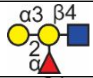	691.25
**G2**	α-L-Fuc-(1→2)-β-D-Gal-(1→4)-D-Glc	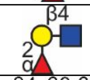	488.17
**G3**	(Gal-(1→4)-[α-L-Fuc(1→3)]-β-D-GlcNAc-(1→3)-β-D-Gal(1→4)[α-L-Fuc(1→3)]-D-Glc	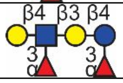	999.36
**G4**	[α-D-Neu5Ac-(2→6)-β-D-Gal-(1→4)-β-D-GlcNAc-(1→2)-α-D-Man-(1→3)]-[α-D-Neu5Ac-(2→6)-β-D-Gal-(1→4)-β-D-GlcNAc-(1→2)-α-D-Man-(1→6)]-β-D-Man(1→4)-β-D-GlcNAc-(1→4)-D-GlcNAc	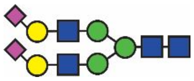	2222.78
**G5**	α-D-Man-(1→2)-α-D-Man-(1→6)-[Man-(1→2)-α-D-Man-(1→3)]-α-D-Man-(1→6)-[Man-(1→2)-α-D-Man-(1→2)-α-D-Man-(1→3)]-α-D-Man	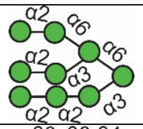	1476.48
**G6**	β-D-Gal(1→3)-β-D-GlcNAc(1→3)- β-D-Gal(1→4)-[α-L-Fuc(1→3)] -β-D-Glc	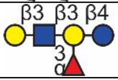	853.31
**G7**	β-D-Gal-(1→4)-β-D-GlcNAc(1→3)-β-D-Gal(1→4)-β-D-GlcNAc(1→3)-β-D-Gal(1→4)-β-D-Glc	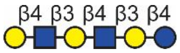	1072.38
**G8**	β-D-Gal-(1→4)-β-D-GlcNAc-(1→3)-β-D-Gal-(1→4)-β-D-GlcNAc-(1→3)-β-D-Gal-(1→4)-β-D-GlcNAc-(1→3)-β-D-Gal-(1→4)-β-D-Glc	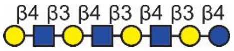	1437.51
**G9**	β-D-Gal-(1→4)-β-D-GlcNAc-(1→3)-β-D-Gal-(1→4)-β-D-Glc	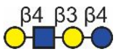	707.25
**G10**	α-D-GalNAc(1→3)-[α-L-Fuc(1→2)]-β-D-Gal(1→4)-D-GlcNAc	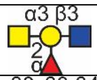	732.28
**G11**	β-D-Gal(1→3)-[α-L-Fuc(1→4)]-β-D-GlcNAc(1→3)-β-D-Gal(1→4)-[α-L-Fuc(1→3)]-β-D-Glc	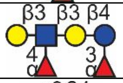	999.36
**G12**	α-D-GalNAc-(1→3)-[α-L-Fuc-(1→2)]-β-D-Gal-(1→4)-β-D-Glc	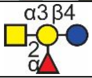	691.25
**G13**	Gly037-1 α-D-GalNAc-(1→3)-[α-L-Fuc-(1→2)]-β-D-Gal-(1→3)-β-D-GlcNAc-(1→3)-β-D-Gal-(1→4)-β-D-Glc	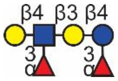	1056.39
**G14**	α-D-Man-(1→2)-α-D-Man-(1→6)-[α-D-Man-(1→3)]-α-D-Man-(1→6)-[α-D-Man-(1→2)-α-D-Man-(1→2)-α-D-Man-(1→3)]-α-D-Man	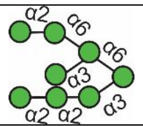	1314.43
**G15**	α-D-Man-(1→2)-α-D-Man-(1→3)-[α-D-Man-(1→6)-[α-D-Man-(1→2)-α-D-Man-(1→3)]-α-D-Man	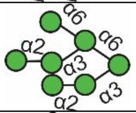	1152.38
**G16**	α-D-Man-(1→3)[α-D-Man(1→6)]-α-D-Man(1→6)[α-D-Man(1→2)-α-D-Man-(1→3)]-α-D-Man	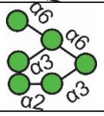	990.32

**G17**	α-L-Fuc-(1→2)-β-D-Gal-(1→3)-[α-L-Fuc(1→4)]-β-D-GlcNAc(1→3)-Gal	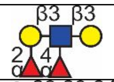	837.31
**G18**	α-D-Gal-(1→3)-[α-L-Fuc(1→2)]- β-D-Gal(1→4)-D-GlcNAc-β-D-Gal-(1→4)-β-D-Glc	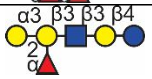	1015.36
**G19**	Manα(1-3)[Manα(1-6)]Manα(1-6)Man	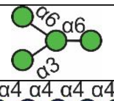	666.22
**G20**	α-D-Glc-(1→4)-α-D-Glcα1-4Glcα1-4Glcα1-4Glc	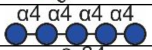	828.27
**G21**	α-D-Neu5Ac-(2→6)-β-D-Gal-(1→4)-D-Glc	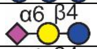	633.21
**G22**	α-D-Neu5Ac-(2→3)-β-D-Gal-(1→4)-D-Glc	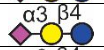	633.21
**G23**	α-D-Neu5Ac-(2→6)-β-D-Gal-(1→4)-D-GlcNAc	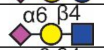	674.24
**G24**	α-D-GalNAc(1→3)-[α-L-Fuc(1→2)]-β-Gal(1→4)-D-GlcNAc	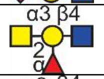	732.28
**G25**	α-D-Neu5Ac-(2→3)-β-D-Gal-(1→4)-D-GlcNAc	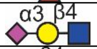	674.24
**G25**	α-D-Neu5Ac-(2→8)-α-D-Neu5Ac-(2→3)-β-D-Gal-(1→4)-D-Glc (GD3)	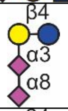	924.30
**G27**	α-D-Neu5Ac-(2→8)-α-D-Neu5Ac-(2→8)-α-D-Neu5Ac-(2→3)-β-D-Gal-(1→4)-D-Glc (GT3)	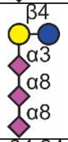	1215.40
**G28**	α-D-GalNAc(1→4)-[α-D-Neu5Ac-(2→3)]-β-D-Gal-(1→4)-D-Glc (GM2)	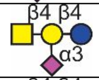	836.29
**G29**	α-D-GalNAc(1→4)-[α-D-Neu5Ac-(2→8)-α-D-Neu5Ac-(2→3)]-β-D-Gal-(1→4)-D-Glc (GD2)	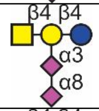	1127.38
**G30**	α-D-GalNAc(1→4)-[α-D-Neu5Ac-(2→8)-α-D-Neu5Ac-(2→8)-α-D-Neu5Ac-(2→3)]-β-D-Gal-(1→4)-D-Glc (GT2)	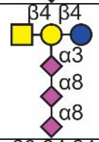	1418.48
**G31**	β-D-Gal(1→3)-α-D-GalNAc(1→4)-[α-D-Neu5Ac-(2→3)]-β-D-Gal-(1→4)-D-Glc (GM1)	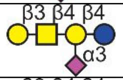	998.34
**G32**	β-D-Gal(1→3)-α-D-GalNAc(1→4)-[α-D-Neu5Ac-(2→8)-α-D-Neu5Ac-(2→3)]-β-D-Gal-(1→4)-D-Glc (GD1b)	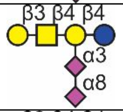	1289.45
**G33**	β-D-Gal(1→3)-α-D-GalNAc(1→4)-[α-D-Neu5Ac-(2→8)-α-D-Neu5Ac-(2→8)-α-D-Neu5Ac-(2→3)]-β-D-Gal-(1→4)-D-Glc (GT1c)	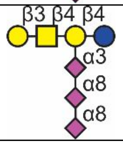	1580.53
**G34**	[β-D-GlcNAc-(1→2)-α-D-Man-(1→3)]-[β-D-GlcNAc-(1→2)-α-D-Man-(1→6)]-β-D-Man(1→4)-β-D-GlcNAc-(1→4)-D-GlcNAc	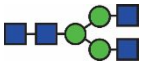	1316.49

[a] SNFG = symbol nomenclature for glycans.

Table S2. Affinities (K_d, µM) measured by nMS and COIN-nMS for GAL-3C binding with **G1** and **G2**, GAL-7 homodimer with **G3**, fCD22 with **G4** and DC-SIGN CRD with **G5**. Literature K_d are given as reference. Measurements were performed on ammonium acetate solutions (200 mM, pH 6.9) for GAL-3C, GAL-7 and fCD22 and a solution of ammonium acetate (200 mM) and calcium carbonate (2.5 mM) at pH 7.4 for DC-SIGN CRD.[a,b]

GBP	Glycan ID	K_d (nMS)	Concentration (µM)	K_d (COIN-nMS, individual experiments)	K_d (COIN-nMS, global fit)	K_d (Literature)
GAL-3C	**G1**	4.2±0.28	20<br>30<br>50	3.2±0.1<br>3.7±0.1<br>5.8±0.1	3.8±0.23	4.5±0.3 [S9]
GAL-3C	**G2**	26.6±1.7	20<br>30<br>50	27.0±0.5,<br>24.8±0.5<br>28.0±0.8	23.2±1.8	47.6 ± 4.5 [S10]
GAL-7	**G3**	518.3±15.2	20<br>30<br>50	524±2<br>507±6<br>537±6	521.3±24.9	500 ± 250 [S10]
fCD22	**G4**	13.1±0.6	30<br>50<br>75	25.4±2.2,<br>37.5±0.5,<br>23.4±0.7	23.5±1.4	13.1 ± 0.6 [S3]
DC-SIGN CRD	**G5**	231.0±17.3	75<br>100<br>125	212.4±2.2,<br>246.7±1.0,<br>234.0±1.5	234.8±12.6	NA

[a] Errors correspond to one standard deviation.

[b] NA ≡ not applicable.

Table S3. Affinities (K_d, μM) measured by nMS and COIN-nMS for GAL-3C binding to glycans (**G2**, **G5-G13**) present as a mixture. Measurements were performed on ammonium acetate solutions (200 mM, pH 6.9). [a,b]

Glycan ID	K_d (nMS)	K_d (COIN-nMS)	Stock concentration (μM)
**G2**	165.7 ± 84.5	156.1 ± 48.6	100
**G5**	NB	NB	100
**G6**	26.8 ± 4.7	101.2 ± 18.0	60
**G7**	5.0 ± 1.8	2.9 ± 0.9	60
**G8**	5.5 ± 1.3	5.8 ± 0.6	100
**G9**	23.2 ± 10.1	12.9 ± 7.4	40
**G10**	17.9 ± 3.0	10.9 ± 3.1	150
**G11**	NB	NB	100
**G12**	11.3 ± 2.1	10.4 ± 3.6	60
**G13**	4.6 ± 0.9	2.4 ± 0.6	100

[a] Errors correspond to one standard deviation. Different dilutions (2, 4, 5 or10 times) of the glycan mixture (with the listed stock concentrations) were used. The K_d values are the average of eight measurements (duplicates for each dilution)

[b] NB ≡ No binding detected.

Table S4. Affinities (K_d, µM) measured by nMS and COIN-nMS for GAL-7 homodimer binding to glycans (**G2**, **G5-G13**) present as a mixture. Measurements were performed on ammonium acetate solutions (200 mM, pH 6.9). [a,b]

Glycan ID	K_d (nMS)	K_d (COIN-nMS)	Stock concentration (µM)
**G2**	237.4 ± 69.3	241.1 ±10.7	100
**G5**	NB	NB	100
**G6**	232.4 ± 52.23	277.6 ± 84.8	60
**G7**	348.0 ± 89.2	361.7 ± 143.3	60
**G8**	177.6 ± 54.3	231.7 ± 24.9	100
**G9**	348.4 ± 62.8	439.2 ±158.1	40
**G10**	730.6 ± 141.3	436.6 ± 82.4	150
**G11**	NB	NB	100
**G12**	184.3 ± 15.7	333.2 ± 116.8	60
**G13**	261.0 ± 22.65	260.7 ± 119.4	100

[a] Errors correspond to one standard deviation. Different dilutions (2, 4, 5 or 10 times) of the glycan mixture (with the listed stock concentrations) were used. The K_d values are the average of eight measurements (duplicates for each dilution)

[b] NB ≡ No binding detected.

Table S5. Affinities (K_d, mM) measured by nMS and COIN-nMS for DC-SIGN CRD binding to glycans (**G2**, **G5**, **G10**, **G14-G20**) present as a mixture. Measurements were performed on ammonium acetate (200 mM) and calcium carbonate (2.5 mM) at pH 7.4.[a]

Glycan ID	K_d (nMS)	K_d (COIN-nMS)	Stock concentration (µM)
**G2**	2.10 ± 0.19	0.66 ± 0.04	200
**G5**	0.23 ± 0.02	0.24 ± 0.04	300
**G10**	6.01 ± 0.08	2.92 ± 1.17	100
**G14**	0.27 ± 0.01	0.26 ± 0.03	200
**G15**	0.39 ± 0.05	0.41 ± 0.04	200
**G16**	0.81 ± 0.06	0.56 ± 0.03	400
**G17**	0.62 ± 0.03	0.55 ± 0.05	100
**G18**	3.33 ± 0.55	1.87 ± 0.36	100
**G19**	1.18 ± 0.05	1.50 ± 0.23	100
**G20**	2.41 ± 0.06	1.83 ± 0.54	200

[a] Errors correspond to one standard deviation. Different dilutions (2, 4 or 10 times) of the glycan mixture (with the listed stock concentrations) were used. The K_d values are the average of six measurements (duplicates for each dilution)

Table S6. Affinities (K_d, μM) measured by COIN-CaR-nMS for SNA binding with **G21**, MAA with **G22**, CD22-Fc with **G21** and GNA with **G5**.[a] Measurements were performed using ammonium acetate solutions (200 mM, pH 6.9).

GBP	Glycan ID	K_d (COIN-CaR-nMS)	K_d (Literature)
SNA	**G21**	1.6 ± 0.4	1.98 ± 0.27 [b]
MAA	**G22**	3.5 ± 1.5	3.6 ± 2.0 [b]
CD22-Fc	**G21**	40.0 ± 1.0	75 ± 4 [c]
GNA	**G5**	553.2 ± 1.0	774.1 ± 12.8 [d]

[a] Errors correspond to one standard deviation.
[b] Values determined by ITC.
[c] Value, determined by direct MS (nMS) on CD22 fragment (fCD22), taken from ref S11.
[d] Value determined by direct nMS.

Table S7. Affinities (K_d, μM) measured by COIN-CaR-nMS for SNA binding with **G23** (0.05 - 10 μM) and **G21** (30 μM), and for Sig7-Fc with **G21** (0.1 - 10 μM) and **G29** (30 μM).[a] Measurements were performed using ammonium acetate solutions (200 mM, pH 6.9).

GBP	G23 concentration (μM)	G21 concentration (μM)	G29 concentration (μM)	K_d G21	K_d G23 (SNA)	K_d G29 (Sig7-Fc)
SNA	10	30	-	2.0 ± 0.4	0.7 ± 0.2	-
SNA	5	30	-	1.5 ± 0.3	0.9 ± 0.1	-
SNA	1.0	30	-	3.3 ± 0.2	0.4 ± 0.1	-
SNA	0.5	30	-	1.7 ± 0.5	0.3 ± 0.1	-
SNA	0.1	30	-	1.6 ± 0.4	0.4 ± 0.1	-
SNA	0.05	30	-	1.8 ± 0.5	0.6 ± 0.3	-
Sig7-Fc	-	10	30	249 ± 20	-	240 ± 10
Sig7-Fc	-	5	30	366 ± 10	-	240 ± 2
Sig7-Fc	-	1.0	30	239 ± 50	-	184 ± 5
Sig7-Fc	-	0.5	30	284 ± 70	-	215 ± 20

[a] Errors correspond to one standard deviation.

Table S8. Annotation of *N*- and *O*- linked glycopeptides obtained from pronase digestion of bovine fetuin (Uniprot protein sequence P12763).[a,b]

No.	m/z	Peptides	Glycan composition	Ion type	Δ m/z
1	760.272	S	$HexNAc_1Hex_1NeuAc_1$	$[M-H]^-$	
2	773.014	NC	$HexNAc_5Hex_6NeuAc_3$	$[M-4H]^{4-}$	0.001
3	776.018	ND	$HexNAc_5Hex_6NeuAc_3$	$[M-4H]^{4-}$	0.002
4	783.275	NGS	$HexNAc_5Hex_6NeuAc_3$	$[M-4H]^{4-}$	0.002
5	787.274	NGS	$HexNAc_5Hex_6NeuAc_2NeuGc_1$	$[M-4H]^{4-}$	0.002
6	793.274	LAN	$HexNAc_5Hex_6NeuAc_3$	$[M-4H]^{4-}$	0.019
7	797.777	NDS	$HexNAc_5Hex_6NeuAc_3$	$[M-4H]^{4-}$	0.001
8	800.322	^{269}(A)PSAVPD(A)^{276}	$HexNAc_1Hex_1NeuAc_2$	$[M-2H]^{2-}$	0.001
9	802.765	^{99}NC^{100}↔ ^{89}C	$HexNAc_5Hex_6NeuAc_3$	$[M-4H]^{4-}$	0.003
10	805.033	SNGS	$HexNAc_5Hex_6NeuAc_3$	$[M-4H]^{4-}$	0.003
11	806.764	^{99}NC^{100}↔ ^{89}C	$HexNAc_5Hex_6NeuAc_2NeuGc_1$	$[M-4H]^{4-}$	0.001
12	811.314	^{269}(A)PSAVPD(A)^{276}	$HexNAc_1Hex_1NeuAc_2$	$[M-3H+Na]^{2-}$	0.001
13	812.280	NC	$HexNAc_4Hex_5NeuAc_2$	$[M-3H]^{3-}$	0.001
14	816.283	ND	$HexNAc_4Hex_5NeuAc_2$	$[M-3H]^{3-}$	0.001
15	836.801	^{56}NDSR^{159}	$HexNAc_5Hex_6NeuAc_3$	$[M-4H]^{4-}$	0.003
16	839.289	LAN	$HexNAc_4Hex_5NeuAc_2$	$[M-3H]^{3-}$	0.030
17	845.293	NDS	$HexNAc_4Hex_5NeuAc_2$	$[M-3H]^{3-}$	0.002
18	851.945	^{99}NC^{100}↔ ^{89}C	$HexNAc_4Hex_5NeuAc_2$	$[M-3H]^{3-}$	0.002
19	856.048	NGS	$HexNAc_5Hex_6NeuAc_4$	$[M-4H]^{4-}$	0.002
20	859.342	SV	$HexNAc_1Hex_1NeuAc_1$	$[M-H]^-$	0.002
21	870.550	NDS	$HexNAc_5Hex_6NeuAc_4$	$[M-4H]^{4-}$	0.002
22	877.807	SNGS	$HexNAc_5Hex_6NeuAc_4$	$[M-4H]^{4-}$	0.003
23	881.320	SV	$HexNAc_1Hex_1NeuAc_1$	$[M-2H+Na]^-$	0.002
24	883.839	^{278}GPTPS^{282}	$2x(HexNAc_1Hex_1NeuAc_1)$	$[M-2H]^{2-}$	0.005
25	897.331	^{156}NDSR^{159}	$HexNAc_4Hex_5NeuAc_2$	$[M-3H]^{3-}$	0.003
26	909.574	^{56}NDSR^{159}	$HexNAc_5Hex_6NeuAc_4$	$[M-4H]^{4-}$	0.004
27	919.357	^{277}(A)GPTPS(A)^{282}	$HexNAc_2Hex_2NeuAc_2$	$[M-2H]^{2-}$	0.007
28	928.364	^{270}PSA^{272}	$HexNAc_1Hex_1NeuAc_1$	$[M-H]^-$	0.000
29	933.991	NC	$HexNAc_5Hex_6NeuAc_1$	$[M-3H]^{3-}$	0.002
30	937.995	ND	$HexNAc_5Hex_6NeuAc_1$	$[M-3H]^{3-}$	0.000
31	947.668	NGS	$HexNAc_5Hex_6NeuAc_1$	$[M-3H]^{3-}$	0.002
32	958.669	-	$HexNAc_5Hex_6NeuAc_3$	$[M-3H]^{3-}$	0.001
33	961.000	LAN	$HexNAc_5Hex_6NeuAc_1$	$[M-3H]^{3-}$	0.026
34	967.006	^{156}NDS^{158}	$HexNAc_5Hex_6NeuAc_2$	$[M-3H]^{3-}$	0.004
35	973.660	^{99}NC^{100}↔ ^{89}C	$HexNAc_5Hex_6NeuAc_2$	$[M-3H]^{3-}$	0.007
36	982.890	^{269}(A)PSAVPD(A)^{276}	$HexNAc_2Hex_2NeuAc_2$	$[M-2H]^{2-}$	0.000
37	993.880	^{269}(A)PSAVPD(A)^{276}	$HexNAc_2Hex_2NeuAc_2$	$[M-3H+Na]^{2-}$	0.002
38	996.352	NGS	$HexNAc_5Hex_6NeuAc_2Fuc_1$	$[M-3H]^{3-}$	0.005
39	996.688	N	$HexNAc_5Hex_6NeuAc_3$	$[M-3H]^{3-}$	0.001

40	1004.020	N	$HexNAc_5Hex_6NeuAc_3$	$[M-4H+Na]^{3-}$	0.001
41	1009.688	LAN	$HexNAc_5Hex_6NeuAc_2Fuc_1$	$[M-3H]^{3-}$	0.025
42	1015.695	^{176}NG^{177}	$HexNAc_5Hex_6NeuAc_3$	$[M-3H]^{3-}$	0.002
43	1019.04	^{156}NDSR^{159}	$HexNAc_5Hex_6NeuAc_2$	$[M-3H]^{3-}$	0.005
44	1022.350	^{99}NC^{100}↔ ^{89}C	$HexNAc_5Hex_6NeuAc_2Fuc_1$	$[M-3H]^{3-}$	0.005
45	1025.692	SN	$HexNAc_5Hex_6NeuAc_3$	$[M-3H]^{3-}$	0.003
46	1035.026	ND	$HexNAc_5Hex_6NeuAc_3$	$[M-3H]^{3-}$	0.001
47	1039.700	LN	$HexNAc_5Hex_6NeuAc_2NeuGc_1$	$[M-3H]^{3-}$	0.009
48	1041.682	NC	$HexNAc_5Hex_6NeuAc_2NeuGc_1$	$[M-3H]^{3-}$	0.002
49	1044.708	^{176}NGS^{178}	$HexNAc_5Hex_6NeuAc_3$	$[M-3H]^{3-}$	0.002
50	1050.034	NGS	$HexNAc_5Hex_6NeuAc_2NeuGc_1$	$[M-3H]^{3-}$	0.002
51	1052.035	^{176}NGS^{178}	$HexNAc_5Hex_6NeuAc_3$	$[M-4H+Na]^{3-}$	0.002
52	1058.033	LAN	$HexNAc_5Hex_6NeuAc_3$	$[M-3H]^{3-}$	0.025
53	1064.043	^{156}NDS^{158}	$HexNAc_5Hex_6NeuAc_3$	$[M-3H]^{3-}$	0.002
54	1070.693	^{99}NC^{100}↔ ^{89}C	$HexNAc_5Hex_6NeuAc_3$	$[M-3H]^{3-}$	0.003
55	1076.019	^{99}NC^{100}↔ ^{89}C	$HexNAc_5Hex_6NeuAc_3$	$[M-4H+NH_3]^{3-}$	0.011
56	1078.019	^{99}NC^{100}↔ ^{89}C	$HexNAc_5Hex_6NeuAc_3$	$[M-4H+Na]^{3-}$	0.003
57	1112.45	^{278}GPTPS^{282}	$HexNAc_1Hex_1NeuAc_1$	$[M-H]^{-}$	0.002
58	1116.075	^{156}NDSR^{159}	$HexNAc_5Hex_6NeuAc_3$	$[M-3H]^{3-}$	0.001
59	1121.401	^{155}LND^{157}	$HexNAc_5Hex_6NeuAc_3Fuc_1$	$[M-3H]^{3-}$	0.008
60	1123.402	^{156}NDSR^{159}	$HexNAc_5Hex_6NeuAc_3$	$[M-4H+Na]^{3-}$	0.002
61	1128.731	^{155}LND^{157}	$HexNAc_5Hex_6NeuAc_3Fuc_1$	$[M-4H+Na]^{3-}$	0.008
62	1132.867	^{99}NC^{100}↔ ^{89}C	$HexNAc_4Hex_5NeuAc_1$	$[M-2H]^{2-}$	0.008
63	1134.431	^{278}GPTPS^{282}	$HexNAc_1Hex_1NeuAc_1$	$[M-2H+Na]^{-}$	0.002
64	1141.739	^{176}NGS^{178}	$HexNAc_5Hex_6NeuAc_4$	$[M-3H]^{3-}$	0.001
65	1147.060	^{176}NGS^{178}	$HexNAc_5Hex_6NeuAc_4$	$[M-4H+NH_3]^{3-}$	0.018
66	1149.064	^{176}NGS^{178}	$HexNAc_5Hex_6NeuAc_4$	$[M-4H+Na]^{3-}$	0.001
67	1154.401	^{176}NGS^{178}	$HexNAc_5Hex_6NeuAc_4$	$[M-4H+K]^{3-}$	0.009
68	1161.072	^{156}NDS^{158}	$HexNAc_5Hex_6NeuAc_4$	$[M-3H]^{3-}$	0.003
69	1167.416	N	$HexNAc_4Hex_5NeuAc_2$	$[M-2H]^{2-}$	0.004
70	1167.730	^{99}NC^{100}↔ ^{89}C	$HexNAc_5Hex_6NeuAc_4$	$[M-3H]^{3-}$	0.001
71	1170.746	SNGS	$HexNAc_5Hex_6NeuAc_4$	$[M-3H]^{3-}$	0.002
72	1195.924	NG	$HexNAc_4Hex_5NeuAc_2$	$[M-2H]^{2-}$	0.000
73	1213.103	^{156}NDSR^{159}	$HexNAc_5Hex_6NeuAc_4$	$[M-3H]^{3-}$	0.004
74	1224.934	^{156}ND^{157}	$HexNAc_4Hex_5NeuAc_2$	$[M-2H]^{2-}$	0.004
75	1268.446	^{156}NDS^{158}	$HexNAc_4Hex_5NeuAc_2$	$[M-2H]^{2-}$	0.001
76	1276.455	NDS	$HexNAc_4Hex_5NeuAc_1NeuGc_1$	$[M-2H]^{2-}$	0.015

77	1278.423	^{99}NC^{100}↔ ^{89}C	$HexNAc_4Hex_5NeuAc_2$	$[M-2H]^{2-}$	0.007
78	1305.455	NDS	$HexNAc_5Hex_6NeuAc_1$	$[M-2H]^{2-}$	0.006
79	1315.441	^{99}NC^{100}↔ ^{89}C	$HexNAc_5Hex_6NeuAc_1$	$[M-2H]^{2-}$	0.000
80	1346.493	^{156}NDSR^{159}	$HexNAc_4Hex_5NeuAc_2$	$[M-2H]^{2-}$	0.004
81	1362.494	^{156}NDSR^{159}	$HexNAc_4Hex_5NeuGc_2$	$[M-2H]^{2-}$	0.006
82	1422.004	NGS	$HexNAc_5Hex_6NeuAc_2$	$[M-2H]^{2-}$	0.002
83	1451.009	NDS	$HexNAc_5Hex_6NeuAc_2$	$[M-2H]^{2-}$	0.001
84	1460.985	^{99}NC^{100}↔ ^{89}C	$HexNAc_5Hex_6NeuAc_2$	$[M-2H]^{2-}$	0.003
85	1529.052	^{56}NDSR^{159}	$HexNAc_5Hex_6NeuAc_2$	$[M-2H]^{2-}$	0.007
86	1567.563	^{176}NGS^{178}	$HexNAc_5Hex_6NeuAc_3$	$[M-2H]^{2-}$	0.005
87	1606.551	^{99}NC^{100}↔ ^{89}C	$HexNAc_5Hex_6NeuAc_3$	$[M-2H]^{2-}$	0.006
88	1727.611	-	$HexNAc_3Hex_5NeuAc_1$	$[M-H]^{-}$	0.001
89	1839.702	^{277}(A)GPTPS(A)^{282}	$HexNAc_2Hex_2NeuAc_2$	$[M-H]^{-}$	0.006
90	1889.662	-	$HexNAc_3Hex_6NeuAc_1$	$[M-H]^{-}$	0.001
91	1930.693	-	$HexNAc_4Hex_5NeuAc_1$	$[M-H]^{-}$	0.001
92	2043.733	NDS	$HexNAc_3Hex_5NeuAc_1$	$[M-H]^{-}$	0.023
93	2069.765	LND	$HexNAc_3Hex_5NeuAc_1$	$[M-H]^{-}$	0.003
94	2074.728	AN	$HexNAc_3Hex_6NeuAc_1$	$[M-H]^{-}$	0.014
95	2321.830	ND	$HexNAc_4Hex_5NeuGc_1Fuc_1$	$[M-H]^{-}$	0.020
96	2434.889	LND	$HexNAc_4Hex_5NeuGc_1Fuc_1$	$[M-H]^{-}$	0.002

[a] Parentheses are used to indicate ambiguous assignment of the terminal amino acid residues; residue in red are the glycosylation sites.

[b] Presence of disulfide bond indicated by ↔.

Table S9. Annotation of *N*-linked glycopeptides obtained from pronase digestion of human lactoferrin (Sequence P02788 and P02788-2 with consideration of variants).

No	m/z	Peptides	Glycan composition	Ion type	Δ m/z
1	729.597	N	$HexNAc_4Hex_5NeuAc_1Fuc_1$	$[M-3H]^{3-}$	0.001
2	772.283	NQ	$HexNAc_4Hex_5NeuAc_1Fuc_1$	$[M-3H]^{3-}$	0.001
3	778.283	N	$HexNAc_4Hex_5NeuAc_1Fuc_2$	$[M-3H]^{3-}$	0.000
4	784.266	YN	$HexNAc_4Hex_5Fuc_3$	$[M-3H]^{3-}$	0.025
5	791.623	NW	$HexNAc_4Hex_5NeuAc_1Fuc_1$	$[M-3H]^{3-}$	0.001
6	820.631	NQ	$HexNAc_4Hex_5NeuAc_2$	$[M-3H]^{3-}$	0.002
7	826.631	N	$HexNAc_4Hex_5NeuAc_2Fuc_1$	$[M-3H]^{3-}$	0.003
8	832.642	YN	$HexNAc_4Hex_5NeuAc_1Fuc_2$	$[M-3H]^{3-}$	0.006
9	839.968	NW	$HexNAc_4Hex_5NeuAc_2$	$[M-3H]^{3-}$	0.000
10	840.308	NW	$HexNAc_4Hex_5NeuAc_1Fuc_2$	$[M-3H]^{3-}$	0.000
11	869.307	NQ	$HexNAc_4Hex_5NeuAc_2Fuc_1$	$[M-3H]^{3-}$	0.006
12	874.646	NGS	$HexNAc_4Hex_5NeuAc_2Fuc_1$	$[M-3H]^{3-}$	0.000
13	881.325	YN	$HexNAc_4Hex_5NeuAc_1Fuc_3$	$[M-3H]^{3-}$	0.002
14	888.644	NW	$HexNAc_4Hex_5NeuAc_2Fuc_1$	$[M-3H]^{3-}$	0.011
15	899.993	N	$HexNAc_5Hex_6NeuAc_1Fuc_2$	$[M-3H]^{3-}$	0.000
16	903.331	NGS	$HexNAc_3Hex_3NeuAc_1Fuc_1$	$[M-2H]^{2-}$	0.002
17	940.348	NQ	$HexNAc_4Hex_5$	$[M-2H]^{2-}$	0.001
18	948.679	N	$HexNAc_5Hex_6NeuAc_1Fuc_4$	$[M-3H]^{3-}$	0.000
19	976.349	NQ	$HexNAc_3Hex_4NeuAc_1Fuc_1$	$[M-2H]^{2-}$	0.008
20	984.357	NS	$HexNAc_4Hex_4NeuAc_1$	$[M-2H]^{2-}$	0.002
21	997.032	YNQ	$HexNAc_5Hex_6NeuAc_1Fuc_2$	$[M-3H]^{3-}$	0.002
22	1004.860	NQ	$HexNAc_4Hex_4NeuAc_1$	$[M-2H]^{2-}$	0.008
23	1013.377	NQ	$HexNAc_4Hex_5Fuc_1$	$[M-2H]^{2-}$	0.000
24	1013.856	N	$HexNAc_4Hex_4NeuAc_1Fuc_1$	$[M-2H]^{2-}$	0.012
25	1021.864	N	$HexNAc_4Hex_5NeuAc_1$	$[M-2H]^{2-}$	0.005
26	1022.372	N	$HexNAc_4Hex_5Fuc_2$	$[M-2H]^{2-}$	0.004
27	1065.374	NS	$HexNAc_4Hex_5NeuAc_1$	$[M-2H]^{2-}$	0.007
28	1077.897	NQ	$HexNAc_4Hex_4NeuAc_1Fuc_1$	$[M-2H]^{2-}$	0.000
29	1085.895	NQ	$HexNAc_4Hex_5NeuAc_1$	$[M-2H]^{2-}$	0.000
30	1094.895	N	$HexNAc_4Hex_5NeuAc_1Fuc_1$	$[M-2H]^{2-}$	0.001
31	1103.915	YN	$HexNAc_4Hex_5Fuc_2$	$[M-2H]^{2-}$	0.007
32	1106.891	NW	$HexNAc_4Hex_4NeuAc_1Fuc_1$	$[M-2H]^{2-}$	0.016
33	1128.407	NQT	$HexNAc_4Hex_4NeuAc_1Fuc_1$	$[M-2H]^{2-}$	0.013
34	1138.400	NS	$HexNAc_4Hex_5NeuAc_1Fuc$	$[M-2H]^{2-}$	0.010
35	1158.927	NQ	$HexNAc_4Hex_5NeuAc_1Fuc_1$	$[M-2H]^{2-}$	0.004
36	1167.926	N	$HexNAc_4Hex_5NeuAc_1Fuc_2$	$[M-2H]^{2-}$	0.008
37	1187.933	NW	$HexNAc_4Hex_5NeuAc_1Fuc_1$	$[M-2H]^{2-}$	0.001
38	1195.927	NG	$HexNAc_4Hex_5NeuAc_2$	$[M-2H]^{2-}$	0.003
39	1209.441	NQT	$HexNAc_4Hex_5NeuAc_1Fuc_1$	$[M-2H]^{2-}$	0.006
40	1231.953	NQ	$HexNAc_4Hex_5NeuAc_1Fuc_2$	$[M-2H]^{2-}$	0.001
41	1240.446	N	$HexNAc_4Hex_5NeuAc_2Fuc_1$	$[M-2H]^{2-}$	0.004
42	1245.978	RN	$HexNAc_4Hex_5NeuAc_1Fuc_2$	$[M-2H]^{2-}$	0.004

43	1251.43	N	$HexNAc_4Hex_5NeuAc_2Fuc_1$	$[M-3H+Na]^{2-}$	0.000
44	1260.96	NW	$HexNAc_4Hex_5NeuAc_1Fuc_2$	$[M-2H]^{2-}$	0.008
45	1268.950	NG	$HexNAc_4Hex_5NeuAc_2Fuc_1$	$[M-2H]^{2-}$	0.003
46	1282.463	NQT	$HexNAc_4Hex_5NeuAc_1Fuc_2$	$[M-2H]^{2-}$	0.013
47	1304.464	NQ	$HexNAc_4Hex_5NeuAc_2Fuc_1$	$[M-2H]^{2-}$	0.008
48	1312.459	NGS	$HexNAc_4Hex_5NeuAc_2Fuc_1$	$[M-2H]^{2-}$	0.009
49	1333.473	NW	$HexNAc_4Hex_5NeuAc_2Fuc_1$	$[M-2H]^{2-}$	0.009
50	1350.480	N	$HexNAc_5Hex_6NeuAc_1Fuc_2$	$[M-2H]^{2-}$	0.009
51	1370.485	NGSD	$HexNAc_4Hex_5NeuAc_1Fuc_3$	$[M-2H]^{2-}$	0.007
52	1414.505	NQ	$HexNAc_5Hex_6NeuAc_1Fuc_2$	$[M-2H]^{2-}$	0.014
53	1422.507	NGS	$HexNAc_5Hex_6NeuAc_1Fuc_2$	$[M-2H]^{2-}$	0.009
54	1423.511	N	$HexNAc_5Hex_6NeuAc_1Fuc_3$	$[M-2H]^{2-}$	0.008
55	1443.517	NW	$HexNAc_5Hex_6NeuAc_1Fuc_2$	$[M-2H]^{2-}$	0.012
56	1487.534	NQ	$HexNAc_5Hex_6NeuAc_1Fuc_3$	$[M-2H]^{2-}$	0.014
57	1495.533	NGS	$HexNAc_5Hex_6NeuAc_1Fuc_3$	$[M-2H]^{2-}$	0.012
58	1516.539	NW	$HexNAc_5Hex_6NeuAc_1Fuc_3$	$[M-2H]^{2-}$	0.020
59	2190.772	N	$HexNAc_4Hex_5NeuAc_1Fuc_1$	$[M-H]^-$	0.017
60	2318.830	NQ	$HexNAc_4Hex_5NeuAc_1Fuc_1$	$[M-H]^-$	0.017
61	2336.825	N	$HexNAc_4Hex_5NeuAc_1Fuc_2$	$[M-H]^-$	0.022

Table S10. Annotation of *N*-linked glycopeptides obtained from pronase digestion of bovine AGP.

No	m/z	Peptides	Glycan composition	Ion type	Δ m/z
1	1072.383	TN	$HexNAc_4Hex_5NeuAc_1$	$[M-2H]^{2-}$	0.006
2	1080.381	TN	$HexNAc_4Hex_5NeuGc_1$	$[M-2H]^{2-}$	0.005
3	1085.896	QN	$HexNAc_4Hex_5NeuAc_1$	$[M-2H]^{2-}$	0.002
4	1093.902	NK	$HexNAc_4Hex_5NeuGc_1$	$[M-2H]^{2-}$	0.008
5	1100.895	NGT	$HexNAc_4Hex_5NeuAc_1$	$[M-2H]^{2-}$	0.005
6	1108.892	NGT	$HexNAc_4Hex_5NeuGc_1$	$[M-2H]^{2-}$	0.005
7	1110.389	-	$HexNAc_4Hex_5NeuAc_2$	$[M-2H]^{2-}$	0.002
8	1118.384	-	$HexNAc_4Hex_5NeuGc_2$	$[M-2H]^{2-}$	0.005
9	1129.421	NKS	$HexNAc_4Hex_5NeuAc_1$	$[M-2H]^{2-}$	0.007
10	1137.421	NKS	$HexNAc_4Hex_5NeuGc_1$	$[M-2H]^{2-}$	0.006
11	1156.411	PNAT	$HexNAc_4Hex_5NeuAc_1$	$[M-2H]^{2-}$	0.025
12	1164.409	PNAT	$HexNAc_4Hex_5NeuGc_1$	$[M-2H]^{2-}$	0.029
13	1172.932	QNGT	$HexNAc_4Hex_5NeuGc_1$	$[M-2H]^{2-}$	0.000
14	1184.386	YN	$HexNAc_4Hex_5Fuc_1NeuGc_1$	$[M-2H]^{2-}$	0.039
15	1217.931	TN	$HexNAc_4Hex_5NeuAc_2$	$[M-2H]^{2-}$	0.006
16	1225.928	TN	$HexNAc_4Hex_5NeuAc_1Gc_1$	$[M-2H]^{2-}$	0.011
17	1231.446	QN	$HexNAc_4Hex_5NeuAc_2$	$[M-2H]^{2-}$	0.000
18	1233.931	TN	$HexNAc_4Hex_5NeuGc_2$	$[M-2H]^{2-}$	0.005
19	1239.449	QN	$HexNAc_4Hex_5NeuAc_1Gc_1$	$[M-2H]^{2-}$	0.004
20	1246.449	NGT	$HexNAc_4Hex_5NeuAc_2$	$[M-2H]^{2-}$	0.003
21	1247.453	QN	$HexNAc_4Hex_5NeuGc_2$	$[M-2H]^{2-}$	0.011
22	1254.439	NGT	$HexNAc_4Hex_5NeuAc_1Gc_1$	$[M-2H]^{2-}$	0.010
23	1262.438	NCS	$HexNAc_4Hex_5NeuAc_2$	$[M-2H]^{2-}$	0.002
24	1268.454	WN	$HexNAc_4Hex_5NeuAc_1Gc_1$	$[M-2H]^{2-}$	0.000
25	1274.972	NKS	$HexNAc_4Hex_5NeuAc_2$	$[M-2H]^{2-}$	0.001
26	1276.469	QN	$HexNAc_5Hex_6NeuAc_1$	$[M-2H]^{2-}$	0.006
27	1282.970	NKS	$HexNAc_4Hex_5NeuAc_1Gc_1$	$[M-2H]^{2-}$	0.009
28	1290.970	NKS	$HexNAc_4Hex_5NeuGc_2$	$[M-2H]^{2-}$	0.007
29	1301.964	PNAT	$HexNAc_4Hex_5NeuAc_2$	$[M-2H]^{2-}$	0.022
30	1309.957	PNAT	$HexNAc_4Hex_5NeuAc_1Gc_1$	$[M-2H]^{2-}$	0.028
31	1312.973	N	$HexNAc_4Hex_5NeuAc_3$	$[M-2H]^{2-}$	0.009
32	1317.956	PNAT	$HexNAc_4Hex_5NeuGc_2$	$[M-2H]^{2-}$	0.026
33	1320.962	N	$HexNAc_4Hex_5NeuAc_2Gc_1$	$[M-2H]^{2-}$	0.000
34	1326.483	QNGT	$HexNAc_4Hex_5NeuGc_2$	$[M-2H]^{2-}$	0.005
35	1329.451	EYN	$HexNAc_4Hex_5NeuGc_2$	$[M-2H]^{2-}$	0.015
36	1355.492	WNGT	$HexNAc_4Hex_5NeuGc_2$	$[M-2H]^{2-}$	0.004
37	1363.482	TN	$HexNAc_4Hex_5NeuAc_3$	$[M-2H]^{2-}$	0.007
38	1371.479	TN	$HexNAc_4Hex_5NeuAc_2Gc_1$	$[M-2H]^{2-}$	0.008
39	1377.006	NK	$HexNAc_4Hex_5NeuAc_3$	$[M-2H]^{2-}$	0.008
40	1379.477	TN	$HexNAc_4Hex_5NeuAc_1Gc_2$	$[M-2H]^{2-}$	0.007
41	1385.005	NK	$HexNAc_4Hex_5NeuAc_2Gc_1$	$[M-2H]^{2-}$	0.006
42	1881.718	QN	$HexNAc_4Hex_5$	$[M-H]^{-}$	0.016
43	1911.705	NGT	$HexNAc_4Hex_5$	$[M-H]^{-}$	0.007

44	2016.718	NCSF	$HexNAc_3Hex_4NeuAc_1$	$[M-H]^-$	0.000
45	2022.718	PNAT	$HexNAc_4Hex_5$	$[M-H]^-$	0.065
46	2056.691	NATM	$HexNAc_4Hex_5$	$[M-H]^-$	0.079
47	2032.710	YNCS	$HexNAc_3Hex_4NeuAc_1$	$[M-H]^-$	0.003
48	2048.711	YNCS	$HexNAc_3Hex_4NeuGc_1$	$[M-H]^-$	0.003
49	2067.744	PNA	$HexNAc_4Hex_5Fuc_1$	$[M-H]^-$	0.049
50	2085.738	WN	$HexNAc_4Hex_5Fuc_1$	$[M-H]^-$	0.042
51	2092.737	-	$HexNAc_4Hex_5Fuc_1NeuAc_1$	$[M-H]^-$	0.003
52	2172.773	QN	$HexNAc_4Hex_5NeuAc_1$	$[M-H]^-$	0.016

Table S11 Annotation of *N*- and *O*-linked glycopeptides obtained from pronase digestion of SARS-CoV-2 RBD.

No	m/z	Peptides	Glycan composition	Ion type	Δ m/z
1	826.624	N	$HexNAc_4Hex_5Fuc_1NeuAc_2$	$[M-3H]^{3-}$	0.004
2	850.297	NA	$HexNAc_4Hex_5Fuc_1NeuAc_2$	$[M-3H]^{3-}$	0.010
3	907.991	FPN	$HexNAc_4Hex_5Fuc_1NeuAc_2$	$[M-3H]^{3-}$	0.011
4	948.334	N	$HexNAc_5Hex_6Fuc_1NeuAc_2$	$[M-3H]^{3-}$	0.005
5	962.004	N	$HexNAc_6Hex_5Fuc_1NeuAc_2$	$[M-3H]^{3-}$	0.011
6	969.853	FPN	$HexNAc_3Hex_5Fuc_1$	$[M-2H]^{2-}$	0.014
7	972.011	NA	$HexNAc_5Hex_6Fuc_1NeuAc_2$	$[M-3H]^{3-}$	0.007
8	975.321	PNI	$HexNAc_4Hex_4Fuc_1$	$[M-2H]^{2-}$	0.065
9	976.860	NA	$HexNAc_4Hex_4Fuc_2$	$[M-2H]^{2-}$	0.008
10	981.358	FPN	$HexNAc_5Hex_6Fuc_2NeuAc_1$	$[M-3H]^{3-}$	0.008
11	984.858	NA	$HexNAc_4Hex_5Fuc_1$	$[M-2H]^{2-}$	0.007
12	997.018	N	$HexNAc_5Hex_6Fuc_2NeuAc_2$	$[M-3H]^{3-}$	0.006
13	1005.371	NA	$HexNAc_5Hex_4Fuc_1$	$[M-2H]^{2-}$	0.007
14	1013.859	N	$HexNAc_4Hex_4Fuc_1NeuAc_1$	$[M-2H]^{2-}$	0.009
15	1020.698	NA	$HexNAc_5Hex_6Fuc_2NeuAc_2$	$[M-3H]^{3-}$	0.006
16	1022.368	N	$HexNAc_5Hex_4Fuc_2$	$[M-2H]^{2-}$	0.007
17	1029.707	FPN	$HexNAc_5Hex_6Fuc_1NeuAc_2$	$[M-3H]^{3-}$	0.006
18	1034.374	FPN	$HexNAc_3Hex_4Fuc_1NeuAc_1$	$[M-2H]^{2-}$	0.014
19	1042.884	FPN	$HexNAc_3Hex_5Fuc_2$	$[M-2H]^{2-}$	0.013
20	1045.365	N	$HexNAc_5Hex_6Fuc_1NeuAc_3$	$[M-3H]^{3-}$	0.005
21	1049.377	NA	$HexNAc_4Hex_4Fuc_1NeuAc_1$	$[M-2H]^{2-}$	0.010
22	1057.888	NA	$HexNAc_5Hex_4Fuc_2$	$[M-2H]^{2-}$	0.006
23	1063.396	FPN	$HexNAc_4Hex_4Fuc_2$	$[M-2H]^{2-}$	0.014
24	1069.042	NA	$HexNAc_5Hex_6Fuc_1NeuAc_3$	$[M-3H]^{3-}$	0.008
25	1078.397	NA	$HexNAc_4Hex_5NeuAc_1Ac_1$	$[M-2H]^{2-}$	0.008
26	1086.397	NA	$HexNAc_5Hex_5Fuc_1$	$[M-2H]^{2-}$	0.008
27	1094.887	N	$HexNAc_4Hex_5Fuc_1NeuAc_1$	$[M-2H]^{2-}$	0.008
28	1115.401	N	$HexNAc_5Hex_4Fuc_1NeuAc_1$	$[M-2H]^{2-}$	0.005
29	1130.405	NA	$HexNAc_4Hex_5Fuc_1NeuAc_1$	$[M-2H]^{2-}$	0.008
30	1135.919	N	$HexNAc_6Hex_3Fuc_1NeuAc_1$	$[M-2H]^{2-}$	0.002
31	1143.414	PN	$HexNAc_4Hex_5Fuc_1NeuAc_1$	$[M-2H]^{2-}$	0.007
32	1150.914	NA	$HexNAc_5Hex_4Fuc_1NeuAc_1$	$[M-2H]^{2-}$	0.001
33	1155.381	N	$HexNAc_5Hex_4Fuc_1NeuAc_1S_1$	$[M-2H]^{2-}$	0.005
34	1164.942	FPN	$HexNAc_4Hex_5NeuAc_1Ac_1$	$[M-2H]^{2-}$	0.011
35	1167.915	N	$HexNAc_4Hex_5Fuc_2NeuAc_1$	$[M-2H]^{2-}$	0.008
36	1188.427	N	$HexNAc_4Hex_5NeuAc_1Ac_1$	$[M-2H]^{2-}$	0.001
37	1194.430	NITN	$HexNAc_6Hex_3Fuc_1S_1$	$[M-2H]^{2-}$	0.009
38	1196.423	N	$HexNAc_5Hex_5Fuc_1NeuAc_1$	$[M-2H]^{2-}$	0.011
39	1203.434	NA	$HexNAc_4Hex_5Fuc_2NeuAc_1$	$[M-2H]^{2-}$	0.007
40	1216.950	FPN	$HexNAc_4Hex_5Fuc_1NeuAc_1$	$[M-2H]^{2-}$	0.005
41	1223.945	NI	$HexNAc_4Hex_5NeuAc_2$	$[M-2H]^{2-}$	0.010
42	1231.947	NA	$HexNAc_5Hex_5Fuc_1NeuAc_1$	$[M-2H]^{2-}$	0.006

43	1237.461	FPN	$HexNAc_5Hex_4Fuc_1NeuAc_1$	$[M-2H]^{2-}$	0.003
44	1240.441	N	$HexNAc_4Hex_5Fuc_1NeuAc_2$	$[M-2H]^{2-}$	0.001
45	1257.969	FPN	$HexNAc_6Hex_3Fuc_1NeuAc_1$	$[M-2H]^{2-}$	0.012
46	1260.940	N	$HexNAc_5Hex_4Fuc_1NeuAc_2$	$[M-2H]^{2-}$	0.015
47	1275.953	NA	$HexNAc_4Hex_5Fuc_1NeuAc_2$	$[M-2H]^{2-}$	0.007
48	1277.456	N	$HexNAc_5Hex_6Fuc_1NeuAc_1$	$[M-2H]^{2-}$	0.005
49	1289.974	FPN	$HexNAc_4Hex_5Fuc_2NeuAc_1$	$[M-2H]^{2-}$	0.010
50	1310.484	FPN	$HexNAc_4Hex_5NeuAc_1Ac_1$	$[M-2H]^{2-}$	0.003
51	1312.973	NA	$HexNAc_5Hex_6Fuc_1NeuAc_1$	$[M-2H]^{2-}$	0.006
52	1318.487	FPN	$HexNAc_5Hex_5Fuc_1NeuAc_1$	$[M-2H]^{2-}$	0.008
53	1350.483	N	$HexNAc_5Hex_6Fuc_2NeuAc_1$	$[M-2H]^{2-}$	0.006
54	1362.494	FPN	$HexNAc_4Hex_5Fuc_1NeuAc_2$	$[M-2H]^{2-}$	0.008
55	1383.002	FPN	$HexNAc_5Hex_4Fuc_1NeuAc_2$	$[M-2H]^{2-}$	0.014
56	1385.995	NA	$HexNAc_5Hex_6Fuc_2NeuAc_1$	$[M-2H]^{2-}$	0.013
57	1391.508	FPN	$HexNAc_5Hex_5Fuc_2NeuAc_1$	$[M-2H]^{2-}$	0.016
58	1399.514	FPN	$HexNAc_5Hex_6Fuc_1NeuAc_1$	$[M-2H]^{2-}$	0.007
59	1418.506	NA	$HexNAc_2Hex_5$	$[M-H]^-$	0.007
60	1423.002	N	$HexNAc_5Hex_6Fuc_1NeuAc_2$	$[M-2H]^{2-}$	0.006
61	1458.514	NA	$HexNAc_5Hex_6Fuc_1NeuAc_2$	$[M-2H]^{2-}$	0.013
62	1472.540	FPN	$HexNAc_5Hex_6Fuc_2NeuAc_1$	$[M-2H]^{2-}$	0.010
63	1545.055	FPN	$HexNAc_5Hex_6Fuc_1NeuAc_2$	$[M-2H]^{2-}$	0.014
64	1545.560	ES	$HexNAc_2Hex_2NeuAc_2$	$[M-H]^-$	0.019
65	1646.607	TES	$HexNAc_2Hex_2NeuAc_2$	$[M-H]^-$	0.019

Table S12. Affinities (K_d, μM) measured by COIN-CaR-nMS screening of *N*-glycan (native and NeuS-treated) natural libraries and 6SL (**G21**) and 6SLN (**G23**) against to SNA. Measurements were performed on ammonium acetate solutions (200 mM, pH 6.9). [a,b]

**Order #**	**Glycan composition**	**Native library**	**NeuS-treated library**
1	$HexNAc_3Hex_4Fuc_1NeuAc_1$	0.13 ± 0.05	0.17 ± 0.04
2	$HexNAc_4Hex_4NeuAc_1$	0.14 ± 0.05	0.20 ± 0.07
3	$HexNAc_4Hex_4Fuc_1NeuAc_1$	0.14 ± 0.04	0.30 ± 0.05
4	$HexNAc_3Hex_4NeuAc_1$	0.15 ± 0.05	0.40 ± 0.14
5	$HexNAc_3Hex_4NeuGc_1$	0.15 ± 0.06	0.32 ± 0.10
6	$HexNAc_3Hex_5Fuc_1NeuAc_1$	0.16 ± 0.04	0.20 ± 0.05
7	$HexNAc_4Hex_5NeuAc_1$	0.20 ± 0.05	0.20 ± 0.07
8	$HexNAc_4Hex_5Fuc_1NeuAc_1$	0.20 ± 0.07	0.30 ± 0.03
9	$HexNAc_4Hex_5Fuc_2NeuAc_1$	0.20 ± 0.07	0.30 ± 0.02
10	$HexNAc_5Hex_3Fuc_1NeuAc_1$	0.20 ± 0.04	0.20 ± 0.04
11	$HexNAc_6Hex_3Fuc_2NeuAc_1$	0.20 ± 0.01	0.20 ± 0.01
12	$HexNAc_4Hex_5NeuGc_1$	0.40 ± 0.07	0.30 ± 0.03
13	$HexNAc_4Hex_5Fuc_1NeuGc_1$	0.41 ± 0.15	0.50 ± 0.17
14	$HexNAc_5Hex_4Fuc_1NeuAc_1$	0.50 ± 0.15	0.50 ± 0.04
15	α-D-Neu5Ac-(2→6)-β-D-Gal-(1→4)-D-GlcNAc	0.55 ± 0.16	-
16	$HexNAc_5Hex_6NeuAc_1$	0.65 ± 0.14	0.57 ± 0.14
17	$HexNAc_4Hex_5NeuAc_1NeuGc_1$	0.82 ± 0.07	1.20 ± 0.20
18	$HexNAc_5Hex_6NeuGc_1$	0.82 ± 0.21	0.40 ± 0.11
19	$HexNAc_4Hex_5NeuGc_2$	0.86 ± 0.22	1.40 ± 0.28
20	$HexNAc_5Hex_6Fuc_1NeuAc_2$	0.86 ± 0.07	1.02 ± 0.40
21	$HexNAc_6Hex_3Fuc_1NeuAc_1$	1.10 ± 0.10	0.41 ± 0.07
22	$HexNAc_5Hex_6NeuAc_2$	1.20 ± 0.07	0.54 ± 0.11
23	$HexNAc_4Hex_5Fuc_1NeuAc_2$	1.25 ± 0.48	1.00 ± 0.30
24	$HexNAc_5Hex_4Fuc_1NeuAc_1S$	1.30 ± 0.10	0.65 ± 0.32
25	$HexNAc_5Hex_4Fuc_1NeuAc_2$	1.35 ± 0.10	-
26	$HexNAc_4Hex_5Fuc_1NeuAc_1NeuGc_1$	1.36 ± 0.46	1.13 ± 0.07
27	$HexNAc_5Hex_6NeuAc_3$	1.42 ± 0.18	1.54 ± 0.52
28	α-D-Neu5Ac-(2→6)-β-D-Gal-(1→4)-D-Glc	1.60 ± 0.40	-
29	$HexNAc_6Hex_7Fuc_1NeuAc_2$	1.85 ± 0.10	-
30	$HexNAc_4Hex_5NeuAc_2$	2.07 ± 0.76	0.94 ± 0.16
31	$HexNAc_4Hex_5Fuc_1NeuGc_2$	2.25 ± 0.07	1.69 ± 0.38
32	$HexNAc_5Hex_6NeuAc_1NeuGc_1$	5.40 ± 0.50	0.79 ± 0.42
33	$HexNAc_5Hex_5Fuc_1NeuAc_1$	NQ	-
34	$HexNAc_4Hex_5NeuAc_2Ac$	NQ	1.30 ± 0.71
35	$HexNAc_5Hex_5Fuc_1NeuAc_2$	NQ	-
36	$HexNAc_5Hex_6NeuGc_2$	NQ	0.51 ± 0.14
37	$HexNAc_5Hex_6Fuc_2NeuAc_2$	NQ	-
38	$HexNAc_5Hex_6NeuAc_4$	NQ	-
39	$HexNAc_6Hex_5Fuc_1NeuAc_2$	NQ	-

[a] Errors correspond to one standard deviation.
[b] NQ ≡ detected but not quantified.

Table S13. Affinities (K_d, μM) measured by COIN-CaR-nMS screening of *N*-glycan (native and all α2-3-linked) natural libraries and 3SL (**G22**) and 3SLN (**G25**) against MAA. Measurements were performed on ammonium acetate solutions (200 mM, pH 6.9). [a,b]

Order #	Glycan composition	K_d
1	$HexNAc_5Hex_6NeuAc_3$	0.35 ± 0.11
2	$HexNAc_4Hex_5Fuc_1NeuAc_2$	0.39 ± 0.08
3	$HexNAc_5Hex_6Fuc_3NeuAc_1$	0.40 ± 0.14
4	$HexNAc_5Hex_5NeuAc_2$	0.44 ± 0.20
5	$HexNAc_4Hex_5NeuAc_1NeuGc_1$	0.48 ± 0.17
6	$HexNAc_5Hex_6Fuc_1NeuAc_3$	0.48 ± 0.15
7	$HexNAc_4Hex_5NeuGc_2$	0.49 ± 0.20
8	$HexNAc_4Hex_5NeuAc_2$	0.52 ± 0.20
9	$HexNAc_4Hex_5Fuc_1NeuAc_1$	0.57 ± 0.16
10	$HexNAc_5Hex_4Fuc_1NeuAc_1$	0.58 ± 0.14
11	$HexNAc_6Hex_7Fuc_1NeuAc_2$	0.58 ± 0.07
12	$HexNAc_5Hex_6Fuc_1NeuAc_2$	0.66 ± 0.17
13	$HexNAc_5Hex_6NeuAc_1$	0.67 ± 0.07
14	$HexNAc_4Hex_4Fuc_1NeuAc_1$	0.73 ± 0.24
15	$HexNAc_4Hex_5NeuAc_2Ac$	0.80 ± 0.35
16	$HexNAc_5Hex_4Fuc_1NeuAc_1S$	0.90 ± 0.20
17	$HexNAc_5Hex_4Fuc_1NeuAc_1$	0.99 ± 0.36
18	$HexNAc_5Hex_6NeuAc_2$	1.06 ± 0.04
19	$HexNAc_4Hex_5NeuGc_1$	1.10 ± 0.70
20	$HexNAc_4Hex_5Fuc_2NeuAc_1$	1.17 ± 0.40
21	$HexNAc_4Hex_5NeuAc_1$	1.76 ± 0.69
22	α-D-Neu5Ac-(2→3)-β-D-Gal-(1→4)-D-GlcNAc	2.14 ± 0.46
23	α-D-Neu5Ac-(2→3)-β-D-Gal-(1→4)-D-Glc	3.50 ± 1.50
24	$HexNAc_4Hex_5Fuc_1NeuGc_2$	NQ
25	$HexNAc_6Hex_5Fuc_1NeuAc_2$	NQ
26	$HexNAc_5Hex_6NeuAc_1NeuGc_1$	NQ
27	$HexNAc_5Hex_6NeuGc_2$	NQ
28	$HexNAc_5Hex_6NeuAc_4$	NQ

[a] Errors correspond to one standard deviation.
[b] NQ ≡ detected but not quantified.

Table S14. Affinities (K_d, μM) measured by COIN-CaR-nMS screening of *N*-glycan natural libraries against AAL. Measurements were performed on ammonium acetate solutions (200 mM, pH 6.9). [a,b]

**Order #**	**Glycan composition**	$\boldsymbol{K_d}$
1	$HexNAc_5Hex_6Fuc_1NeuAc_1$	0.27 ± 0.13
2	$HexNAc_4Hex_5Fuc_2NeuAc_1$	0.46 ± 0.20
3	$HexNAc_4Hex_5Fuc_3NeuAc_1$	0.50 ± 0.05
4	$HexNAc_4Hex_5Fuc_2NeuAc_1$	0.57 ± 0.10
5	$HexNAc_6Hex_3Fuc_1S$	0.60 ± 0.14
6	$HexNAc_5Hex_4Fuc_2NeuAc_1$	0.60 ± 0.09
7	$HexNAc_5Hex_5Fuc_1NeuAc_1$	0.61 ± 0.01
8	$HexNAc_4Hex_5Fuc_1NeuGc_2$	0.64 ± 0.34
9	$HexNAc_4Hex_4Fuc_1NeuAc_1$	0.65 ± 0.07
10	$HexNAc_3Hex_7Fuc_1$	0.70 ± 0.20
11	$HexNAc_4Hex_6Fuc_2$	0.70 ± 0.10
12	$HexNAc_5Hex_4Fuc_1NeuAc_1$	0.72 ± 0.30
13	$HexNAc_6Hex_3Fuc_1S_2$	0.90 ± 0.14
14	$HexNAc_5Hex_6Fuc_1NeuAc_2$	1.00 ± 0.30
15	$HexNAc_4Hex_5Fuc_1NeuAc_1NeuGc_1$	1.10 ± 0.07
16	$HexNAc_4Hex_6Fuc_1NeuAc_1$	1.30 ± 0.40
17	$HexNAc_4Hex_5Fuc_3$	1.34 ± 0.38
18	$HexNAc_3Hex_4Fuc_1NeuAc_1$	1.40 ± 0.40
19	$HexNAc_5Hex_4Fuc_1NeuAc_1S$	1.30 ± 0.56
20	$HexNAc_5Hex_3Fuc_1NeuAc_1$	1.50 ± 0.10
21	$HexNAc_4Hex_5Fuc_1NeuAc_2$	1.64 ± 0.40
22	$HexNAc_5Hex_4Fuc_1NeuAc_1$	1.80 ± 0.10
23	$HexNAc_5Hex_5Fuc_1NeuAc_1$	1.83 ± 0.76
24	$HexNAc_3Hex_3Fuc_1$	1.85 ± 0.07
25	$HexNAc_3Hex_5Fuc_1NeuAc_1$	1.85 ± 0.35
26	$HexNAc_4Hex_4Fuc_2$	2.20 ± 0.14
27	$HexNAc_3Hex_4Fuc_1NeuAc_1$	2.30 ± 0.57
28	$HexNAc_5Hex_5Fuc_1NeuAc_2$	2.50 ± 0.64
29	$HexNAc_4Hex_3Fuc_1$	2.60 ± 0.57
30	$HexNAc_5Hex_4Fuc_2S_1$	2.80 ± 1.30
31	$HexNAc_5Hex_6Fuc_2NeuAc_2$	3.40 ± 0.85
32	$HexNAc_3Hex_3Fuc_1NeuAc_1$	NQ
33	$HexNAc_5Hex_6Fuc_3$	NQ
34	$HexNAc_5Hex_6Fuc_2NeuAc_1$	NQ
35	$HexNAc_5Hex_6Fuc_4$	NQ
36	$HexNAc_5Hex_6Fuc_1NeuAc_3$	NQ
37	$HexNAc_6Hex_5Fuc_1NeuAc_3$	NQ
38	$HexNAc_4Hex_5Fuc_2NeuAc_2$	NQ
39	$HexNAc_6Hex_5Fuc_1NeuAc_2$	NQ
40	$HexNAc_4Hex_4Fuc_1$	NQ

41	$HexNAc_5Hex_3Fuc_1S_1$	NQ
42	$HexNAc_4Hex_5Fuc_1$	NQ
43	$HexNAc_4Hex_5Fuc_2$	NQ
44	$HexNAc_6Hex_3Fuc_1NeuAc_1$	NQ
45	$HexNAc_6Hex_3Fuc_2NeuAc_1$	NQ

[a] Errors correspond to one standard deviation.

[b] NQ ≡ detected but not quantified.

Table S15. Affinities (K_d, μM) measured by COIN-CaR-nMS screening of *N*-glycan (native and all α2-6- and all α2-3-linked) natural libraries against RCA-I. Measurements were performed on ammonium acetate solutions (200 mM, pH 6.9). [a,b]

Order #	Glycan composition	K_d (Native)	K_d (α2-6-NeuAc)	K_d (α2-3-NeuAc)
1	$HexNAc_6Hex_7Fuc_1NeuAc_2$	0.26 ± 0.11		
2	$HexNAc_5Hex_6Fuc_2$	0.26 ± 0.18		
3	$HexNAc_5Hex_5Fuc_2$	0.31 ± 0.09		
4	$HexNAc_5Hex_6Fuc_2$	0.32 ± 0.16		
5	$HexNAc_5Hex_6NeuAc_2$ $NeuGc_1$	0.32 ± 0.10		
6	$HexNAc_5Hex_6NeuAc_3$	0.39 ± 0.17		
7	$HexNAc_5Hex_6Fuc_1$	0.39 ± 0.10		
8	$HexNAc_5Hex_6NeuAc_2$	0.42 ± 0.06		
9	$HexNAc_5Hex_6Fuc_1NeuAc_1$	0.49 ± 0.08		
10	$HexNAc_4Hex_5Fuc_1NeuAc_1NeuGc_1$	0.49 ± 0.09		
11	$HexNAc_5Hex_6Fuc_2NeuAc_1$	0.49 ± 0.09		
12	$HexNAc_4Hex_6Fuc_1NeuAc_1$	0.50 ± 0.08		
13	$HexNAc_4Hex_5Fuc_1NeuGc_2$	0.50 ± 0.09		
14	$HexNAc_5Hex_6NeuGc_2$	0.51 ± 0.17		
15	$HexNAc_5Hex_4Fuc_2NeuAc_1$	0.53 ± 0.21		
16	$HexNAc_5Hex_6Fuc_1NeuAc_2$	0.56 ± 0.15		
17	$HexNAc_4Hex_6NeuGc_1$	0.56 ± 0.17		
18	$HexNAc_5Hex_4Fuc_1$	0.57 ± 0.20		
19	$HexNAc_4Hex_5Fuc_1NeuGc_1$	0.57 ± 0.18		
20	$HexNAc_5Hex_5NeuAc_2$	0.58 ± 0.10		
21	$HexNAc_5Hex_6NeuAc_1NeuGc_1$	0.58 ± 0.09		
22	$HexNAc_4Hex_4Fuc_3$	0.64 ± 0.20		
23	$HexNAc_4Hex_5NeuAc_1$	0.64 ± 0.04		
24	$HexNAc_5Hex_4Fuc_1NeuAc_1S$	0.70 ± 0.04		
25	$HexNAc_4Hex_5NeuGc_1$	0.74 ± 0.07		
26	$HexNAc_4Hex_5Fuc_2NeuAc_1$	0.75 ± 0.15		
27	$HexNAc_3Hex_4Fuc_2$	0.77 ± 0.19		
28	$HexNAc_4Hex_5Fuc_3NeuAc_1$	0.84 ± 0.04		
29	$HexNAc_5Hex_4Fuc_2NeuAc_1$	0.84 ± 0.18		
30	$HexNAc_4Hex_5Fuc_3$	0.84 ± 0.20		
31	$HexNAc_5Hex_6Fuc_2NeuAc_2$	0.90 ± 0.08		
32	$HexNAc_5Hex_6NeuGc_1$	0.90 ± 0.10		
33	$HexNAc_4Hex_4Fuc_1NeuAc_1$	0.91 ± 0.26		
34	$HexNAc_4Hex_5NeuGc_2$	0.95 ± 0.15		
35	$HexNAc_6Hex_5Fuc_1NeuAc_2$	0.96 ± 0.01		
36	$HexNAc_5Hex_6NeuAc_1$	1.05 ± 0.01	0.90 ± 0.23	0.80 ± 0.29
37	$HexNAc_4Hex_5NeuAc_2$	1.09 ± 0.16	1.10 ± 0.01	2.16 ± 0.01
38	$HexNAc_4Hex_5NeuAc_1NeuGc_1$	1.10 ± 0.29		
39	$HexNAc_5Hex_5Fuc_1NeuAc_1$	1.13 ± 0.44		

40	$HexNAc_5Hex_6Fuc_2NeuAc_1$	1.17 ± 0.01		
41	$HexNAc_3Hex_5Fuc_1$	1.17 ± 0.33		
42	$HexNAc_4Hex_5Fuc_1NeuAc_1$	1.20 ± 0.27		
43	$HexNAc_4Hex_5$	1.22 ± 0.03		
44	$HexNAc_5Hex_5NeuAc_1$	1.24 ± 0.23		
45	$HexNAc_5Hex_4Fuc_1NeuAc_1$	1.28 ± 0.42		
46	$HexNAc_4Hex_4Fuc_1$	1.30 ± 0.45		
47	$HexNAc_6Hex_3Fuc_1NeuAc_1$	1.30 ± 0.09		
48	$HexNAc_4Hex_4NeuAc_1$	1.35 ± 0.33		
49	$HexNAc_4Hex_5Fuc_1$	1.40 ± 0.40		
50	$HexNAc_5Hex_4Fuc_1S$	1.43 ± 0.08		
51	$HexNAc_4Hex_5Fuc_2NeuAc_2$	1.50 ± 0.03		
52	$HexNAc_4Hex_5Fuc_2$	1.63 ± 0.28		
53	$HexNAc_4Hex_3Fuc_1$	1.73 ± 0.08		
54	$HexNAc_4Hex_5Fuc_1NeuAc_2$	1.74 ± 0.60		
55	$HexNAc_5Hex_4Fuc_1NeuAc_2$	1.97 ± 0.90		
56	$HexNAc_4Hex_4$	2.10 ± 0.70		
57	$HexNAc_5Hex_5Fuc_3$	2.30 ± 0.32		
58	$HexNAc_4Hex_4Fuc_2$	2.40 ± 0.50		
59	$HexNAc_4Hex_3Fuc_2$	2.70 ± 0.44		
60	$HexNAc_5Hex_4Fuc_3$	2.70 ± 0.80		
61	$HexNAc_5Hex_3Fuc_2$	4.40 ± 0.14		
62	$HexNAc_5Hex_3Fuc_1NeuAc_1$	NQ		

[a] Errors correspond to one standard deviation.

[b] NQ ≡ detected but not quantified.

Table S16. Affinities (K_d, μM) measured by COIN-CaR-nMS screening of *N*-glycan (native and all α2-6- and all α2-3-linked) natural libraries against PHA-E. Measurements were performed on ammonium acetate solutions (200 mM, pH 6.9). [a,b]

Order #	Glycan composition	K_d (Native)	K_d (α2-6-NeuAc)	K_d (α2-3-NeuAc)
1	$HexNAc_5Hex_5Fuc_1NeuAc_1$	9.3 ± 2.1		
2	$HexNAc_4Hex_5NeuAc_1$	10.3 ± 1.1	20.6 ± 0.8	11.9 ± 4.3
3	$HexNAc_4Hex_5Fuc_1NeuGc_1$	11.1 ± 0.6		
4	$HexNAc_4Hex_5Fuc_2NeuAc_1$	11.2 ± 1.8		
5	$HexNAc_5Hex_4Fuc_1NeuAc_1$	11.3 ± 1.3		
6	$HexNAc_4Hex_5NeuAc_2$	11.4 ± 0.9	31.6 ± 4.9	14.5 ± 0.5
7	$HexNAc_5Hex_5NeuAc_2$	11.4 ± 1.6		
8	$HexNAc_4Hex_5NeuGc_1$	12.1 ± 0.6		
9	$HexNAc_4Hex_5Fuc_1$	12.3 ± 3.6		
10	$HexNAc_4Hex_5$	13.1 ± 0.8		
11	$HexNAc_4Hex_4Fuc_2$	13.7 ± 1.2		
12	$HexNAc_5Hex_4Fuc_2NeuAc_1$	14.1 ± 1.1		
13	$HexNAc_4Hex_5Fuc_1NeuAc_2$	15.1 ± 0.5		
14	$HexNAc_4Hex_5NeuAc_1NeuGc_1$	15.9 ± 2.7		
15	$HexNAc_4Hex_5Fuc_1NeuAc_1NeuGc_1$	16.0 ± 0.6		
16	$HexNAc_4Hex_5Fuc_1NeuAc_1$	16.0 ± 0.4		
17	$HexNAc_5Hex_6NeuAc_1$	16.1 ± 2.3	26.4 ± 4.0	15.5 ± 0.5
18	$HexNAc_4Hex_5Fuc_1NeuGc_2$	17.8 ± 1.8		
19	$HexNAc_5Hex_6NeuAc_3$	18.5 ± 3.3	-	17.8 ± 0.4
20	$HexNAc_4Hex_3Fuc_1$	18.6 ± 3.5		
21	$HexNAc_4Hex_5NeuGc_2$	19.9 ± 0.1		
22	$HexNAc_5Hex_6NeuGc_1$	19.9 ± 6.8		
23	$HexNAc_5Hex_6NeuGc_2$	22.8 ± 4.5		
24	$HexNAc_5Hex_6NeuAc_2$	23.6 ± 1.3	36.8 ± 4.1	22.0 ± 2.2
25	$HexNAc_5Hex_6Fuc_1NeuAc_2$	25.3 ± 1.6		
26	$HexNAc_5Hex_6NeuGc_1NeuAc_1$	26.1 ± 0.9		
27	$HexNAc_4Hex_4Fuc_1NeuAc_1$	32.9 ± 2.4		
28	$HexNAc_5Hex_4Fuc_2NeuAc_1$	NQ		

[a] Errors correspond to one standard deviation.
[b] NQ ≡ detected but not quantified.

Table S17. Affinities (K_d, μM) measured by COIN-CaR-nMS screening of *N*-glycan (native and all α2-6- and all α2-3-linked) natural libraries and **G21** against CD22-Fc. Measurements were performed on ammonium acetate solutions (200 mM, pH 6.9). [a,b]

Order #	Glycan composition	K_d
1	$HexNAc_5Hex_6NeuAc_3$	21.5 ± 1.7
2	$HexNAc_4Hex_5NeuAc_2$	23.5 ± 1.4
3	$HexNAc_5Hex_6NeuAc_2$	23.6 ± 6.3
4	$HexNAc_4Hex_5Fuc_1NeuAc_2$	25.4 ± 3.6
5	$HexNAc_4Hex_5NeuAc_1NeuGc_1$	25.8 ± 10.0
6	$HexNAc_4Hex_5NeuAc_2Ac$	26.0 ± 3.0
7	$HexNAc_4Hex_5NeuGc_2$	26.0 ± 9.2
8	$HexNAc_4Hex_4Fuc_1NeuAc_1$	30.3 ± 4.1
9	$HexNAc_4Hex_5NeuGc_1$	30.6 ± 10.0
10	$HexNAc_5Hex_4Fuc_1NeuAc_1$	31.0 ± 6.1
11	$HexNAc_4Hex_5Fuc_2NeuAc_1$	31.3 ± 4.1
12	$HexNAc_4Hex_5NeuAc_1$	34.5 ± 6.3
13	$HexNAc_4Hex_5Fuc_1NeuAc_1$	35.2 ± 5.9
14	α-D-Neu5Ac-(2→6)-β-D-Gal-(1→4)-D-Glc	40.0 ± 1.0

[a] Errors correspond to one standard deviation.

[b] NQ ≡ detected but not quantified.

Table S18. Affinities (K_d, µM) measured by COIN-CaR-nMS screening of *N*-glycan (native and all α2-6- and all α2-3-linked) natural libraries and purified oligosaccharide against Sig7-Fc. Measurements were performed on ammonium acetate solutions (200 mM, pH 6.9). [a,b]

Order #	Glycan composition	K_d
1	α-D-Neu5Ac-(2→8)-α-D-Neu5Ac-(2→3)-β-D-Gal-(1→4)-D-Glc (GD3)	97 ± 14
2	α-D-Neu5Ac-(2→8)-α-D-Neu5Ac-(2→8)-α-D-Neu5Ac-(2→3)-β-D-Gal-(1→4)-D-Glc (GT3)	184 ± 41
3	α-D-GalNAc(1→4)-[α-D-Neu5Ac-(2→8)-α-D-Neu5Ac-(2→3)]-β-D-Gal-(1→4)-D-Glc (GD2)	205 ± 41
4	$HexNAc_4Hex_5NeuGc_1$	220 ± 39
5	$HexNAc_4Hex_5NeuAc_1NeuGc_1$	222 ± 43
6	α-D-GalNAc(1→4)-[α-D-Neu5Ac-(2→8)-α-D-Neu5Ac-(2→8)-α-D-Neu5Ac-(2→3)]-β-D-Gal-(1→4)-D-Glc (GT2)	235 ± 20
7	α-D-Neu5Ac-(2→6)-β-D-Gal-(1→4)-D-Glc	236 ± 69
8	$HexNAc_4Hex_5NeuAc_1$	260 ± 76
9	α-D-Neu5Ac-(2→6)-β-D-Gal-(1→4)-D-GlcNAc	265 ± 66
10	α-D-Neu5Ac-(2→3)-β-D-Gal-(1→4)-D-GlcNAc	267 ± 26
11	α-D-Neu5Ac-(2→3)-β-D-Gal-(1→4)-D-Glc	277 ± 50
12	$HexNAc_4Hex_5NeuAc_2$	312 ± 67
13	$HexNAc_4Hex_5NeuGc_2$	354 ± 99
14	β-D-Gal(1→3)-α-D-GalNAc(1→4)-[α-D-Neu5Ac-(2→3)]-β-D-Gal-(1→4)-D-Glc (GM1)	437 ± 56
15	α-D-GalNAc(1→4)-[α-D-Neu5Ac-(2→3)]-β-D-Gal-(1→4)-D-Glc (GM2)	460 ± 117
16	β-D-Gal(1→3)-α-D-GalNAc(1→4)-[α-D-Neu5Ac-(2→8)-α-D-Neu5Ac-(2→3)]-β-D-Gal-(1→4)-D-Glc (GD1b)	1217 ± 401
17	β-D-Gal(1→3)-α-D-GalNAc(1→4)-[α-D-Neu5Ac-(2→8)-α-D-Neu5Ac-(2→8)-α-D-Neu5Ac-(2→3)]-β-D-Gal-(1→4)-D-Glc (GT1c)	NQ

[a] Errors correspond to one standard deviation.
[b] NQ ≡ detected but not quantified.

Table S19. Affinities (K_d, μM) measured by COIN-CaR-nMS screening of *N*-glycan (native and all α2-6- and all α2-3-linked) natural libraries and **G22** against SARS-CoV-2 RBD. Measurements were performed on ammonium acetate solutions (200 mM, pH 6.9). [a,b]

Order #	Glycan composition	K_d
1	$HexNAc_4Hex_5NeuAc_1$	52 ± 1
2	$HexNAc_3Hex_4Fuc_1NeuAc_1$	57 ± 5
3	$HexNAc_4Hex_4Fuc_1NeuAc_1$	68 ± 14
4	$HexNAc_4Hex_5Fuc_2NeuAc_1$	69 ± 21
5	$HexNAc_5Hex_3Fuc_1NeuAc_1$	72 ± 1
6	$HexNAc_3Hex_5Fuc_1NeuAc_1$	73 ± 20
7	α-D-Neu5Ac-(2→6)-β-D-Gal-(1→4)-D-GlcNAc	76 ± 9
8	$HexNAc_4Hex_5NeuAc_1NeuGc_1$	78 ± 15
9	$HexNAc_4Hex_5NeuGc_1$	79 ± 2
10	$HexNAc_3Hex_5NeuAc_1$	80 ± 1
11	$HexNAc_5Hex_4Fuc_1NeuAc_2$	82 ± 19
12	$HexNAc_4Hex_4NeuAc_1$	84 ± 2
13	$HexNAc_4Hex_5Fuc_1NeuGc_2$	98 ± 8
14	$HexNAc_4Hex_5NeuAc_1$	102 ± 33
15	$HexNAc_4Hex_5Fuc_1NeuAc_2S$	103 ± 6
16	$HexNAc_5Hex_5Fuc_1NeuAc_1$	104 ± 6
17	$HexNAc_5Hex_6Fuc_1NeuAc_1$	105 ± 33
18	$HexNAc_5Hex_5Fuc_1NeuAc_2$	108 ± 1
19	$HexNAc_5Hex_6NeuGc_1$	109 ± 11
20	$HexNAc_4Hex_6NeuGc_1$	113 ± 4
21	$HexNAc_4Hex_5Fuc_1NeuGc_1$	116 ± 10
22	$HexNAc_4Hex_5NeuAc_2$	116 ± 18
23	$HexNAc_4Hex_5NeuGc_2$	117± 10
24	$HexNAc_4Hex_5NeuAc_2Ac$	119 ± 8
25	$HexNAc_5Hex_6NeuAc_1NeuGc_1$	120 ± 26
26	$HexNAc_4Hex_5Fuc_1NeuAc_1NeuGc_1$	138 ± 6
27	$HexNAc_5Hex_6NeuGc_2$	140 ± 21
28	$HexNAc_5Hex_6Fuc_2NeuAc_2$	141 ± 1
29	$HexNAc_5Hex_6NeuAc_3$	151 ± 40
30	$HexNAc_4Hex_5Fuc_1NeuAc_1$	153 ± 15
31	$HexNAc_4Hex_5Fuc_1NeuAc_2$	159 ± 1
32	$HexNAc_5Hex_6NeuAc_1$	162 ± 4
33	$HexNAc_5Hex_6NeuAc_2$	202 ± 1
34	$HexNAc_5Hex_6Fuc_1NeuAc_2$	208 ± 4
35	$HexNAc_5Hex_4Fuc_1NeuAc_1$	233 ± 45
36	$HexNAc_3Hex_4NeuAc_1$	NQ

[a] Errors correspond to one standard deviation.

[b] NQ ≡ detected but not quantified.

Figures

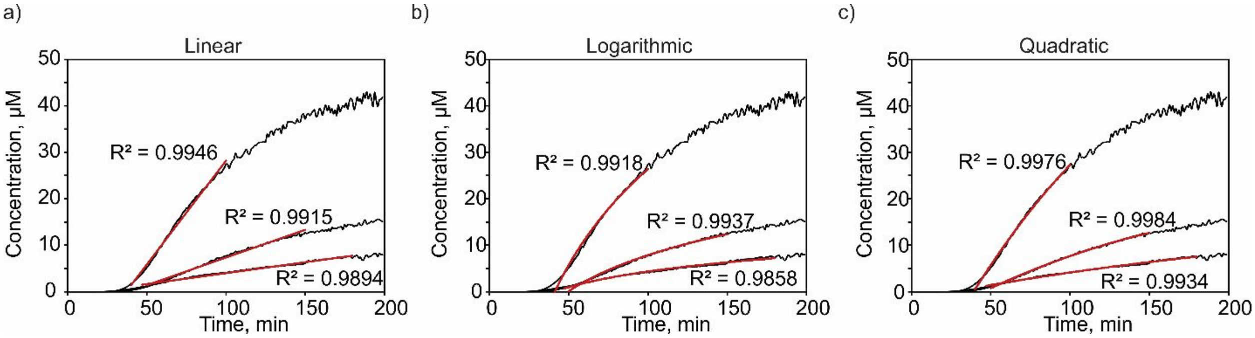

Figure S1. Time-dependent concentration changes of **G23** in the presence of GBP (GAL-3C) with concentration in Solution 2 of 20, 50 and 100 μM. Red curves are the best fit for the region of data after mixing onset using (a) linear ($y = ax+b$), (b) logarithmic ($y = a\ln(x)+b$) and (c) quadratic models ($y = ax^2 + bx+c$).

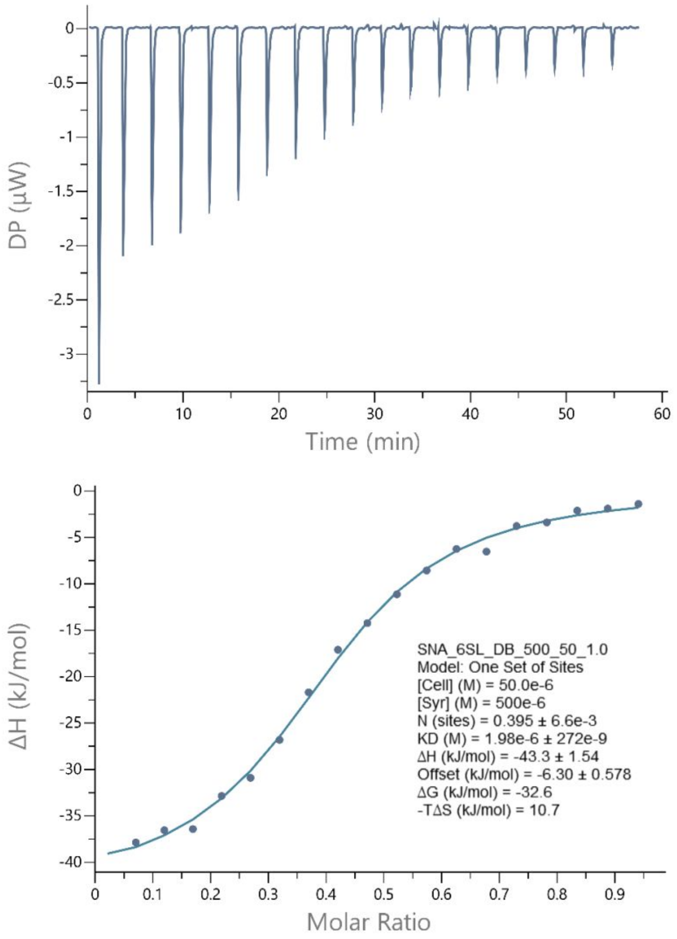

Figure S2. ITC data measured for the binding of SNA (50 μM) to **G21** (0.5 mM) in aqueous ammonium acetate (200 mM, pH 6.9 and 25 °C).

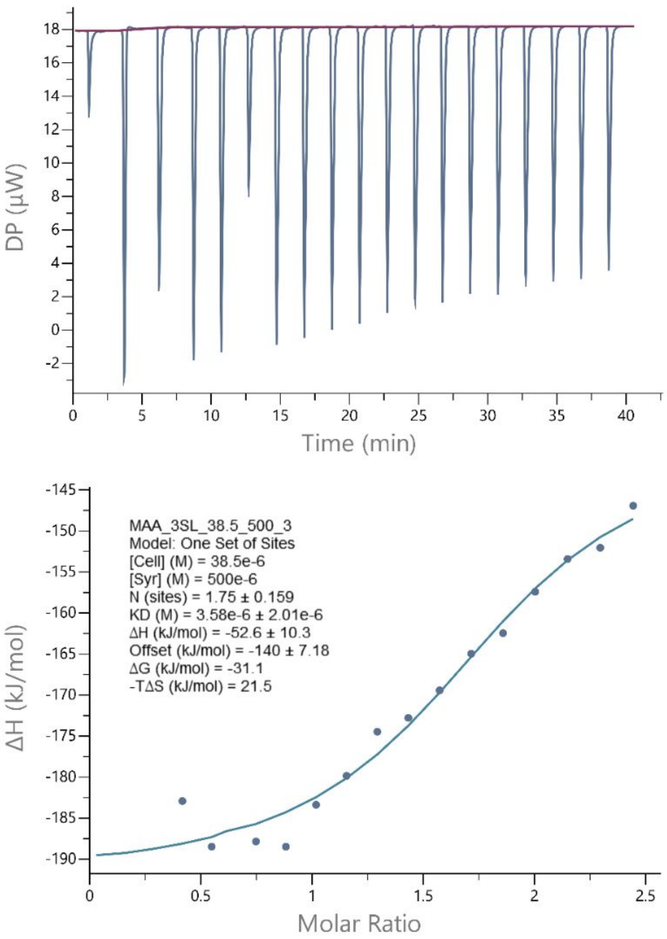

Figure S3. ITC data measured for the binding of MAA (38.5 μM) to **G23** (0.5 mM) in aqueous ammonium acetate (200 mM, pH 6.9 and 25 °C).

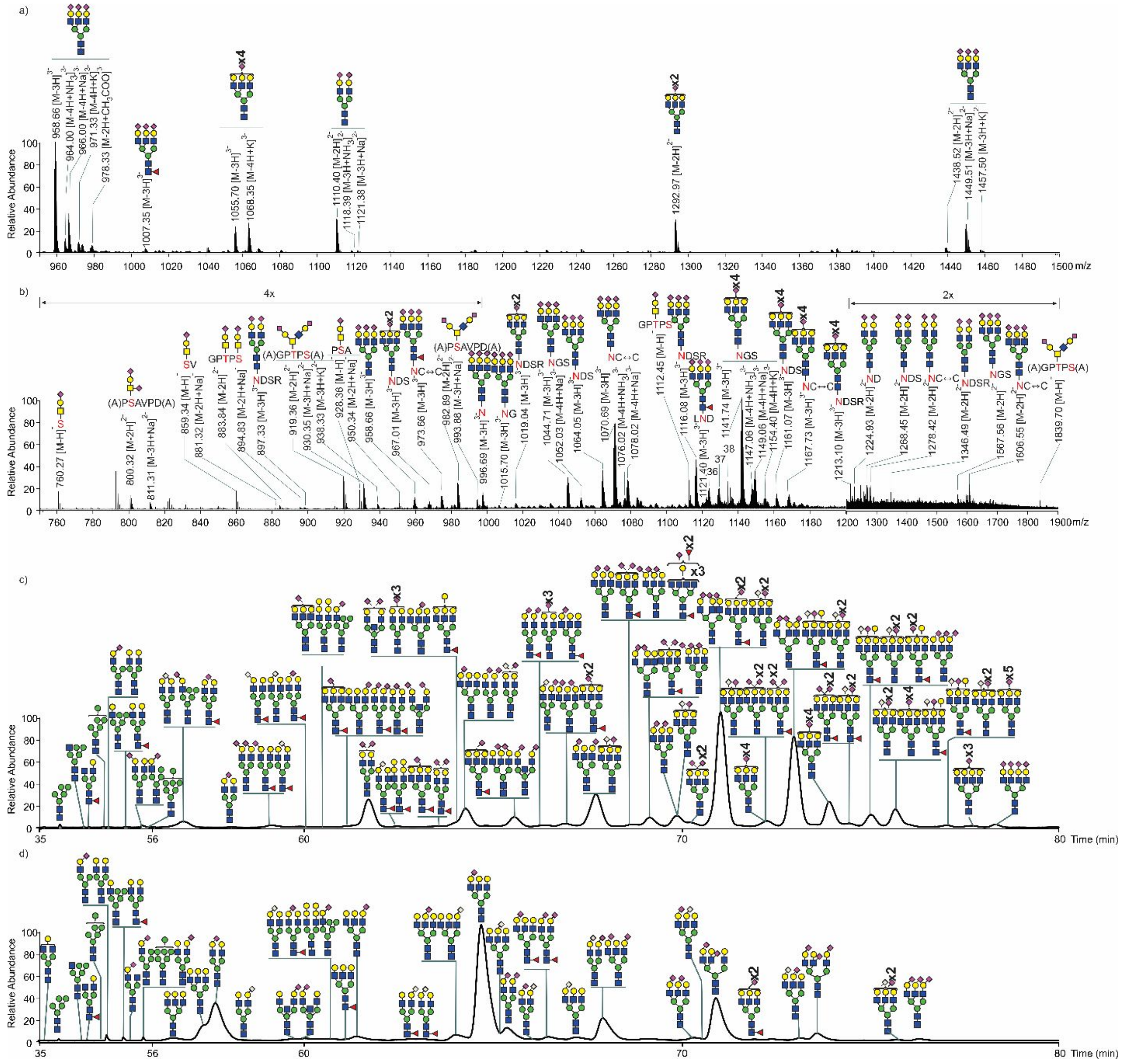

Figure S4. Glycan composition of bovine fetuin (BF). Representative ESI mass spectra of glycans and glycopeptides derived from BF using (a) PNGase F or (b) Pronase digestion in ammonium acetate (200 mM, pH 6.8) solutions. HILIC chromatogram of (c) BF glycans labeled with procainamide; (d) NeuS-treated BF glycans labeled with procainamide.

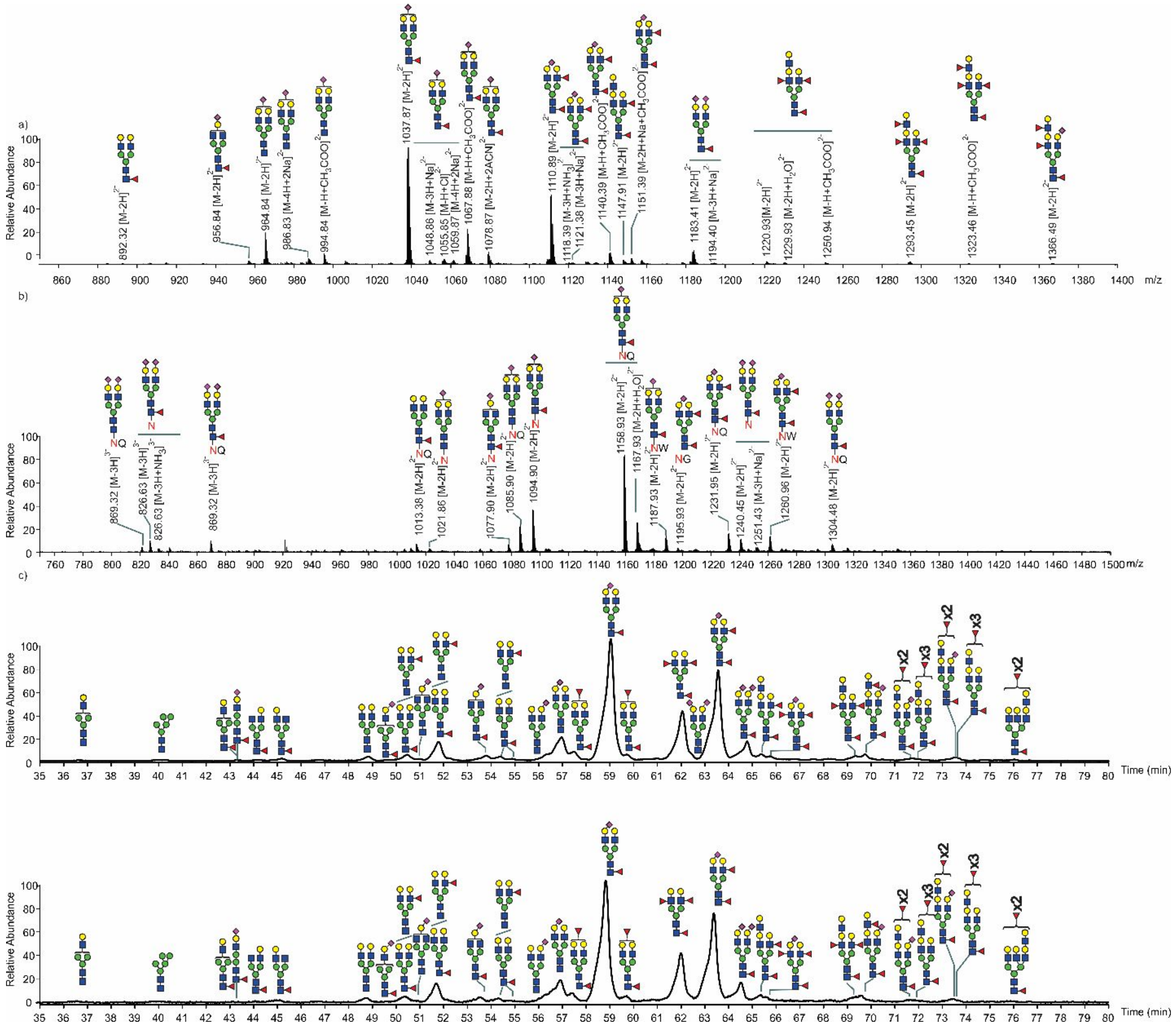

Figure S5. Glycan composition of human lactoferrin (HF). Representative ESI mass spectra of glycans and glycopeptides derived from HF using (a) PNGase F or (b) Pronase digestion in ammonium acetate (200 mM, pH 6.8) solutions. HILIC chromatogram of (c) HF glycans labeled with procainamide; (d) NeuS-treated HF glycans labeled with procainamide.

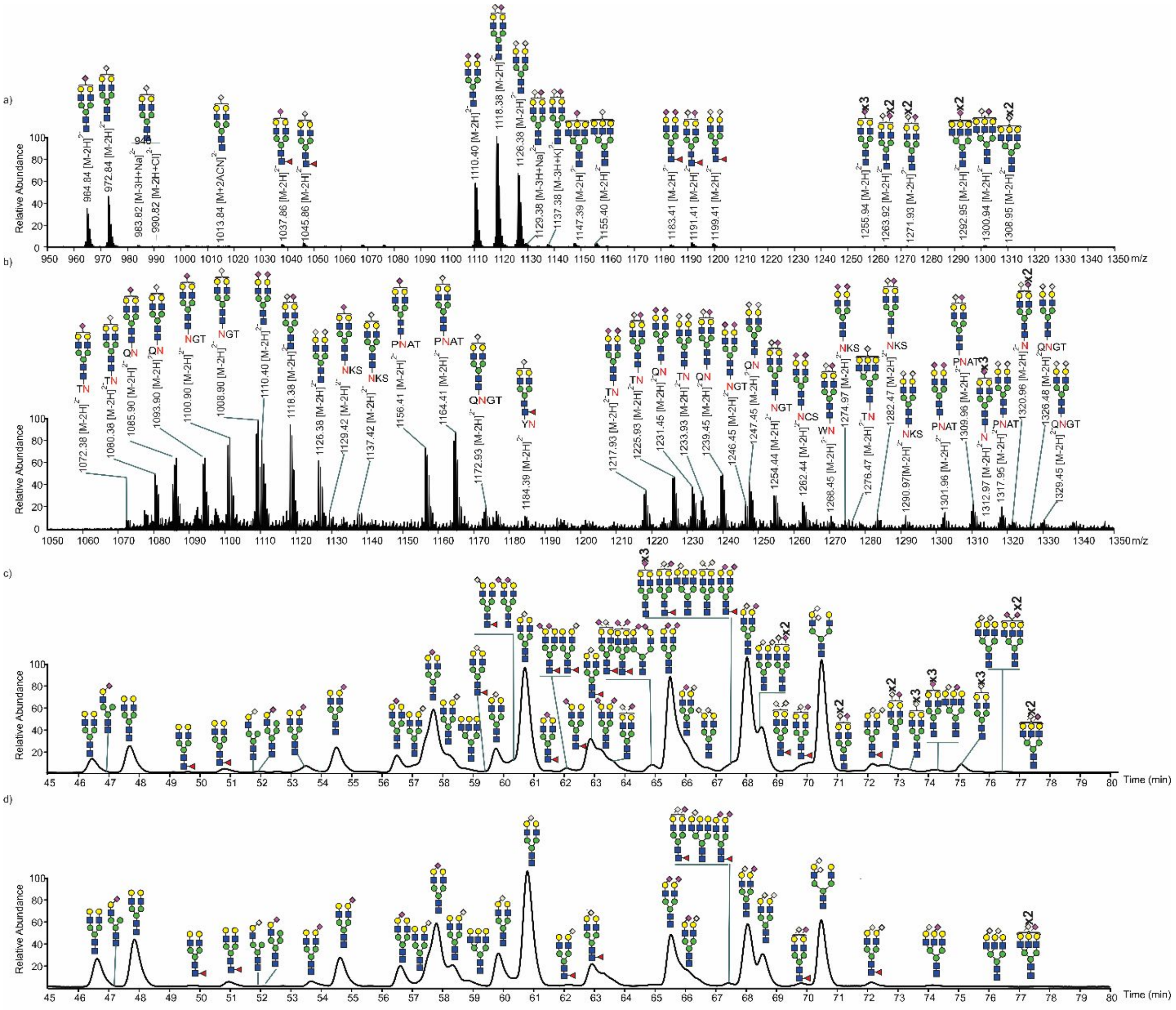

Figure S6. Glycan composition of bovine AGP (bAGP). Representative ESI mass spectra of glycans and glycopeptides derived from bAGP using (a) PNGase F or (b) Pronase digestion in ammonium acetate (200 mM, pH 6.8) solutions. HILIC chromatogram of (c) bAGP glycans labeled with procainamide; (d) NeuS-treated bAGP glycans labeled with procainamide.

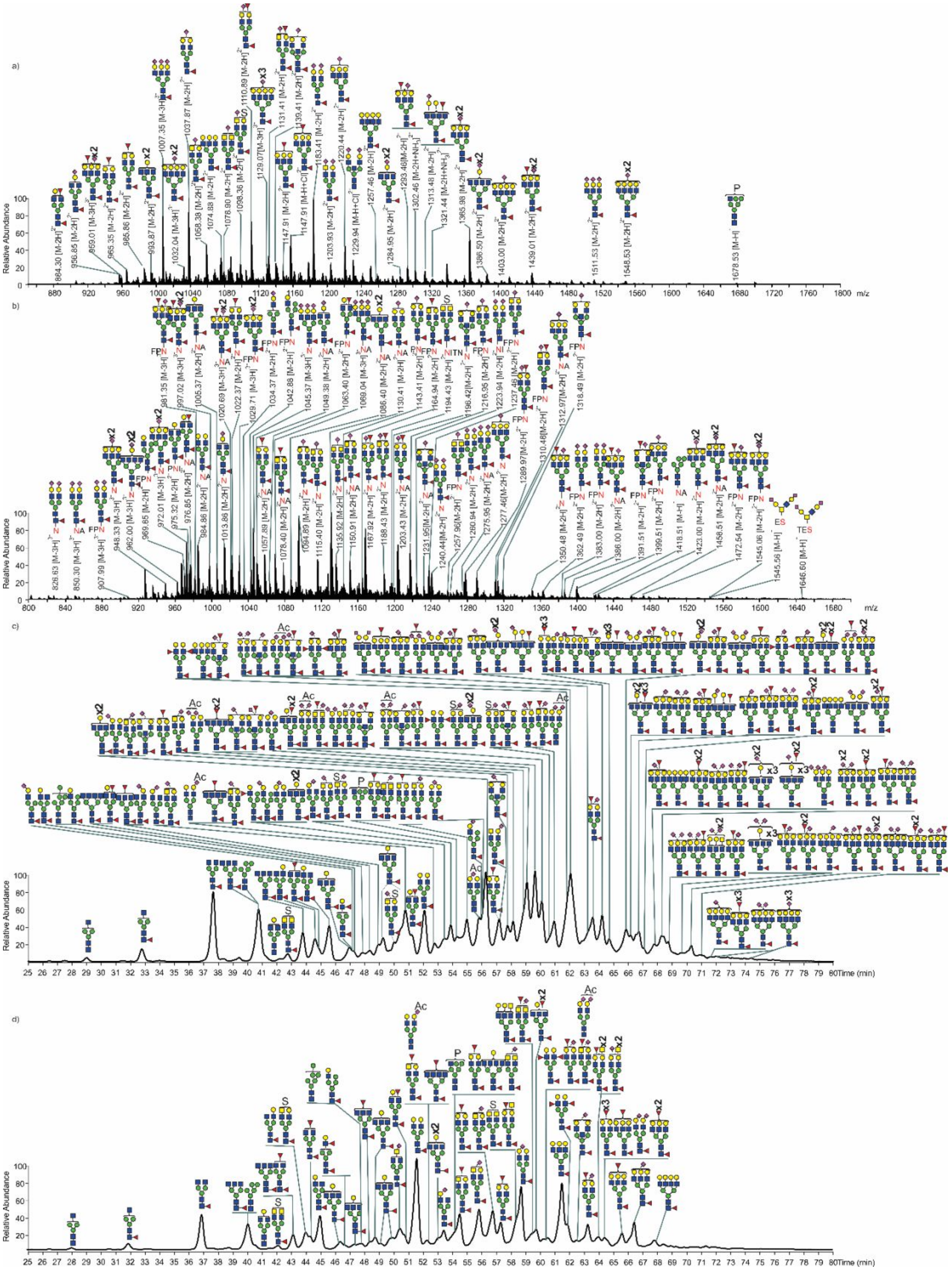

Figure S7. Glycan composition of SARS-CoV-2 RBD. Representative ESI mass spectra of glycans and glycopeptides derived from RBD using (a) PNGase F or (b) Pronase digestion in ammonium acetate (200 mM, pH 6.8) solutions. HILIC chromatogram of (c) RBD glycans labeled with procainamide; (d) NeuS-treated RBD glycans labeled with procainamide.

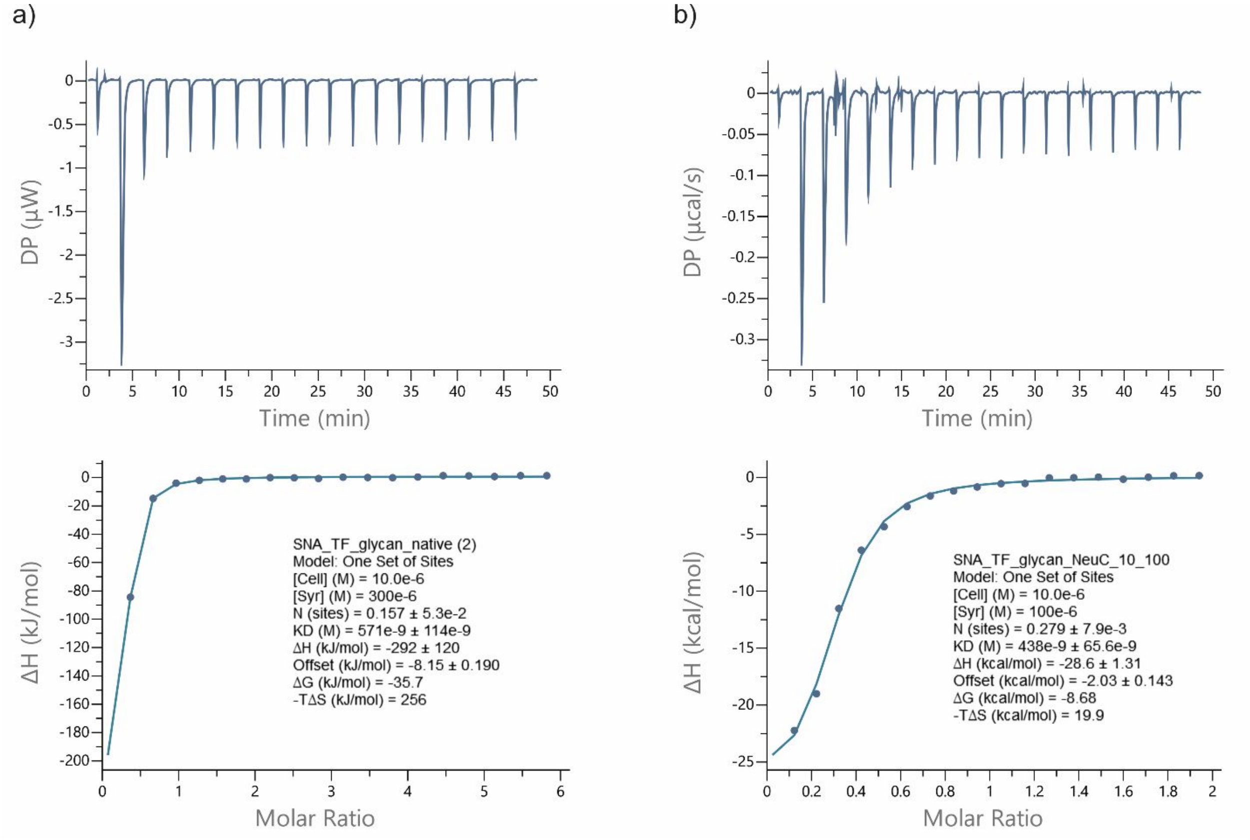

Figure S8. ITC data measured for SNA (10 μM) binding to (a) *N*-glycans from TF (0.3 mM) and (b) *N*-glycan from NeuC-treated TF (0.1 mM) in aqueous ammonium acetate (200 mM, pH 6.9 and 25 °C).

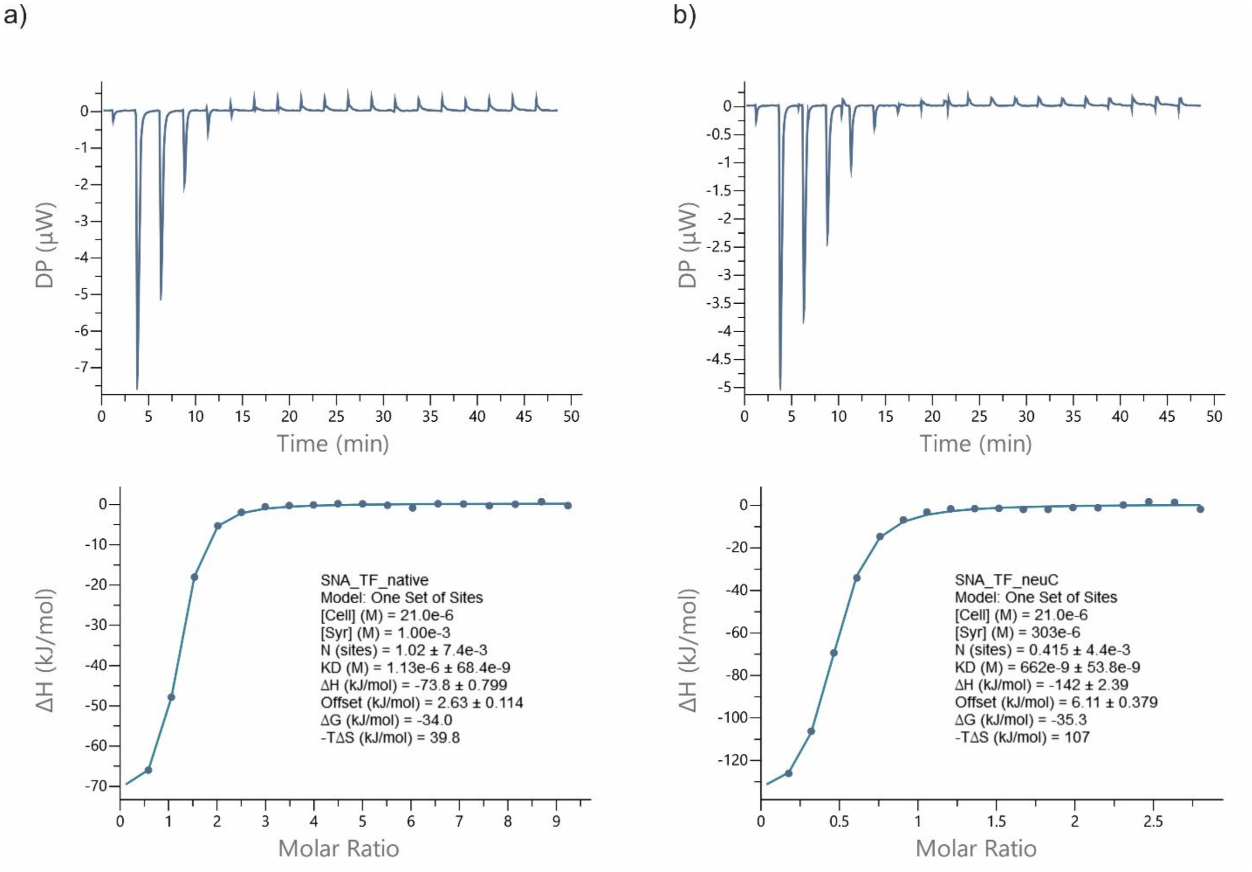

Figure S9. ITC data measured for SNA (21 μM) binding to (a) TF (0.5 mM) and (b) NeuC-treated TF (0.3 mM) in aqueous ammonium acetate (200 mM, pH 6.9 and 25 °C).

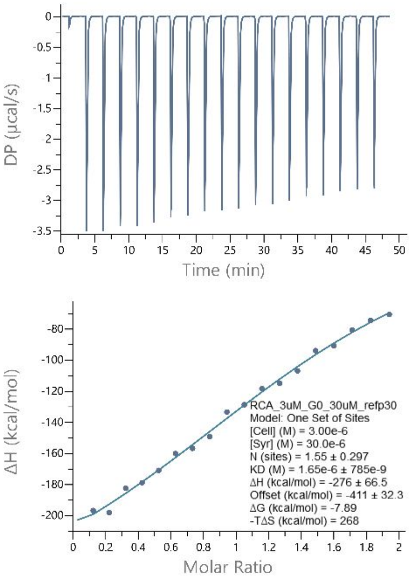

Figure S10. ITC data measured for RCA-I (3 μM) binding to **G34** (, 30 μM) in aqueous ammonium acetate (200 mM, pH 6.9 and 25 °C).

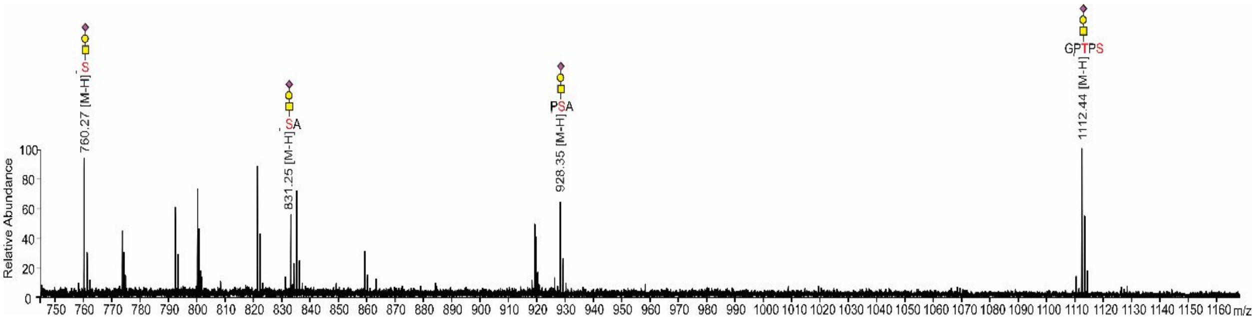

Figure S11. Representative mass spectrum measured by CaR-nMS (CE 90) performed on an ammonium acetate solution (200 mM, pH 6.8) of Sig7-Fc (2 μM) and a pronase digest of RBD and BF.

References

(S1) Moremen, K. W.; Ramiah, A.; Stuart, M.; Steel, J.; Meng, L.; Forouhar, F.; Moniz, H. A.; Gahlay, G.; Gao, Z.; Chapla, D.; Wang, S.; Yang, J.-Y.; Prabhakar, P. K.; Johnson, R.; Rosa, M. dela; Geisler, C.; Nairn, A. V; Seetharaman, J.; Wu, S.-C.; Tong, L.; Gilbert, H. J.; LaBaer, J.; Jarvis, D. L. Expression System for Structural and Functional Studies of Human Glycosylation Enzymes. *Nat. Chem. Biol.* **2018**, *14*, 156–162.

(S2) Li, Z.; Kitov, P. I.; Kitova, E. N.; Bui, D. T.; Moremen, K. W.; Wakarchuk, W. W.; Mahal, L. K.; Macauley, M. S.; Klassen, J. S. Quantifying Carbohydrate-Active Enzyme Activity with Glycoprotein Substrates Using Electrospray Ionization Mass Spectrometry and Center-of-Mass Monitoring. *Anal. Chem.* **2021**, *93*, 15262-15270.

(S3) Bui, D. T.; Jung, J.; Kitova, E. N.; Li, Z.; Willows, S. D.; Boddington, M. E.; Kitov, P. I.; Mason, A. L.; Capicciotti, C. J.; Mahal, L. K.; Macauley, M. S.; Klassen, J. S. Mass Spectrometry-Based Shotgun Glycomics Using Labeled Glycan Libraries. *Anal. Chem.* **2022**, *94*, 4997–5005.

(S4) Stanley, P.; Taniguchi, N.; Aebi, M. N-Glycans.; Varki, A., Cummings, R. D., Esko, J. D., Stanley, P., Hart, G. W., Aebi, M., Darvill, A. G., Kinoshita, T., Packer, N. H., Prestegard, J. H., Schnaar, R. L., Seeberger, P. H., Eds.; Cold Spring Harbor (NY), 2015; pp 99–111.

(S5) Walsh, I.; O'Flaherty, R.; Rudd, P. M. Bioinformatics Applications to Aid High-Throughput Glycan Profiling. *Perspect. Sci.* **2017**, *11*, 31–39.

(S6) Gut, H.; King, S. J.; Walsh, M. A. Structural and Functional Studies of Streptococcus Pneumoniae Neuraminidase B: An Intramolecular Trans-Sialidase. *FEBS Lett.* **2008**, *582*, 3348–3352.

(S7) Kitov, P. I.; Han, L.; Kitova, E. N.; Klassen, J. S. Sliding Window Adduct Removal Method (SWARM) for Enhanced Electrospray Ionization Mass Spectrometry Binding Data. *J. Am. Soc. Mass Spectrom.* **2019**, *30*, 1446–1454.

(S8) Nguyen, L.; McCord, K. A.; Bui, D. T.; Bouwman, K. M.; Kitova, E. N.; Elaish, M.; Kumawat, D.; Daskhan, G. C.; Tomris, I.; Han, L.; Chopra, P.; Yang, T.-J.; Willows, S. D.; Mason, A. L.; Mahal, L. K.; Lowary, T. L.; West, L. J.; Hsu, S.-T. D.; Hobman, T.; Tompkins, S. M.; Boons, G.-J.; de Vries, R. P.; Macauley, M. S.; Klassen, J. S. Sialic Acid-Containing Glycolipids Mediate Binding and Viral Entry of SARS-CoV-2. *Nat. Chem. Biol.* **2022**, *18*, 81-90.

(S9) Han, L.; Kitova, E. N.; Tan, M.; Jiang, X.; Pluvinage, B.; Boraston, A. B.; Klassen, J. S. Affinities of Human Histo-Blood Group Antigens for Norovirus Capsid Protein Complexes. *Glycobiology* **2015**, *25*, 170–180.

(S10) Shams-Ud-Doha, K.; Kitova, E. N.; Kitov, P. I.; St-Pierre, Y.; Klassen, J. S. Human Milk Oligosaccharide Specificities of Human Galectins. Comparison of Electrospray Ionization Mass Spectrometry and Glycan Microarray Screening Results. *Anal. Chem.* **2017**, *89*, 4914–4921.

(S11) Rodrigues, E.; Jung, J.; Park, H.; Loo, C.; Soukhtehzari, S.; Kitova, E. N.; Mozaneh, F.; Daskhan, G.; Schmidt, E. N.; Aghanya, V.; Sarkar, S.; Streith, L.; St. Laurent, C. D.; Nguyen, L.; Julien, J.-P.; West, L. J.; Williams, K. C.; Klassen, J. S.; Macauley, M. S. A Versatile Soluble Siglec Scaffold for Sensitive and Quantitative Detection of Glycan Ligands. *Nat. Commun.* **2020**, *11*, 5091.
