# Supplementary material for: Absolute Affinities from Quantitative Shotgun Glycomics Using Concentration-Independent (COIN) Native Mass Spectrometry
Source: ACS Cent Sci. 2023 Jun 15;9(7):1374–87. doi: 10.1021/acscentsci.3c00294 (PMC10303200; doi:10.1021/acscentsci.3c00294)
Supplement: Supplementary file 2 — oc3c00294_si_002.pdf [file oc3c00294_si_002.pdf]

Supporting Information File 2 for:

Absolute Affinities from Quantitative Shotgun Glycomics Using Concentration-Independent (COIN) Native Mass Spectrometry

Duong T. Bui,[1] James Favell,[1] Elena N. Kitova,[1] Zhixiong Li,[1] Kelli A. McCord,[1] Edward N. Schmidt[1], Fahima Mozaneh,[1] Mohamed Elaish,[2,3] Amr El-Hawiet,[4] Yves St-Pierre,[5] Tom C. Hobman,[2,6,7] Matthew S. Macauley,[1,6] Lara K. Mahal,[1] Morris R. Flynn,[8] and John S. Klassen[1]*

[1]*Department of Chemistry, University of Alberta, Edmonton, Alberta, Canada T6G 2G2*

[2] *Department of Cell Biology, University of Alberta, Edmonton, AB, Canada T6G 2H7*

[3] *Poultry Diseases Department, Faculty of Veterinary Medicine, Cairo University, Giza, Egypt*

[4]*Department of Pharmacognosy, Faculty of Pharmacy, Alexandria University, Alexandria, Egypt*

[5] *Institut National de la Recherche Scientifique (INRS), INRS-Centre Armand-Frappier Santé Biotechnologie, Laval, QC, Canada H7V 1B7*

[6] *Department of Medical Microbiology and Immunology, University of Alberta, Edmonton, AB, Canada T6G 2E1*

[7]*Li Ka Shing Institute of Virology, University of Alberta, Edmonton, Alberta, Canada. T6G 2E1*

[8] *Department of Mechanical Engineering, Faculty of Engineering, University of Alberta, Edmonton, Alberta, Canada T6G 1H9*

* Corresponding Author:

Department of Chemistry

University of Alberta

Edmonton, AB CANADA T6G 2G2

Email: john.klassen@ualberta.ca

Telephone: (780) 492-3501

https://orcid.org/0000-0002-3389-7112

Mathematical model for mixing of two solutions in nanoESI emitter

To provide a theoretical framework to interpret the experimental results, we consider the idealized one-dimensional problem of diffusion in a long capillary of length L and (constant) diameter d – see Figure S2-1. Mimicking experimental conditions, we allow the solute to be transported both by diffusion and also by advection, though we assume that the latter effect is small in a way to be quantified later. Using subscripts to indicate partial differentiation with respect to time (t) and space (x), the equation governing the spatio-temporal evolution of the solute concentration, c, is

$$c_t + uc_x = Dc_{xx}\,, \tag{1}$$

where D is the diffusion coefficient and u is the advection velocity, which, for analytical convenience, we assume to be constant. Note that $u = Q/(\pi d^2/4)$ in which Q is the volumetric flow rate of the liquid through the capillary. It is advantageous to non-dimensionalize eq 1 by introducing non-dimensional variables for space ξ ($x = \xi L$) and time τ ($t = \tau L^2/D$). Then we can rewrite eq 1 as

$$(c/c_0)_\tau + \mathrm{Pe}(c/c_0)_\xi = (c/c_0)_{\xi\xi}\,, \tag{2}$$

in which c_0 is the initial solute concentration as measured in the region $x < \ell$ where the horizontal distance ℓ is defined in Figure S2-1. The Peclet number, Pe, is defined as $\mathrm{Pe} = uL/D$.

Eq 2 makes reference to the non-dimensional concentration c/c_0, however, it is advantageous to consider the evolution of the non-dimensional concentration deficit, defined as $\sigma = 1 - c/c_0$. Whereas σ solves the same governing equation as c/c_0, i.e.

$$\sigma_t + \mathrm{Pe}\,\sigma_\xi = \sigma_{\xi\xi}\,, \tag{3}$$

we can transform the above equation into a standard heat equation by applying the transformation

$$\sigma(\xi,\tau) = \exp\left[\frac{1}{2}\mathrm{Pe}\ (\xi - \frac{1}{2}\mathrm{Pe}\tau)\right]\upsilon(\xi,\tau)\ , \tag{4}$$

such that υ satisfies

$$\upsilon_\tau = \upsilon_{\xi\xi} \tag{5}$$

Boundary conditions relevant to the above governing equations are as follows. At x = 0, ξ = 0, i.e. on the left-hand side of the capillary,

$$c(0,t) = c_0 \quad \Rightarrow \quad \sigma(0,\tau) = 0 \quad \Rightarrow \quad \upsilon(0,\tau) = 0. \tag{6}$$

At $x = L$, ξ = 1, i.e. on the right-hand side of the capillary,

$$c_x(L,t) = 0 \quad \Rightarrow \quad \sigma_\xi(1,\tau) = 0 \quad \Rightarrow \quad \upsilon_\xi(1,\tau) + \frac{1}{2}\mathrm{Pe}\ \upsilon(1,\tau) = 0. \tag{7}$$

Also, at t = 0, $\boldsymbol{\tau}$ = 0,

$$c = c_0\left[1 - \mathrm{H}(x - l)\right] \ \Rightarrow \ \sigma(\xi,0) = \mathrm{H}(\xi - \frac{l}{L}) \ \Rightarrow \ \upsilon(\xi,0) = \exp(-\frac{1}{2}\mathrm{Pe}\xi)\mathrm{H}(\xi - \frac{l}{L}), \tag{8}$$

where H denotes the Heaviside step function.

To solve eq 5, we assume a separable solution of the form

$$\upsilon(\xi,\tau) = X(\xi)T(\tau). \tag{9}$$

Substitution of eq 9 into eq 5 shows that

$$X_n(\xi) = A_n\cos\lambda_n\xi(\tau) + B_n\sin\lambda_n\xi(\tau), \tag{10}$$

where λ_n is the sequence of eigenvalues with n = 1, 2, 3 and A_n and B_n are integration constants. Subsequent application of the left- and right-hand side boundary conditions confirm that $A_n = 0$ whereas the eigenvalues satisfy a transcendental equation of the form

$$\tan \lambda_n = -\frac{\lambda_n}{\frac{1}{2}\mathrm{Pe}} \tag{11}$$

The first (trivial) root of eq 11 is $\lambda_1 = 0$ whereby $\sin \lambda_1 \xi = 0$. Thereafter, and to good approximation for small Pe,

$$\lambda_2 \approx \frac{\pi}{2} \qquad \lambda_3 \approx \frac{3\pi}{2} \qquad \lambda_4 \approx \frac{5\pi}{2}. \tag{12}$$

In the interests of maintaining an orthogonal set of basis functions, we consider as a generic solution for X_n:

$$X_n(\xi) = B_n \sin\left[\left(n - \frac{1}{2}\right)\pi\xi\right], \qquad n = 1, 2, 3... , \tag{13}$$

such that

$$T_n(\tau) = C_n \exp\left[-\left(n - \frac{1}{2}\right)^2 \pi^2 \tau\right]. \tag{14}$$

Combining eq 13 and eq 14 and using superposition yields the small-Pe solution for υ

$$\upsilon(\xi,\tau) = \sum_{n=1}^{\infty} b_n \sin\left[\left(n - \frac{1}{2}\right)\pi\xi\right] \exp\left[-\left(n - \frac{1}{2}\right)^2 \pi^2 \tau\right] \tag{15}$$

where the Fourier coefficients b_n satisfy

$$b_n = 2\int_0^1 \exp(-\frac{1}{2}\mathrm{Pe}\xi)\sin\left[\left(n-\frac{1}{2}\right)\pi\xi\right]\mathrm{H}\left(\xi-\frac{l}{L}\right)\mathrm{d}l. \qquad (16)$$

Using the above solution for υ, eq 4 can be solved for σ and the normalized concentration $c/c_0 = 1 - \sigma$ can be found.

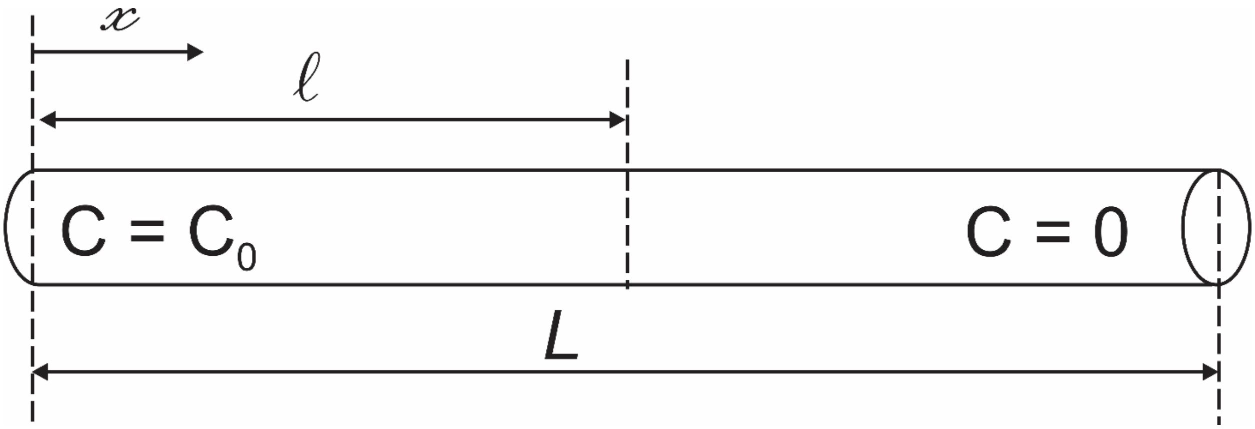

Figure S2-1. Capillary tube schematic. We assume that the solute (whose initial concentration in the region $x < l$ or $\xi < l/L$ is c_0) is transported by a combination of diffusion and (slow) left-to-right advection.
